# Targeting immunosuppressive myeloid cells via implant-mediated slow release of small molecules to prevent glioblastoma recurrence

**DOI:** 10.1038/s41551-025-01533-2

**Published:** 2025-10-22

**Authors:** Yannik Kaiser, Christopher S. Garris, Eliana Marinari, Hyung Shik Kim, Juhyun Oh, Martin Pedard, Elias A. Halabi, Moonhyun Choi, Sepideh Parvanian, Rainer Kohler, Denis Migliorini, Ralph Weissleder

**Affiliations:** 1 Center for Systems Biology, Massachusetts General Hospital, Boston, MA USA; 2 Department of Pathology, Harvard Medical School, Boston, MA USA; 3 Department of Oncology, University Hospital of Geneva, Geneva, Switzerland; 4 Center for Translational Research in Onco-Hematology, University of Geneva, Geneva, Switzerland; 5 Brain Tumor and Immune Cell Engineering Laboratory, AGORA Cancer Research Center, Lausanne, Switzerland; 6 Swiss Cancer Center Léman (SCCL), Geneva, Switzerland; 7 Department of Systems Biology, Harvard Medical School, Boston, MA USA

**Keywords:** Cancer immunotherapy, Immunotherapy, Drug delivery, CNS cancer, Cancer microenvironment

## Abstract

Glioblastoma is a highly aggressive brain tumour with a high risk of recurrence after surgery, even when combined with chemotherapy and radiotherapy. A major barrier to lasting treatment is the tumour’s immunosuppressive environment, which is largely dominated by myeloid cells. Here we describe the development of a biodegradable implant to sustainably release immune-modulator small molecules to reprogram tumour-infiltrating myeloid cells toward a pro-inflammatory, antitumour phenotype in the surgical cavity after tumour removal. In immunocompetent mouse models, this therapy induces interleukin-12 expression in myeloid cells without systemic cytokine elevation, and increases the infiltration of CD8^+^ and CD4^+^ T cells. Over 50% of mice treated (in combination with radiotherapy and chemotherapy) remain tumour-free during the experimental course (80 days). We further treated human glioblastoma explants ex vivo with the therapy and observed increased interleukin-12 expression in tumour-infiltrating myeloid cells, supporting the translational potential of this strategy. This implantable system offers a promising approach to prevent glioblastoma recurrence by activating innate immunity and sustaining immune surveillance post-surgery.

## Abstract (web summary)

A biodegradable implant inserted after tumour removal sustainably releases immune modulators and reprograms tumour-infiltrating myeloid cells toward a pro-inflammatory, antitumour phenotype, offering a promising therapeutic strategy for preventing glioblastoma recurrence.

## Main

Recurrent high-grade glioma (glioblastoma, GBM) is the most common and lethal primary malignant cancer of the central nervous system. Standard-of-care therapies include surgical resection with chemoradiation and other US Food and Drug Administration (FDA)-approved treatments such as intracavitary biodegradable implants for the release of chemotherapy (wafers), bevacizumab and alternating electrical fields^1^. Immune-based treatments have also received attention, but, unfortunately, the high rate of failure in clinical GBM trials continues^2–6^. Tumour myeloid-derived cells and resident microglia are the most abundant non-neoplastic cell types, often accounting for about 20–40% of the tumour mass^7,8^ and contributing to the highly immunosuppressive microenvironment^9^.

Several myeloid-targeted therapies have been proposed, including CSFR1 inhibition^10^, disruption of the CD47–SIRPα axis^11,12^, chimeric antigen receptor (CAR)-macrophages^13^ or different types of interleukin-12 (IL-12) therapies^14^. The latter has emerged as an attractive cytokine for cancer therapy, because it has potent anticancer effects^15^ and additionally plays a critical role in enhancing checkpoint inhibitors^16^. In short, IL-12 elicits broad antitumour effects in multiple cancer models; acts on various immune cell types, including myeloid cells, natural killer (NK) cells, B cells and T cells; turns on signalling pathways that aid in the effector activation of T cells^16–19^; and can be elicited by activating both canonical and NF-κB pathways, especially through inhibition of the cellular inhibitor of apoptosis protein-1 (cIAP)^20,21^. Clinical trials of systemic IL-12 have been undertaken but had to be terminated because the cytokine, administered as a recombinant soluble protein, was poorly tolerated^22^. As a result, attention has turned to increasing local concentrations of IL-12, bypassing systemic side effects^23^. This has included, for example, the delivery of *IL12* mRNA^24^, IL-12 heterodimers fused to a monoclonal antibody targeting DNA in necrotic tumour regions (M9241)^25^ and a ligand-inducible system^14,26^. The IL-12 delivery/induction with these methods was moderate and temporally limited, necessitating alternative approaches. One alternative to the above has been systemically injecting nanoparticles loaded with NF-κB modulators to trigger the native production of IL-12 in the tumour microenvironment (TME)^27,28^. Although this approach worked well in peripheral cancers, the blood–brain barrier (BBB) in central nervous system tumours continues to be a considerable delivery challenge^29^.

Administration of therapeutics directly into the surgical resection cavity can bypass some delivery issues, and various slow-release wafer materials with different antitumour payloads have been suggested. Unfortunately, the survival advantages with these approaches are only marginally better than surgery alone. Therefore, there is a need to develop more effective systems, and one approach is the long-term modulation of the immunosuppressive myeloid glioblastoma microenvironment. In this Article we report on a guest–host macrostructure material for regional myeloid conversion to an immunostimulatory, antitumour phenotype mediated through canonical and non-canonical NF-κB pathway activation as well as STAT3 pathway silencing. We found that this surgical approach alone led to long-term survival, without tumour recurrence, in approximately half of the animals with orthotopic GBM. When combined with standard-of-care radiation and temozolomide, an even higher fraction of animals remained GBM-free. These results present promising potential for a new treatment for GBM.

## Results

### Analysis of the myeloid cell compartment in murine and human GBM

**Fig. 1:**
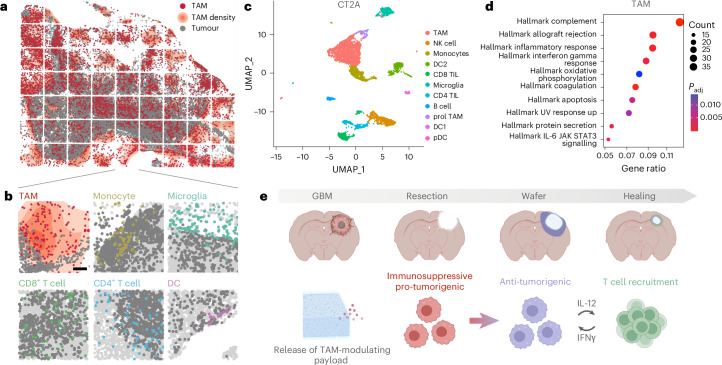
Glioblastoma is rich in immunosuppressive TAMs. **a**,**b**, Synthetic spatial maps of myeloid cell distribution from the CT-2A model. The white grids represent 63 separate fields of view (FOVs) imaged to acquire the entire tissue specimen. Note that ~25% of all cells in CT-2A GBM represent macrophages. **b**, High-resolution maps showing the distribution of different immune cells in a GBM specimen (each FOV is 512 µm × 512 µm; scale bar, 100 µm). DC, dendritic cells. **c**, Single-cell RNA-sequencing data from the CT-2A dataset^30^, showing the prominence of the macrophage/monocyte pool. pDC, plasmacytoid DC; TIL, tumour-infiltrating lymphocytes. **d**, Geneset enrichment analysis: the CT-2A TAM pool is characterized by high NF-κB signalling and hypoxia, contributing to the highly immunosuppressive environment (also Supplementary Fig. 1). Statistical significance was calculated using a hypergeometric test (Fisher’s exact test) with *P* values adjusted for multiple testing using the Benjamini–Hochberg method. **e**, Schematic overview of the study. Intracranially implanted murine GBM tumours were surgically resected, and the resection cavity was filled with a triple-drug-loaded CANDI wafer. The release of the wafer payload converts the immunosuppressive pro-tumorigenic myeloid compartment into an antitumour one (‘myeloid immunoconversion’). The converted myeloid cells produce cytokines that result in T cell recruitment (via IL-12, CCL5 and others) and further antitumour effects. Panel **e** created with BioRender.com. Source data

We initially performed a bioinformatic analysis to determine the proportion and composition of the myeloid compartment in GBM (Fig. 1 and Supplementary Fig. 1) based on published datasets^30,31^. Through this and spatial profiling of murine GBM, we found that macrophages and monocytes make up ~25% of all cells in the mouse CT-2A GBM model. The macrophages are characterized by immunosuppressive markers such as SPP1, and major pathway hallmarks include NF-κB signalling. Similar findings were identified in human GBM (Supplementary Fig. 1), albeit with certain differences. Although the immunosuppressive macrophage compartment was still large, the microglial compartment was also sizable. Different myeloid cell therapeutics are being evaluated^32^. One newer approach has been the systemic use of macrophage-targeting nanomaterials capable of immune modulation and efficient drug delivery to macrophages, which has been tested in various malignancies and shown potent antitumour effects^27^. However, as newly diagnosed GBM tumours are routinely surgically resected, we have designed a wafer system that could be implanted during surgery and locally release myeloid immunostimulatory compounds over prolonged periods.

We created an implantable crosslinked bis-succinyl cyclodextrin material that serves as a ‘sponge’ to hold immunostimulatory small molecules. We hypothesized that the release of these molecules over time would lead to high local IL-12 production in macrophages, known to trigger prominent antitumour effects^27,28^ (Supplementary Fig. 2). However, it was unclear whether this strategy would merely delay GBM recurrence or result in cures. Furthermore, although there are data on the systemic distribution of nanometre-sized materials^27^, it was unclear what the effects and distribution of the degradable wafer material and its payload would be in murine tumours.

### Synthesis and characterization of the immune-stimulatory CANDI wafer

**Fig. 2:**
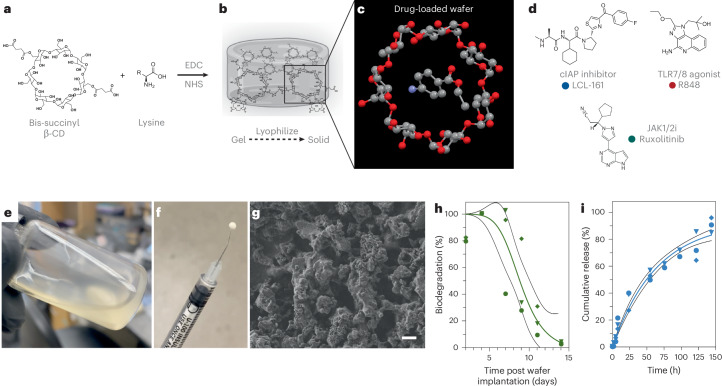
CANDI wafer is a cyclodextrin-based host–guest macrostructure enabling local delivery of immunostimulatory small molecules in the GBM resection cavity. **a**,**e**, bsCD was crosslinked with lysine (**a**) to yield a gel-like material (**e**) that was subsequently lyophilized into implantable solid but porous wafers. **b**,**d**, Cyclodextrin units in the wafer act as host–guest units (**b**) and are loaded with immune-stimulatory small molecules (for example, JAKi, cIAPi and TLR7/8 agonist), which stimulate IL-12 production in TAMs^27^. **e**, Gel-like core material following crosslinking. **f**, Lyophilized gel material ready for implantation into the GBM resection cavity. **g**, Scanning electron microscopy of the wafer surface, showing a porous solid material (in-depth SEM characterization is provided in Supplementary Figs. 3 and 4). Scale bar, 100 µm. **h**, MRI was used to monitor the in vivo degradation of covalently Gd-labelled wafer material (*n* = 3 biological replicates). **i**, Release rates of the small-molecule compounds from the wafer material (*n* = 3 independent replicates, represented by different symbols). The release reaches a maximum within ~7 days (details are provided in Supplementary Fig. 5). Additional release in vivo depends on the degradation of the wafer material, which occurs over several days (Supplementary Fig. 6). Panel **b** created with BioRender.com. Source data

Previously, bis-succinyl β-cyclodextrin (bsCD) had been crosslinked with lysine, yielding a nanoparticle 27 nm in size that was loaded with a dual immunostimulatory payload^28^. The reaction conditions and concentrations were adjusted to yield a thick gel-like material (Fig. 2) that was subsequently dialysed against a 4-kDa molecular mass cutoff and then loaded with three immune-modulatory small molecules. The three molecules included a Janus tyrosine kinase (JAK) inhibitor (ruxolitinib), a cIAP inhibitor (LCL-161) and a Toll-like receptor TLR7/8 agonist (R848). This triple combination was identified by a drug screen to maximize IL-12 production in myeloid cells when given systemically^27^. Additional earlier work had identified R848 and LCL-161 as being able to modulate tumour-associated macrophage (TAM) phenotypes when used as monotherapy^33^ or dual therapy^21^ in peripheral cancers. Unlike in previous work, however, here we did not use systemically administered nanomaterials, as the BBB would form a considerable delivery barrier. Instead, we developed a more highly crosslinked material for prolonged degradation/release and pressed the triple-drug-loaded material into implantable wafer shapes. Each implantable wafer weighed ~10 mg (volume ~4–6 mm^3^) in its dried form and contained 0.22 mg of the active drug substance (0.04 mg R848, 0.1 mg LCL-161 and 0.08 mg ruxolitinib).

Figure 2e and Supplementary Figs. 3 and 4 provide an overview of the material surface as determined by scanning electron microscopy (SEM). The pressed wafer material has a porous surface reminiscent of a sponge. We determined the drug-release rates of the small molecules from the wafer material. For these studies, we stored drug-loaded wafers in dialysis tubing and determined the release of individual drugs as a function of time. Figure 2i and Supplementary Fig. 5 summarize the results from these studies. In this closed in vitro system, the half-life of release was ~45 h. At 144 h, only 8% (range 3.7–13.2%) of the drug remained in the wafer material, as determined by trypsinization experiments. To supplement our understanding of degradation, we also performed in vivo experiments to understand how proteases in the TME may play additional roles in biodegradation. For these studies, we covalently attached gadolinium-tetraazacyclododecanetetraacetic acid (Gd-DOTA) to cyclodextrin units, implanted these ‘MRI wafers’ in GBM resection cavities, and performed serial magnetic resonance imaging (MRI) on the animals (Fig. 2h and Supplementary Fig. 6). These studies showed that the wafer material is degraded in vivo with a half-life of 8.9 days.

### Cellular effects of the CANDI wafer

**Fig. 3:**
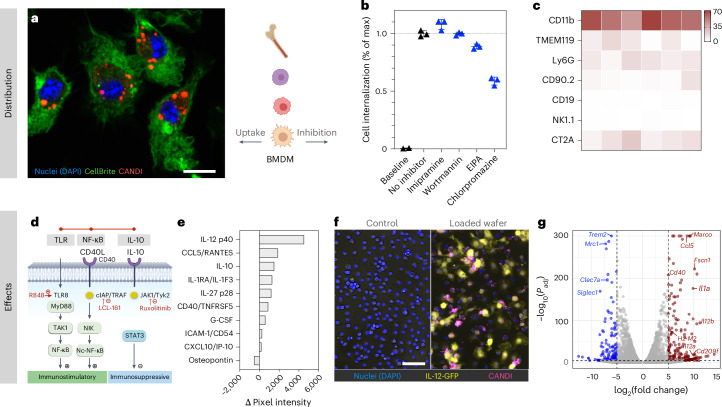
CANDI wafer is internalized into TAMs and reprograms them towards a pro-inflammatory phenotype. **a**, To determine the cellular uptake of wafer material, BMDMs were differentiated into macrophages using macrophage colony-stimulating factor (M-CSF). After seven days, macrophages were incubated with 0.01 mg of wafer material stained with AF647 for 24 h, and the cells were imaged by confocal microscopy. Note the uptake of wafer material into vesicular structures, presumably perinuclear lysosomes. DAPI, blue; wafer, red; CellBrite membrane stain, green. Scale bar, 10 µm. A colourblind-accessible version of this image is provided in Supplementary Fig. 23. Representative images are from one of three technical replicates with similar results. **b**, Mechanism of cellular CANDI uptake. iMAC cells were incubated with different uptake inhibitors, and the cellular uptake of CANDI-AF647 was assessed using flow cytometry. Chlorpromazine inhibits clathrin-mediated endocytosis (Rho-GTPase). Wortmannin inhibits micropinocytosis/phagocytosis (PI3K). Imipramine inhibits macropinocytosis. EIPA inhibits macropinocytosis via Na/H exchange. The biggest effects were observed with chlorpromazine (*n* = 3 technical replicates; details are provided in Supplementary Fig. 8). Data are presented as mean ± s.d. **c**, Summary of TME cells staining positive for wafer material. Each column represents a mouse. Note that the material is mostly present in CD11b-positive TAMs as opposed to other cells. **d**, Schematic overview of the effects of the triple drug on myeloid function. The combined effect is an immunostimulatory one. **e**, Cytokine induction in BMDMs exposed to drug-loaded CANDI wafer as determined by enzyme-linked immunosorbent assay (ELISA). Note the high expression of IL-12 and CCL5, and the downregulation of SPP1^35^. Data are shown as Δ pixel intensity (arbitrary units) compared to PBS control. **f**, Prominent in vitro induction of IL-12 in BMDM cells. Scale bar, 50 µm. Representative images are from one of three technical replicates with similar results. **g**, Bulk RNA-seq upregulation of *Il12*, *Ccl5*, *Marco* and other immunostimulatory genes. Note the downregulation of *Trem2*, *Mrc1* and *Clec7a*. Statistics were generated with the Wald test with Benjamini–Hochberg FDR correction. Panels **a** and **d** created with BioRender.com. Source data

We performed a series of in vitro and in vivo studies to determine the pharmacokinetics and pharmacodynamics of the wafer and its payload. First, we determined whether the wafer material was internalized into macrophages or whether the small-molecule payloads exerted their effects without bulk internalization. Bone marrow-derived macrophages (BMDMs) were incubated with fluorescently labelled CANDI-AF647 wafer and imaged by microscopy (Fig. 3a). These data clearly show cellular internalization of the wafer material, data that were further corroborated by flow cytometry (Fig. 3b,c). Using confocal imaging, we determined the uptake of CANDI into punctuate intracellular structures within 24 h, co-localized with Lysotracker stains. At later time points, there was cytoplasmic localization of the fluorescent CANDI (Supplementary Fig. 7), a finding also observed with other cyclodextrin nanomaterials^34^. Similar experiments were also conducted in vivo using MerTK–GFP mice (Fig. 5b and Supplementary Fig. 11). These cell-internalization effects were most pronounced 3–5 days after implantation. We next determined the mechanism of cellular CANDI wafer uptake using typical inhibitors of different pathways (chlorpromazine for clathrin-mediated endocytosis; wortmannin for micropinocytosis/phagocytosis; imipramine for macropinocytosis; EIPA (5‐(*N*‐ethyl‐*N*‐isopropyl)amiloride) for macropinocytosis via Na/H exchange) (Fig. 3b and Supplementary Fig. 8). The biggest inhibitory effects in uptake were observed with chlorpromazine, arguing for uptake via clathrin-mediated endocytosis. Additionally, we noticed that *Marco* and *Cd209* were highly upregulated and could be CANDI entry points.

To determine the effects of the wafer payload on different TAM pathways (Fig. 3d), we performed cytokine profiling, imaging of IL-12 reporter cells and RNA-seq. Cytokine profiling of wafer-exposed BMDMs showed high expression of both IL-12a and IL-12b, CCL5 and other cytokines, as expected. Interestingly, we also saw downregulation of SPP1, a negatively associated TAM biomarker^35^. In BMDMs derived from IL-12–eYFP reporter mice, we observed massive IL-12 induction in virtually all cells upon wafer stimulation (Fig. 3f). Finally, bulk RNA-seq (Fig. 3g and Supplementary Fig. 9) of wafer-exposed macrophages provided a few interesting observations. First, when an empty wafer was used without a therapeutic payload, no genes were upregulated or downregulated, attesting to the inertness of the host material (Supplementary Fig. 9). When macrophages were exposed to triple-drug-loaded wafer material, we observed many upregulated (*n* = 177) and downregulated (*n* = 117) genes (Supplementary Fig. 9). Among the top upregulated genes were *Il12* (the main mechanism of action), *Marco* (encoding SCARA2, a scavenger receptor that assists in clearing cellular debris, being a co-receptor to TLR modulating the immune response), *Cd209* (encoding DC-SIGN, a C-type lectin receptor that facilitates the uptake of carbohydrate-rich microorganisms and materials) and *H2-M2* (involved in antigen presentation, MHC). Genes such as *Cd40* and *Fscn1*, both connected to non-canonical NF-κB activation, were also significantly increased by the drug-loaded wafer. Key downregulated genes included *Mrc1* (encoding CD206, typical M2 marker for mannose receptor), *Siglec1* (encoding CD169, anti-inflammatory roles in macrophages)^36^, *Trem2* (an M2 marker, also a drug target for TAMs)^37–39^ and *Clec7a* (encoding dectin-1, associated with poor survival, and blocking can enhance immunotherapy)^40,41^.

We also determined the toxicity of the wafer by means of a number of different experiments: (1) viability assays using immortalized macrophages and THP-1 cells (Supplementary Figs. 20 and 21); (2) blood count and clinical chemistry (Supplementary Table 1); (3) glial fibrillary acidic protein (GFAP) staining in surviving mice (Supplementary Fig. 15); and (4) systemic IL-12 level measurements (Fig. 5d). All of these results were normal and showed no indications of local or systemic toxicity. By contrast, free drugs at similar doses are hepatotoxic, as previously noted^27,42,43^.

### Antitumour efficacy as mono and combination therapies

**Fig. 4:**
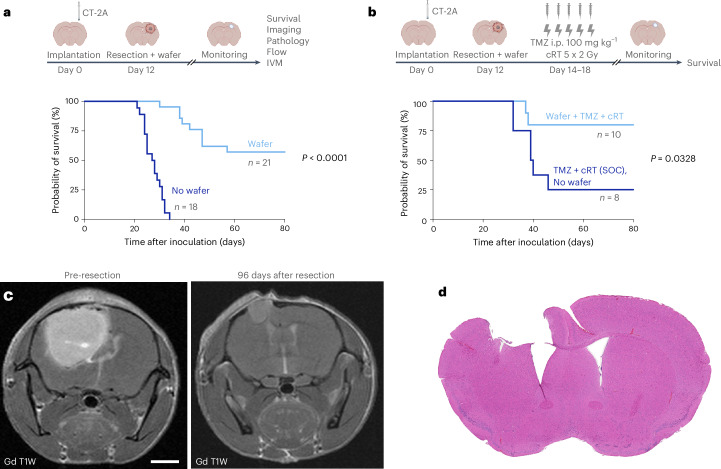
Therapeutic efficacy—intracavitary immunoconversion improves long-term survival and synergizes with standard-of-care treatment in GBM. **a**, Study overview. Following implantation, GBM tumours were resected, and wafers were implanted on day 12. Tumours were monitored by serial bioluminescence and/or MRI up to 96 days after tumour implantation. Tumours were processed for histology, flow cytometry and other studies at various times after resection. The dark blue line in the surgical curve reflects animals (*n* = 18) whose GBM tumours were surgically resected without wafer implantation. The light blue curve reflects resected GBM tumours with wafer implantation (*n* = 21). Eighty days after tumour inoculation, approximately half of the animals were alive and appeared functionally intact. *P* = 0.00000000007, log-rank test. **b**, As in **a**, but with additional combination therapies, including radiation and temozolomide. cRT, chemoradiotherapy; SOC, standard of care. Note the higher survival. *P* = 0.0328, log-rank test. Additional statistics are provided in Supplementary Table 2. **c**, MRI typically shows a residual surgical cavity filled with fluid but completely absent of tumour (as determined by histology). Scale bar, 2 mm. **d**, Haematoxylin and eosin stain of representative brain slice. There is no residual tumour nor an immune infiltration at the site of the previous GBM. GFAP staining is shown in Supplementary Fig. 14. Panels **a** and **b** created with BioRender.com. Source data

Having determined the molecular and cellular effects of the CANDI wafer, in vitro and in vivo, we next set out to determine efficacy in a glioma resection model. As outlined in Fig. 4, GBMs were resected, and the resection cavity was either left alone (control, *n* = 18) or filled with drug-loaded wafers (*n* = 21). Animals were then serially monitored by imaging (MRI, Supplementary Fig. 12; bioluminescence imaging, Supplementary Fig. 13) and for overall survival. All control CT-2A-bearing mice with resected tumours died within 23 days of surgery (35 days after tumour implantation). This outcome is similar to the clinical scenario, where resection alone cannot achieve tumour control. Conversely, when the resection cavity was packed with the drug-loaded CANDI wafer, over half of the animals were alive at day 96, the longest time point investigated. The difference in survival between the groups was highly statistically significant (*P* < 0.0001; Fig. 4a and Supplementary Table 2). At that time, all surviving animals showed normal grooming behaviour and appeared healthy. MRI at that time point showed no enhancing tumour, but merely a fluid-filled resection cavity (Supplementary Fig. 12). The decision was made to euthanize the animals at this point and investigate whether there were any residual microscopic tumour deposits left. Autopsies in these animals showed a fluid-filled resection cavity, and haematoxylin and eosin staining confirmed the complete absence of any residual tumour (Supplementary Fig. 14).

We also determined whether the CANDI wafer-induced immunotherapy could be further enhanced by conventional standard-of-care therapy. We thus combined the surgical resection with wafer placement, radiation and temozolomide treatments (*n* = 18), as is done routinely clinically. Our data show that the treatment effects are indeed synergistic, with nearly 70% of animals surviving 80 days, demonstrating a statistically significant difference between the CANDI-treated and untreated groups (*P* = 0.0328; Fig. 4b and Supplementary Table 2). Finally, we tested some of the above in a second GBM model using SB-28 cells, a much more invasive and aggressive model (*n* = 23). Again, we observed a highly statistically significant difference in survival (*P* = 0.0007) and a similar therapeutic phenotype with nearly 25% survival at *t* = 80 days (Supplementary Fig. 16 and Supplementary Table 2).

### CANDI wafer induces IL-12- and CD8-mediated tumour control

**Fig. 5:**
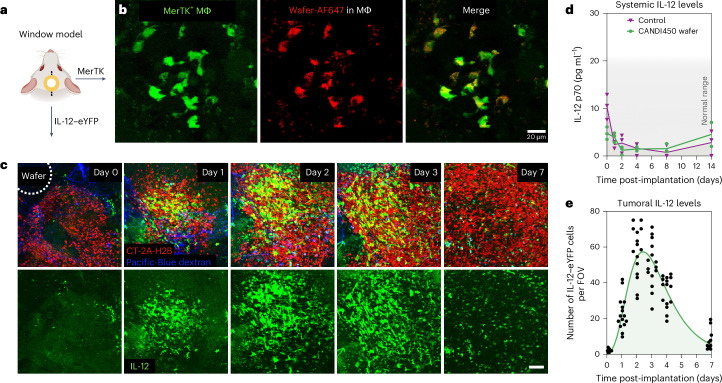
Serial intravital imaging following CANDI wafer implantation reveals macrophage uptake and transient IL-12 induction without systemic elevation. **a**, Brain windows were implanted into mice to allow for serial imaging of tumour growth, resection and monitoring of drug effects of the implanted CANDI wafers. **b**,**c**, Experiments were performed in MerTK–GFP (**b**) and IL-12–eYFP mice (**c**). **b**, Three days after AF647-stained wafer implantation, wafer material could be detected in MerTK–GFP-positive macrophages (MØ) that had accumulated at the periphery of the wafers (Supplementary Fig. 11 provides details). Scale bar, 20 µm. **c**, IL-12–eYFP induction in the tissue surrounding the wafer was measured serially over seven days using an IL-12–eYFP reporter mouse. The highest IL-12 concentrations occur between one and five days after implantation. Of note is the fact that only microwafers and not full-sized ones could be implanted under the window, leading to a faster degradation time. Pacific Blue dextran was administered intravenously. Scale bar, 100 µm. In **b** and **c**, serial imaging was performed over four (**b**) or seven (**c**) days in one animal each, and multiple FOVs were analysed, yielding similar results. Colourblind-accessible versions of these images are provided in Supplementary Fig. 23. **d**, Note that the locally high IL-12 levels do not cause an increase in systemic IL-12 levels. The grey shaded area represents the normal level. *n* = 3 biological replicates. Data are presented as mean ± s.d. **e**, Quantitation of IL-12–eYFP levels in the window model. Note that the wafer in the window model was much smaller, so the IL-12 induction effects are shorter than those of the GBM resection model. Panel **a** created with BioRender.com. Source data

**Fig. 6:**
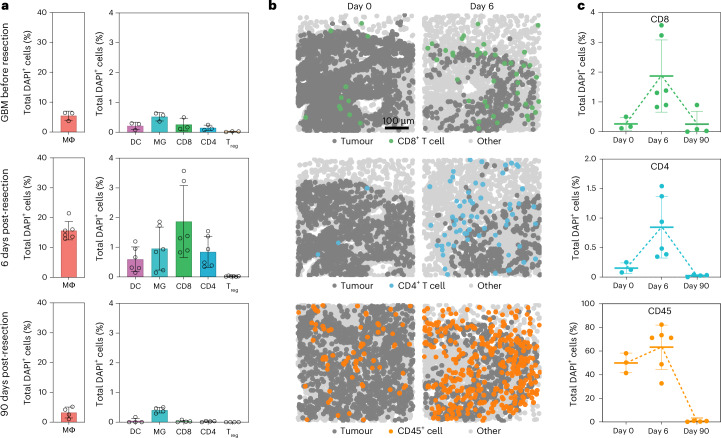
Temporal immune cell analysis of the TME shows transient myeloid expansion as well as CD4/CD8 T cell recruitment. **a**, Immunohistochemistry on native CT-2A tumours (top; *n* = 3 biological replicates), six days after subtotal resection and wafer implantation (middle; *n* = 6 biological replicates) and 90 days after surgery (bottom; *n* = 4 biological replicates). Analysis shows abundant macrophages in the TME, whereas other immune cells are scant, consistent with the known immunosuppressive environment. Following resection and wafer implantation, the myeloid cell compartment increases, and so does CD8 and CD4 recruitment. Ninety days after surgery, there are hardly any immune cells remaining at the prior GBM site. MG, microglia; T_reg_, regulatory T cells; **b**, Representative synthetic images corresponding to the three different conditions and two different time points (FOV, 512 µm × 512 µm). Scale bar, 100 µm. **c**, Temporal analysis of CD8, CD4 and total immune cells over time (day 0, *n* = 3 biological replicates; day 6, *n* = 6 biological replicates; day 90, *n* = 4 biological replicates). In **a** and **c**, data are presented as mean ± s.d. For immune cell composition in other treatment groups, see Supplementary Fig. 19. Source data

To interrogate the mechanism by which the CANDI wafer works in vivo, we performed serial intravital microscopy experiments (Fig. 5), spatial biology experiments (Fig. 6 and Supplementary Fig. 19) and flow cytometry (Supplementary Figs. 17 and 18). Serial intravital microscopy using the brain window chamber model was performed following the implantation of microsized wafers under the window. Given the tight space below the window, these wafers were much smaller than the post-surgically implanted ones and were thus resorbed faster. Serial imaging showed a strong local IL-12 response lasting for the duration of the micronized wafer (4–5 days). High local IL-12 concentrations in the brain (Fig. 5e) did not elevate systemic IL-12 levels (Fig. 5d), unlike systemic IL-12 therapies^22^. Flow cytometry of resected tumour residuals after CANDI wafer treatment (Supplementary Fig. 18) showed a significant increase in the expression of CD86 (*P* = 0.0182) and MHC-II (*P* = 0.0019) in F4/80^high^ macrophages, indicative of TAM polarization towards a pro-inflammatory phenotype. We also observed an immune-suppressive, wound-healing macrophage phenotype (high CD206 and TREM2 expression) in mice that underwent surgery but had no CANDI wafer implantation. In mice that received CANDI wafer treatment, expression levels of CD206 (*P* = 0.0092) and TREM2 (*P* = 0.001) were significantly lower in macrophages.

Immune cell profiling by immunohistochemistry (Fig. 6 and Supplementary Fig. 19) of native CT-2A cells showed a predominately TAM-rich tumour environment essentially largely devoid of CD8 cells. Following subtotal surgical resection and wafer implantation, we noted a significant increase in tumoral CD8 (7.2-fold; *P* = 0.0258) and CD4 (5.6-fold; *P* = 0.0282) levels, which was not observed with surgical resection alone. Temporal analysis of different cohorts also showed a three-fold increase in TAM (*P* = 0.0017) and a 1.3-fold increase in CD45^+^ cells in the CANDI wafer cohort, which returned to baseline levels (*P* < 0.0001) after 90 days. Finally, we performed flow cytometry to further confirm the histology results (Supplementary Fig. 17). These data showed a 4.7-fold increase in CD8 (*P* = 0.0094) and a 2.3-fold increase in CD4 (*P* = 0.0413) recruitment in wafer-treated animals. Finally, we show by flow cytometry that the wafer material indeed primarily accumulated in TAM cells (Fig. 3c).

Finally, to assess whether wafer treatment induced antitumour immunity, we rechallenged previously cured mice in whom GBM tumours were resected (Supplementary Fig. 22). By day 22 after the rechallenge, three of four mice in the wafer group remained completely tumour-free, and one exhibited minimal tumour growth. By contrast, all control mice that received de novo flank tumour implantation developed significant tumour burden, suggesting that wafer immunoconversion generates a lasting immune response capable of preventing tumour recurrence.

### CANDI wafer has immune-stimulatory effects in human GBM tissue

**Fig. 7:**
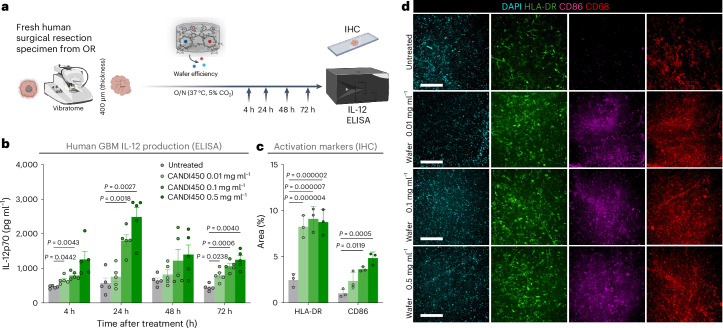
CANDI wafer elicits dose-dependent IL-12 induction and myeloid activation in human GBM. **a**, Fresh resected human GBM tissue was cut into 400-µm-thick sections using a vibratome. The tissue slices were then incubated with CANDI wafer material. IHC, immunohistochemistry; O/N, overnight; OR, operating room. **b**, IL-12 induction was measured as a function of dose and time (*n* = 5). Note the highly significant IL-12 induction in human GBM tissue within 24 h (*P* < 0.001) and the dose dependency. **c**,**d**, Immunohistochemistry performed in the same sections (*n* = 3) showed upregulation of the myeloid cell activation markers HLA-DR, CD68 and CD86. One-way ANOVA with multiple comparisons was used to determine statistical significance. Scale bar, 100 µm. In **b** and **c**, *n* refers to individually incubated tissue sections from the freshly obtained human GBM sample. The experiment was repeated independently with GBM tissue from a second patient, yielding similar results. For in vitro wafer effects on human monocyte/macrophage THP-1 cells, as well as toxicity testing, see Supplementary Fig. 20. Panel **a** created with BioRender.com. Source data

To determine whether the CANDI wafer would have similar immune-stimulatory effects in human tissue, we performed two additional experiments. First, we obtained freshly resected human glioblastoma tissue and exposed vibratome-cut organotypic tissue slices to different amounts of the wafer. Tissue culture supernatant was then tested for IL-12 production (Fig. 7b). The data show a clear dose- and time-dependent increase in IL-12 compared to control (*P* < 0.001 at 24 h). Similarly, we observed significant upregulation of the myeloid cell activation markers HLA-DR, CD68 and CD86 in wafer-exposed tissue sections (Fig. 7c,d). Finally, we tested the wafer material in THP-1 cells, showing similar IL-12 induction effects, myeloid cell activation markers and no discernible toxicity in these human cells (Supplementary Fig. 20).

## Discussion

Standard-of-care GBM therapies include surgical resection, radiation and chemotherapy^4^. Despite this multipronged approach, recurrences are frequent, and survival remains abysmal^44^. Various immunotherapies have shown limited effects but have not changed the overall outcome for patients^45^. Furthermore, immunomodulators (such as R848, LCL-161 and ruxolitinib) administered systemically as free drugs have largely failed for pharmacokinetic, pharmacodynamic or toxicity reasons^27,46–49^. There are several reasons for the extraordinary treatment resistance, the highly immunosuppressive microenvironment being an important one^14,45^. Myeloid cells in GBM rapidly adopt an immunosuppressive phenotype^30,31,50^. We reasoned that an immunomodulatory wafer material could be constructed to shift immune suppression to stimulation. In this Article we have shown the synthesis, characterization and therapeutic efficacy of this approach. Using two different mouse models, we show antitumour immune responses preventing postoperative glioma recurrence.

TAMs in GBM are the most common immune cell type, contributing to ~20–40% of the actual tumour mass^7,50^. The cells originate from two independent sources: bone marrow-derived monocytes and brain-resident microglia. Most adult microglia are yolk sac-derived, have longevity and limited self-renewal, and are mostly found at the tumour/parenchyma interface^51^. By contrast, inside GBM, the BBB is impaired, and the expression of monocyte chemoattractant proteins is increased. This leads to a large influx of bone marrow-derived monocytes and their differentiation into immunosuppressive TAMs, often in the perivascular niche, although different subtypes and programs are being recognized^50^. TAMs have been shown to promote GBM growth^52^ through positive feedback loops with tumour cells^53^. Conversely, it is also known that certain TAM subtypes are plastic^54^ and can acquire antitumour phenotypes^32^. The task then is to provide long-term stimuli so that recruited TAMs adopt an antitumour phenotype and prime the TME for more effective tumour control. Our wafer approach offers a therapeutically effective strategy to achieve this goal and eradicate GBM.

We chose a biodegradable wafer material to deliver small-molecule immunomodulatory drugs to affect TAM function. Unlike in previous work^27^, we wished to achieve much longer drug release and slower degradation to modulate TAM function during the prolonged post-surgical healing process. As such, we increased the crosslinking of the bsCD monomers to yield a gel-like substance that could be drug-loaded and then lyophilized into implantable wafers. The triple-drug payload (R848, LCL-161 and ruxolitinib) was chosen deliberately, as it has been shown to be much more effective^27^ compared to single-^55^ and dual-drug loads^28^. We also adjusted the payloads so that cIAP inhibitors would not cause local toxicity. Collectively, this resulted in an implantable material that degraded within a couple of weeks, releasing the triple small-molecule payload from the surface in a time-dependent fashion. We furthermore showed that the prolonged release of immune-modulatory drugs can reprogram TAMs to an immune-stimulatory phenotype and prevent post-surgical tumour recurrence. IL-12 produced by TAM and tumour antigen-presenting cells conditions cytolytic immunity and interferon gamma locally, which suppresses tumour regrowth. We also showed that key T cell co-stimulation pathways are enhanced by drug-loaded wafers, and these signals, combined with IL-12, bolster antitumour T cell responses. It is possible that the post-surgical recurrence observed clinically is in part driven by immune-suppressive mechanisms triggered by surgical trauma^56^. We similarly observed immune-suppressive macrophage phenotype induction in surgically resected but non-drug-treated animals. However, by contrast, we found that, as opposed to anti-inflammatory treatments, type 1 polarizing (TH1) cytokine induction inhibits tumour growth recurrence. Nonetheless, we similarly observed that promoting CD8 T cell immune surveillance is essential to halt tumour outgrowth. These data argue in favour of a model in which post-surgery macrophage phenotypes are important determinants of tumour recurrence.

The current study had some limitations, and these may represent avenues for future research. First, it remains unclear why some animals with glioblastoma were completely cured while others showed relapse. Although the mice used for studies are genetically homogeneous, some limitations still exist in complex in vivo animal models of cancer that could explain the variability in treatment responses seen. These sources of variation can include tumour location, differences in cell numbers of implantations, size at the start of treatment and the degree of tumour resection efficiency. Although we attempted to perform the microsurgery in an identical fashion in the more than 140 operated animals, residual tumours may always be a possibility. Precision in the surgical removal of tumours and the subsequent placement of immune-stimulatory wafers may be a key determinant of translational success as these intraoperative immunotherapies progress. Innovations such as fluorescence-guided surgery for more defined tumour resection and tracking of the immune-stimulatory material distribution to immune active sites, such as the dura and cervical lymph nodes, could be used to optimize the immune-activating impact of intraoperative biomaterials.

Second, it is possible that additional drug loads may be synergistic, but this will require extensive future screens. Two pieces of evidence suggest that the current efficacy could be improved. In one recent study, we showed that CXCL9Hi macrophages represent another major antitumour phenotype^35^ and that different types of small-molecule payload can achieve this phenotype^57^. At this point, however, we do not know if this is the case for GBM. In another study, we showed that TAMs can act as slow-release reservoirs of cytotoxic payloads^58^ and that additional release can be triggered by radiation^59,60^.

Third, the kinetics of GBM growth and requirements for drug release are different between mice and humans. To slow the degradation of the biomaterial and afford longer drug release for human applications, additional design factors may be required. It is well known that biodegradation of implantable materials and drug release can be modulated by crosslinker types, density^61,62^ and the design of prodrug payloads with longer release kinetics^63^. Furthermore, identifying the microanatomic biodistribution of immune-stimulating wafer materials in the immune hubs of the central nervous system, including the cervical lymph nodes and dura, is a future area of research that can provide a more thorough understanding of how these therapeutics function. In other cancer models, we have found that although polarizing macrophages locally can trigger strong antitumour functions, durable responses are linked to the trafficking of dendritic cells to draining lymphoid tissues. Determining whether and how wafer-based materials either passively enter or are actively trafficked into lymphoid tissues is our next step in advancing these intraoperative immunotherapies.

Fourth, although our data and model suggest the effective in vivo reshaping of the TAM population, it is entirely possible to use similar materials for the creation of glioma-specific CAR macrophages. Previous studies have shown the use of a nano-porter system to achieve this effectively and thus to prevent GBM relapse^13^. Our own studies in this arena involve the creation of analogous CANDI scaffold materials able to carry mRNA^64^ and thus allow vaccination strategies for neoantigen presentation^65,66^. These studies are currently ongoing.

In summary, we have established the myeloid compartment as a future drug target capable of preventing GBM recurrence if modulated appropriately. This work provides the rationale for more efficient immunotherapy in GBM and warrants clinical translation. Further studies are necessary before the proposed wafer can be considered for clinical trials.

## Methods

The research conducted for this Article complied with all relevant ethical regulations. The experiments were approved by the MGH Institutional Animal Care and Use Committee (IACUC) under protocol no. 2021N000135 and performed according to MGH IACUC regulations and the Human Research Ethics Committee from Geneva University in Switzerland (CCER 2022-02109 and 2023-01928).

### Materials

All reagents and solvents were purchased from Thermo Fisher or Sigma-Aldrich and used as received. Small molecules (R848, LCL-161 and ruxolitinib) were purchased from MedChem Express and used as is. Milli-Q water was obtained from a water filtration system.

### Synthesis and characterization of the CANDI wafer

#### Synthesis of CANDI wafer material

The synthesis of the CANDI wafer material was developed from a previously reported method for the synthesis of CANDI nanoparticles^28^. bsCD (275 mg, 1.0 equiv. to carboxylate) was dissolved in 2-(*N*-morpholino)ethanesulfonic acid (MES) buffer (3 ml, 50 mM, pH 5) and activated with *N*-(3-(dimethylamino)propyl)-*N*′-ethyl carbodiimide hydrochloride (EDC; Thermo Fisher, 1.2 g, 10.0 equiv. to carboxylate) and *N*-hydroxysuccinimide (NHS; Sigma-Aldrich, 228.5 mg, 5.0 equiv. to carboxylate) for 10 min at 25 °C. A solution containing l-lysine (Sigma-Aldrich, 200 mg, 0.5 equiv. to carboxylate) in MES buffer (0.35 ml) was added rapidly under vigorous stirring, and the reaction was allowed to stir for 24 h at 25 °C. The resulting viscous gel was added dropwise to ice-cold absolute ethanol solution (50 ml), then centrifuged for 5 min at 800 r.c.f. to yield a white precipitate, which was decanted and dissolved in water (6 ml). The opaque and viscous gel was then dialysed for 48 h in water and consecutively lyophilized for 48 h to yield the unloaded wafer material in powder form, which was characterized by dynamic light scattering (2 mg ml^−1^, 1× PBS) and zeta potential (2 mg ml^−1^, 0.1× PBS) and stored at −20 °C. An overview of the wafer materials used in the study is provided in Supplementary Table 3.

#### Small-molecule loading and wafer preparation

R848A (0.04 mg), LCL-161 (0.1 mg) and ruxolitinib (0.08 mg) were dissolved in dimethylsulfoxide (DMSO, 5 µl), and a solution of CANDI wafer material (10 mg) in water (95 µl) was used for payload loading to a final DMSO concentration of 5%. After vigorous vortexing, the resulting small-molecule-loaded CANDI wafer material was lyophilized for 48 h to yield a white powder, which was subsequently pressed into a wafer shape.

#### Fluorescent labelling of the CANDI wafer

Lyophilized CANDI wafer material (20 mg) was dissolved in water (1 ml), then AF647 succinimidyl ester (Thermo Fisher; 2 mg ml^−1^ in DMSO) was added. The reaction was incubated for 2 h at 37 °C in a thermocycler (Eppendorf, 550 r.p.m.). The resulting fluorescent wafer material particles were purified by dialysing for 48 h in water, then lyophilized for 48 h before being pressed into the wafer shape.

#### Drug-release kinetics

The kinetics of drug release were investigated using a closed dialysis system with a 3-kDa-molecular-mass-cutoff membrane (Pur-A-Lyzer Midi Dialysis Kit). CANDI wafers (60 mg) loaded with R848 (0.24 mg), LCL-161 (0.6 mg) and ruxolitinib (0.48 mg) were placed in PBS (1×, 1 ml) and dialysed against PBS (1×, 5 ml) at 23 °C under continuous shaking. The percentage of eluted molecules was quantified by analysing liquid chromatographs at specified time points (*t* = 0, 0.33, 0.5, 1, 2, 5, 8, 24, 55, 76, 98, 122 and 144 h). For analysis, 90-μl aliquots were injected into a liquid chromatography mass spectrometer, and the system was replenished with an additional 90 μl of PBS after each injection. Each payload (R848, ruxolitinib and LCL-161) was identified by its distinctive retention time (R848 = 0.78 min, ruxolitinib = 0.95 min and LCL-161 = 1.01 min) and mass-to-charge ratio (*ES*−: R848 = 313, ruxolitinib = 305 and LCL-161 = 499). The cumulative drug release was determined by calculating the ratio of the integrated area under the curve for each eluted peak to the total area under the curve of chromatographs obtained from non-membrane controls. All experiments were conducted in distinct triplicates (*N* = 3) to ensure the reproducibility and reliability of the results.

#### CANDI wafer–Gd conjugate

CANDI wafer (100 mg) was dissolved in dichloromethane (DCM, 5 ml) and activated using EDC (60 mg, 386.5 μmol) and NHS (79 mg, 686.3 μmol) overnight. The resulting solution was precipitated using diethyl ether. The activated CANDI was then dissolved in a mixture of DCM (5 ml) and DMSO (3 ml), followed by the addition of Gd-DO3A-butylamine (24 mg, 36 μmol) for 24 h. Subsequently, the conjugated CANDI-Gd was re-precipitated using diethyl ether and subjected to lyophilization. The resulting product was dissolved in acetonitrile (CAN), and unreacted Gd-DO3A-butylamine was removed by filtration using a 3.5-k Amicon filter, followed by lyophilization. MRI with implanted Gd wafers was conducted in triplicates (*N* = 3 mice).

### SEM

The surface morphology of the CANDI wafer was examined by field-emission SEM (FE-SEM; Zeiss Gemini 360). The secondary electron (SE2) detector obtained images with a 10-keV electron beam. Elemental analysis was performed using energy-dispersive X-ray spectroscopy.

### Cell models

The primary glioma cell line model for this study was CT-2A, a syngeneic GBM model histologically similar to human glioblastoma^52^. Cells were obtained from S. Rabkin (CT-2A), X. Breakefield (CT-2A-mCherry-luc) and K. Yang (CT-2A-H2B-mApple). We used CT-2A mCherry-luc cells for pharmacokinetics experiments, MRI experiments, bioluminescence imaging and survival studies. For in vitro maturation (IVM) and histology experiments, we used CT-2A H2B-mApple. In separate control experiments, we determined that all CT-2A subclones had similar growth rates in vitro and in vivo. The second GBM cell line was SB-28, obtained from H. Okada^67^. In this model, there is invasive GBM into normal brain parenchyma, and immunofluorescence shows a heterogeneous but never abundant T cell infiltration^67^. Murine CT-2A and SB-28 cells were cultured in DMEM (Corning) containing 4.5 g l^−1^ glucose and 10% FCS. Cells were detached from plastic with accutase (Sigma-Aldrich).

Immortalized mouse BMDMs (iMACs)^68^ were acquired from C. L. Evavold and used to assess toxicity (Fig. 3). iMAC cells were plated and grown in DMEM, supplemented with 10% fetal bovine serum (Corning) and 1% penicillin–streptomycin (Corning) at 37 °C. On reaching confluency, cells were split using 0.05% trypsin/0.53 mM ethylene diamine tetraacetic acid (EDTA; Corning), and all in vitro assays were performed after the cells reached 90% confluency. The cell lines were tested mycoplasma-negative.

Human THP-1 cells (TIB-202, ATCC), a cell line isolated from the peripheral blood of an individual with acute monocytic leukaemia, were used for efficacy testing. THP-1 cells were differentiated into macrophages and incubated with different amounts of wafer material.

An overview of the cell lines used in the study is provided in Supplementary Table 4.

### Toxicity

Cells were seeded in 96-well plates at a density of 8,000 cells per well and incubated for 24 h at 37 °C and 5% CO_2_ before use. Cells were incubated with CANDI wafer (50% DMSO in medium was used for the negative control, as well as medium only, without CANDI wafer, for the positive control) for 24 h in a total volume of 100 µl before 10 µl of alamarBlue cell viability reagent (Invitrogen, DAL1025) was added per well. Cells were incubated further for 2 h at 37 °C and 5% CO_2_, and fluorescence emission was read at a wavelength of 590 nm using an excitation wavelength of 550 nm (both with a bandwidth of 20 nm). The experiment was conducted in triplicates. To assess systemic toxicity, blood was collected from mice 12 days following CANDI treatment. Complete blood counts and clinical chemistry analyses were then performed.

### Mouse models

We used various mouse models to study the different aspects of drug delivery, IL-12 induction and therapeutic efficacy. All experiments were approved by the MGH Institutional Animal Care and Use Committee (IACUC) under protocol no. 2021N000135 and performed according to MGH IACUC regulations. The maximal tumour burden/size of 8 mm in diameter was not exceeded. A total of *N* = 145 mice were used (Supplementary Table 5). This included immunocompetent female C57BL/6J wild-type mice for GBM implantations (*N* = 141, C57BL/6J, strain no. 000664, JAX), female MerTK–GFP^69^ mice for co-localization IVM studies (*N* = 1; strain no. 036071, JAX) and female IL-12–eYFP mice for GBM implantations, IVM mechanistic studies and bone marrow harvesting (*N* = 3; B6.129-IL-12btm1.1Lky/J, strain no. 006412, JAX). Mice were assigned randomly to the different experimental groups.

### Tumour implantation

We used 10–12-week-old C57BL/6J mice, anaesthetized them with isoflurane, shaved their heads and immobilized the cranium in a stereotactic frame (Kopf). The surgical site was sterilized with two cycles of betadine–isopropanol. Using a Dremel tool with a burr (Fine Science Tools, 19007-07) trepanning was performed and 5 × 10^4^ CT-2A (2.5 × 10^4^ SB-28) cells diluted in 2-µl sterile PBS (Sigma-Aldrich) were stereotactically implanted into the right cerebral cortex (coordinates: 2 mm right lateral of the bregma and 2 mm posterior to the coronal suture with an injection depth of 0.7 mm below the dural surface) using a 10-µl Hamilton microsyringe driven by a fine-step stereotactic device (Kopf). CT-2A mCherry-luc cells were used for therapeutic efficacy studies, and CT-2A H2B-mApple cells were utilized for IVM studies and multiplexed FAST-profiling of GBM.

### Tumour resection and wafer implantation

On day 12 after tumour implantations, animals were immobilized in a stereotactic frame (Kopf), then the surgical site was sterilized with two cycles of betadine–isopropanol. A right paramedian rostral–caudal curvilinear incision was made, and subcutaneous layers of the scalp, including the periosteum, were removed. Under ×10–20 magnification, with a radius of 2 mm around the initial tumour injection site, burr holes were made using a Dremel tool with a burr (Fine Science Tools, 19007-07). The burr holes were connected using fine microscissors, and the bone flap was consecutively lifted off with fine forceps, resulting in a craniectomy. After peeling away the dura mater, the tumours were resected, leaving behind a residual tumour volume and creating a cavity for consecutive wafer implantation. The resection cavity was then left empty, or drug-loaded wafers were placed using fine forceps. The skin was closed with interrupted sutures.

### Histology

Frozen tissue sections were cut to 5-µm thickness and then processed for immunofluorescence. Frozen tissue sections were thawed, rehydrated and blocked with Intercept blocking buffer (LI-COR) for 30 min before antibody staining. The tissue sections were incubated with antibodies (Supplementary Table 6) for 1–2 h and washed with PBS for 10 min three times. An Olympus BX-63 microscope was used for image acquisition (Metamorph software version 7.10.4). CellProfiler version 4.1.8 was used for analysis of the tissue section images. A custom Python script was used to generate synthetic image maps.

### Intravital microscopy

#### Brain window tumour model

All experiments were approved by the MGH Institutional Animal Care and Use Committee (IACUC) and performed according to MGH IACUC regulations. Cranial windows were implanted according to established methods, with modifications^70^. The heads of 10–14-week-old mice were shaved, animals were immobilized in a stereotactic frame (Kopf), and the skull was sterilized with two cycles of betadine–isopropanol. A large oval skin area was removed from behind the ears to between the eyes, surrounding the lambda and bregma sutures. The periosteum was pushed to the side, and all tissue on top of the skull was scraped off. The rim of the 5-mm circular section of the head, excluding the lambda and bregma, was sanded down using a Dremel tool with a burr (Fine Science Tools) and removed to provide an opening to the brain. Using stereotaxic positioning, 2 µl of OptiMEM (Thermo Fisher Scientific) with 10^5^ CT-2A-H2B-mApple cells were injected at ~1-mm depth near the middle of the opening, avoiding vasculature. Gelfoam and saline were used to remove blood during surgery and after injection. A drop of saline and an 8-mm round cover glass were placed onto the opening. Super glue was used to attach only the rim of the cover glass to the skull, avoiding any contact between the adhesive and the brain. Dental cement was used to cement the cover glass onto the skull, cover skull areas without skin, and form an elevated rim for water immersion imaging.

#### Confocal imaging

All confocal images were collected using a customized Olympus FV1000 confocal microscope (Olympus America). A ×2 (XLFluor, NA 0.14), a ×4 (UPlanSApo, NA 0.16) and an XLUMPlanFL N ×20 (NA 1.0) water immersion objective were used for imaging (Olympus America). CT-2A H2B-apple tumour cells, CANDI^AF647^ and vascular probes were excited sequentially using 405-nm, 473-nm, 559-nm and 633-nm diode lasers, respectively, in combination with a DM-405/488/559/635-nm dichroic beamsplitter. Emitted light was further separated by beamsplitters (SDM-473, SDM-560 and SDM-640) and emission filters (BA430-455, BA490-540, BA575-620 and BA655-755) (Olympus America). Confocal laser power settings were carefully optimized to avoid photobleaching, phototoxicity or damage to the brain. All images were processed using Fiji (ImageJ2, version 2.3/1.53f).

### Flow cytometry

Tissues were isolated, mechanically dissociated using surgical scissors, and digested using collagenase IV at 0.2 mg ml^−1^ in RPMI 1640 at 37 °C for 45 min with vigorous shaking. After digestion, tissues were filtered through a 40-µm cell strainer and resuspended in protein-free PBS. The cells were stained using AquaAmine Live Dead Fixable viability stain (Thermo Fisher) and then washed with PBS. They were then resuspended in FACS buffer (PBS with 2 mM EDTA and 2% FCS) and stained with Fc block (Biolegend) and fluorochrome-conjugated antibodies. Sample data were acquired using an Attune NxT flow cytometer (Thermo Fisher) with the Attune Cytometric software (version 5.3.0), and data were analysed using FlowJo 10.9.0 software (TreeStar).

### Bioinformatic analysis of published scRNA-seq datasets

Mouse data^30^ were downloaded from Zenodo^71^. Human GBM Full aggregate data from newly diagnosed patients^31^ were downloaded from the Brain Immune Atlas (www.brainimmuneatlas.org/download.php). Seurat (v4)^72,73^ was used for scRNA-seq data analysis. We constructed a Seurat object using the feature–barcode matrix for each sample. A series of quality filters were applied to the data to remove low-quality cell barcodes: possible debris with too few genes expressed (<300); possibly more than one cell with too many genes expressed (>6,000–10,000 according to the sample); possible dead cell or a sign of cellular stress and apoptosis with too high proportion of mitochondrial gene expression over the total transcript counts (>20%). Each sample was scaled and normalized using Seurat’s ‘NormalizeData’ and ‘ScaleData’ functions. We then merged all samples and repeated the same scaling and normalization method. For human data, all cells in the merged Seurat object were integrated using ‘IntegrateData’ and the top 30 principal component analysis dimensions. Data were clustered using Seurat’s ‘FindNeighbors’ and ‘FindClusters’ (with the parameter resolution = 0.5) functions. The resulting merged and normalized matrix was used for the subsequent analysis. Differentially expressed genes (DEGs) were identified by the ‘FindMarkers’ function, comparing cells belonging to one subtype to the rest. The Wilcoxon statistical test was used. log_2_FC > 0.25 and false discovery rate (FDR) < 0.05 were used to filter DEGs. Clusters were annotated as described in the original papers. Macrophage and microglia-specific markers were used for further characterization. Geneset enrichment analysis was performed with the ‘enricher’ function. The hallmark collection of the MSigDB repository was tested for this analysis with the following parameters: pAdjustMethod = ‘BH’, pvalueCutoff = 0.05, qvalueCutoff = 0.05.

### Bulk RNA-seq

To determine the effects of the wafer on phagocytic cells, bulk RNA-seq was performed. BMDMs were isolated and differentiated as previously described^27^. Cells were then stimulated for 24 h with drug-loaded wafer material to induce activation. RNA was isolated using the RNeasy Plus Mini Kit (Qiagen). Final RNA concentration was determined by absorbance (Nanodrop), and samples were stored at −80 °C until shipment for sequencing (NovoGene).

### Cytokine measurements

#### In vitro cytokine expression

To determine the relative expression levels of several cytokines and chemokines in response to the (drug-loaded) wafer, an immunoassay (R&D Proteome Profiler Mouse XL Cytokine Array) was performed with cell culture supernatants from BMDMs that had been stimulated for 24 h with empty or drug-loaded wafer material.

#### Peripheral IL-12 p70 levels

Drug-loaded wafers were implanted as described in the section Tumour resection and wafer implantation. Blood draws were performed on days 1, 2, 4, 8 and 14 after implantation. Whole blood (100–200 µl) was collected in K2EDTA-coated blood collection tubes (BD Microtainer 365967) and spun down at 2,000 r.c.f. for 10 min. Serum was stored at −20 °C for downstream analysis. IL-12 p70 levels were measured using a mouse IL-12 p70 quantikine ELISA kit (R&D Systems M1270). The experiment was conducted with three animals per group (*N* = 6; one animal in the CANDI wafer-treated cohort had to be euthanized on day 10 due to poor body condition from the repeated blood draws).

### MRI

MRI of GBM-bearing mice was performed for different reasons: (1) to monitor the growth of GBM following implantation; (2) to monitor the evolution following GBM resection and wafer implantation (at baseline (day 13) and after treatment (day 22)); (3) to image the dissolution of wafer material. All imaging was performed on an animal 4.7-T MR imaging unit (Bruker Pharmascan) under respiration-monitored isoflurane anaesthesia. Coronal imaging parameters for pre- and post-contrast enhanced T1-weighted imaging were as follows: repetition time (TR) = 700 ms, echo time (TE) = 14 ms, matrix size 256 × 256 and slice thickness 0.5 mm. Twelve sections were acquired. Imaging parameters for pre-enhanced T2-weighted imaging were as follows: TR = 4,000 ms, TE = 53.3 ms, matrix size 256 × 256, slice thickness 0.5 mm. Twelve sections were acquired. Tumour or wafer volumes were calculated by using the Horos image-processing software for tumour volumetric data via region of interest-based three-dimensional (3D) analysis of Gd-DTPA enhanced T1-weighted MR images (Horos, horosproject.org).

### Bioluminescence imaging

Bioluminescence imaging was performed using an Ami HTX Spectral Instruments Imaging instrument at baseline (day 12) and after treatment (days 19 and 26). Mice received an intraperitoneal injection of luciferin (5 mg per mouse) and were maintained under respiration-monitored isoflurane anaesthesia during imaging. Imaging parameters were as follows: 10-s and 0.5-s exposure; binning levels 1, 2, 4 and 8. Total flux (photons s^−1^) was used for signal quantification.

### Testing in human organotypic tissue slices

To determine the effects of wafer material on human GBM tissue, we studied freshly resected GBM sections. This study was approved by the Human Research Ethics Committee from the Geneva University in Switzerland (CCER 2022-02109 and 2023-01928). Patients included a 37-year-old male and a 52-year-old male. Informed consent was obtained from all patients who had been enroled (*n* = 2). Patients did not receive compensation. Freshly resected GBM specimens were transferred from the operating room to the laboratory in a 50-ml conical tube containing ice-cold medium (high-glucose DMEM supplemented with HEPES 1× and 1× penicillin–streptomycin solution). Pieces were punched from fresh tumour, embedded into 2% agarose, then sliced on a vibratome to a thickness of 400 µm. GBM slices were cultured on a polytetrafluoroethylene insert (pore size 0.4 µm) in 24-well plates with culture medium (neurobasal medium supplemented with B27 1×, d-glucose 1.5 mg ml^−1^, Glutamax 600 µM, l-glutamine 400 µM and 1× penicillin–streptomycin solution). Tumour slices (*n* = 5 per condition) were cultured for 34 days, and the CANDI wafer process was performed on the second day. CANDI wafers (0.01 mg ml^−1^, 0.1 mg ml^−1^ or 0.5 mg ml^−1^) or not were added to the GBM slices for 72 h in an incubation system (37 °C, 5% CO_2_). Supernatant was collected after 4, 24, 48 and 72 h for IL-12 p70 assay. GBM slices (*n* = 3 per condition) were then fixed in paraformaldehyde 4% for immunohistofluorescence. The experiments yielded comparable results between the two patients, supporting the robustness and reproducibility of the findings.

### Statistics and reproducibility

All statistical data analyses were performed using GraphPad Prism 10.0.2 software or R (version 4.3.0, version 4.4.0) and the results were expressed as mean ± s.d. or with 95% confidence intervals (CIs). For normally distributed datasets, we used a two-tailed Student’s *t*-test and one-way ANOVA followed by Bonferroni’s multiple comparison test. When variables were not normally distributed, we performed non-parametric Mann–Whitney or Kuskal–Wallis tests. Survival data were analysed using the Kaplan–Meier method and two-sided log-rank (Mantel–Cox) test to analyse the statistical significance of the difference between survival groups. (Restricted) mean survival times and hazard ratios with 95% CIs were calculated. Survival studies were repeated in independent experiments, yielding consistent results, and data were pooled for statistical analysis. *P* values > 0.05 were considered not significant (NS), *P* values < 0.05 were considered significant. Experimental numbers are listed in the figure legends, and in Supplementary Table 5. No statistical methods were used to predetermine sample sizes. Animals and samples were randomly assigned to the various experimental groups. Data collection and analysis were not performed blind to the conditions of the experiments. If not stated otherwise, no animals or samples were excluded. The number and type of replicates (biological or technical) are indicated in each figure legend.

### Reporting summary

Further information on research design is available in the Nature Portfolio Reporting Summary linked to this Article.

## Supplementary information

Supplementary Information Supplementary Figs. 1–23

Reporting Summary

Peer Review File

Supplementary Data 1 Supplementary Source Data

Supplementary Tables 1–6 Supplementary Table 1: Blood counts and clinical chemistry of CANDI-treated mice. Supplementary Table 2: Summary of statistical survival analyses. Supplementary Table 3: Overview of wafer materials used in the study. Supplementary Table 4: Cell lines. Supplementary Table 5: Mouse strains and experimental numbers. Supplementary Table 6: List of antibodies used.

Supplementary Movie 1 Synthesis of wafer material. The crosslinked cyclodextrin material was first prepared as a high molecular weight gel material, which was subsequently lyophilized for implantation.

Supplementary Movie 2 Internalization of wafer material into BMDM cells. Cells were stained with a green membrane dye (CellBrite) and nuclei were stained with DAPI (blue). The CANDI wafer material was labelled with AF647 (red). Note the uptake of wafer material into cells, presumably the phagosome/lysosomal complex.

Supplementary Movie 3 In vivo appearance of wafer material in resection cavity. Intravital imaging was performed three days after CT-2A resection in a merTK-GFP mouse and wafer implantation (wafer labelled with AF647, red). In this model, the TAMs appear green and can be seen in large numbers adjacent to the implanted wafer. Some of the TAMs closest to the wafer seem to have internalized the material. Thirty-minute movie.

## Source data

Source Data Figs. 1-7 Statistical source data

## Data Availability

The data supporting the results in this study are available within the paper and the Supplementary Information or are available from the corresponding author upon reasonable request. The statistical source data and the data presented in the graphs within the figures are available as source data. The publicly available single-cell RNA-sequencing datasets used in the bioinformatic analyses can be accessed in the Gene Expression Omnibus (GEO) under accession code GSE197879 and in the European Genome-phenome Archive (EGA) under accession code EGAD00001006778. The acquired RNA-seq data are available from https://csb.mgh.harvard.edu/bme_software. Source data are provided with this paper.

## Code availability

The custom Python script used for analysis of the IHC data is available from https://csb.mgh.harvard.edu/bme_software.
