# Supplementary material for: Targeting immunosuppressive myeloid cells via implant-mediated slow release of small molecules to prevent glioblastoma recurrence
Source: Nat Biomed Eng. 2025 Oct 22;10(7):1431–44. doi: 10.1038/s41551-025-01533-2 (PMC13150848; doi:10.1038/s41551-025-01533-2)

nature biomedical engineering

Supplementary information

https://doi.org/10.1038/s41551-025-01533-2

Targeting immunosuppressive myeloid cells via implant-mediated slow release of small molecules to prevent glioblastoma recurrence

In the format provided by the authors and unedited

Fig. S1: Comparison of the TME in CT-2A (A-C) and human glioblastoma (D-F). A. Umap of major immune cell types in CT-2A originally obtained by[30]. Note the prominent myeloid cell population comprising primarily macrophages and monocytes. The microglia and lymphoid populations are small. **B.** Violin plots show expression of Trem2 and Spp1 genes in different clusters. SPP1, in particular, has recently been identified as an immunosuppressive biomarker[35]. **C.** Gene set enrichment analysis for the Monocyte, TAM and Microglia clusters. Hallmark pathways invariably involve NFkB and hypoxia. Statistical significance was calculated using a hypergeometric test (Fisher's exact test) with p-values adjusted for multiple testing using the Benjamini–Hochberg method. **D.** Umap of major immune cell types in human GBM originally obtained by[31]. The myeloid compartment is still sizable but the microglial compartment is larger than in the mouse. **E.** SPP1 and TREM2 levels across human cell types. Note higher SPP1 levels than in the mouse. **F.** Gene set enrichment analysis in human glioblastoma. Hallmark pathways upregulated include complement, inflammatory response, and NFkB signaling. Statistical significance was calculated using a hypergeometric test (Fisher's exact test) with p-values adjusted for multiple testing using the Benjamini–Hochberg method.

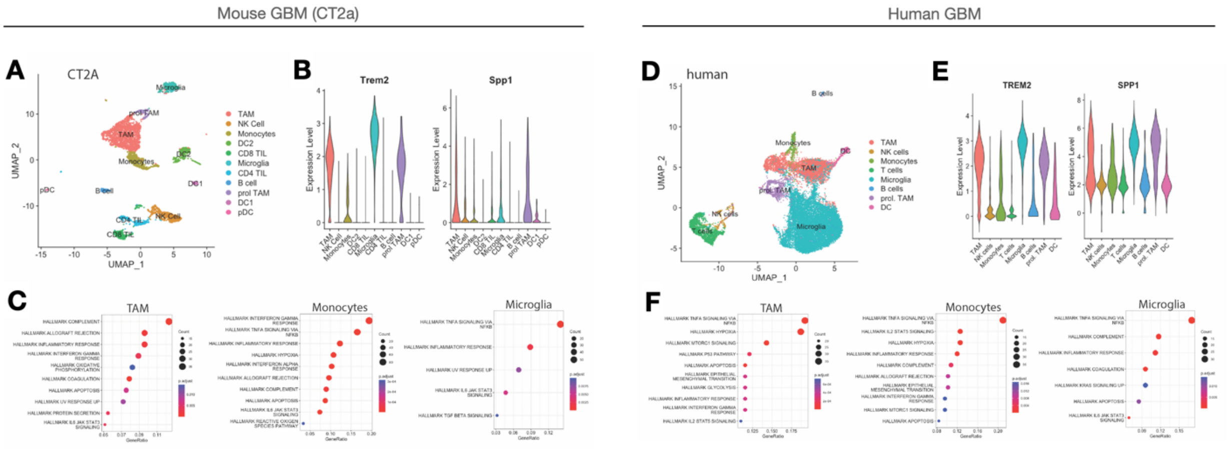

Fig. S2: The CANDI wafer material consists of cross-linked cyclodextrins CD that serve as a supramolecular guest-host structure to deliver small molecules to TAM. Carbohydrate-containing structures have a naturally high affinity for TAM, and small molecule delivery to them can depolarize them to produce IL-12 to jumpstart anti-tumor effects. Created in BioRender. Weissleder, R. (2025), https://BioRender.com/4dpd1c6.

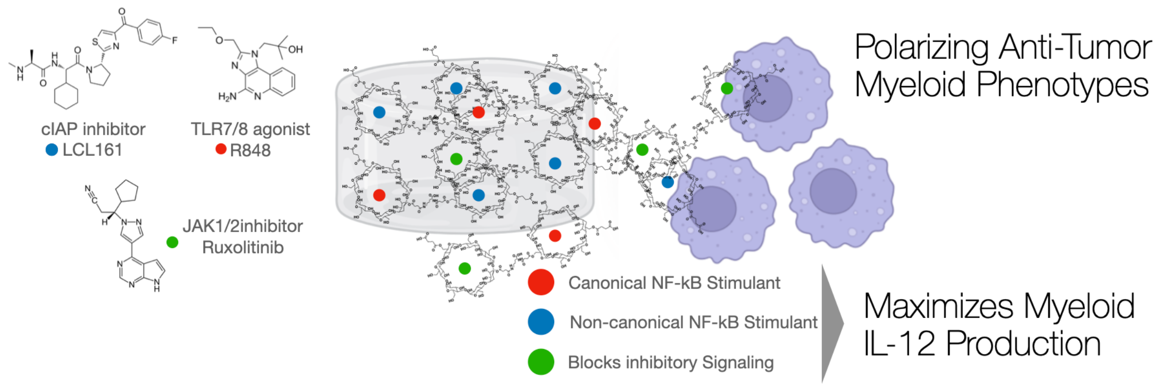

Fig. S3: Scanning electron microscopy (SEM) of the wafer surface. Representative SEM images of drug-loaded wafer at different magnifications. Note the porous surface. Multiple regions of the sample were imaged, and similar morphology was observed throughout. **A.** Scale bar 100 µm. **B.** Scale bar 20 µm. **C.** Scale bar 10 µm.

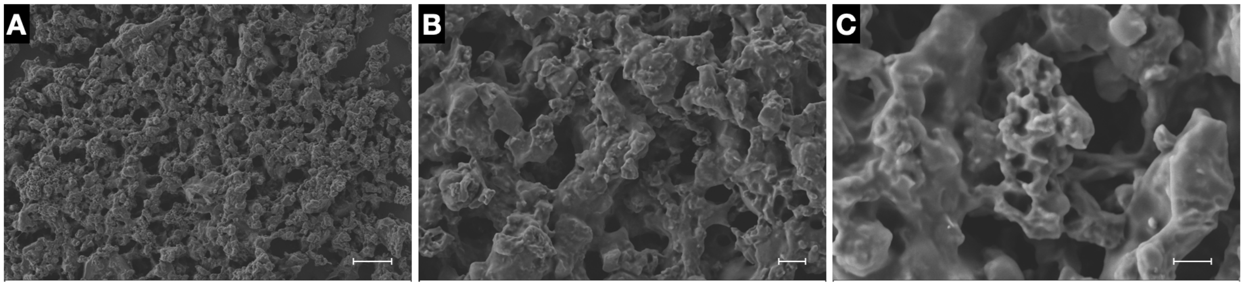

Fig. S4: EDX of the drug-loaded wafer. A. Scanning electron micrograph (SEM) of the drug-loaded wafer. Scale bar 10 µm. Multiple regions of the sample were imaged, and similar morphology was observed throughout. **B.** EDX spectrum showing the main components (C, O, N). The S and Cl are contributions from PBS. The Pt and Pd are from the staining procedure.

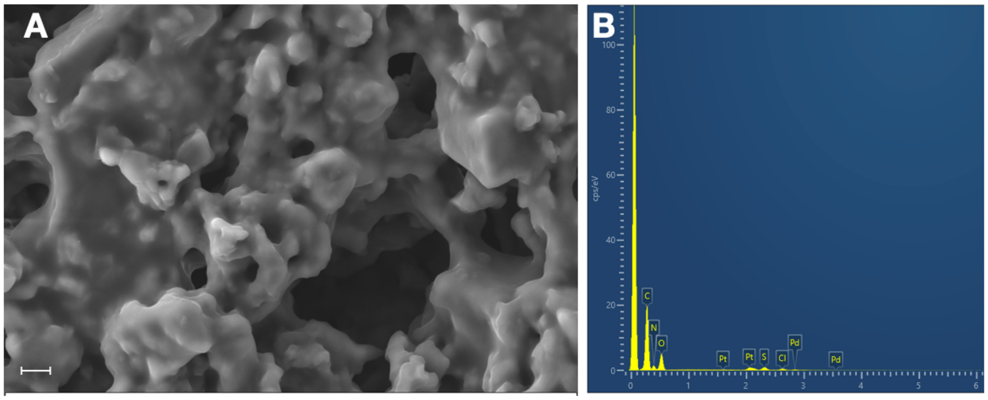

Drug loaded CANDI wafer	
Element	Wt%
C	58.1
O	24.7
N	11.1
S	3.0
Cl	1.4
Pd	1.4
Pt	0.3
Total:	100.00

Fig. S5: Drug release from wafer. A. Images of the wafers used for the dialysis experiments over 7 days. **B.** Release of payload in the closed dialysis system. At 144 hours, the material was still intact, but small molecule release had reached a maximum. The $T_{1/2}$ was approximately 45 hours, and the K_{off} rate was 0.01-0.02 in this system. At 144 hours, 8 % (range: 3.7-13.2%) of the drug remained in the wafer material, as determined by trypsinization experiments. n = 3 individual replicates.

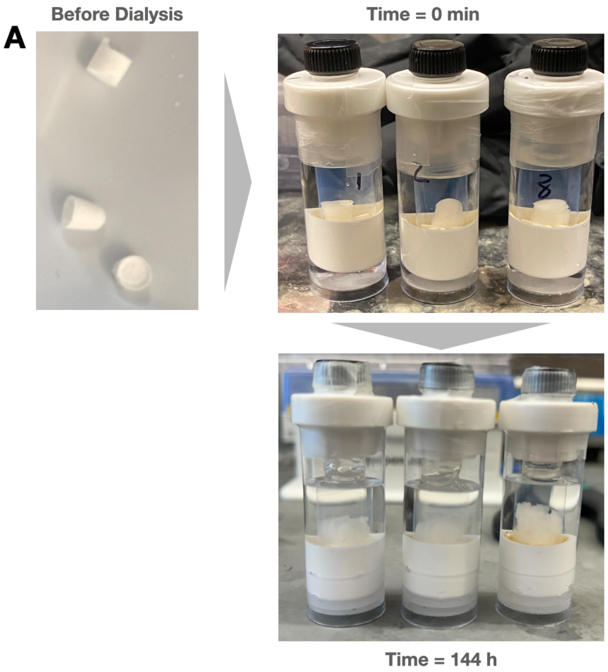

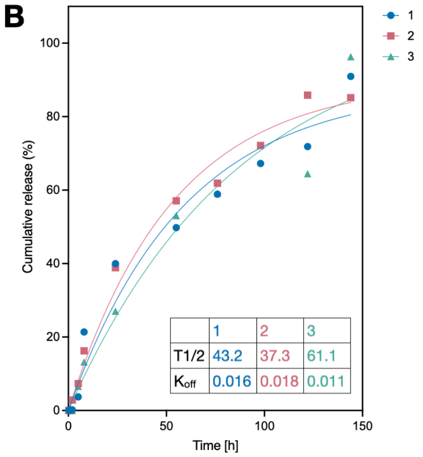

Wafer	Released at 144 h (%)	Remaining in wafer at 144 h (%)	Total of theoretical
1	90.9%	13.2%	104.1%
2	85.2%	10.8%	96%
3	96.3%	3.7%	100%

Fig. S6: In vivo degradation of wafer material. We created a covalently Gd-DOTA labeled wafer material (**A**) and implanted them in mice (**B**) following GBM resection. The wafer material appears markedly hyperintense and slowly degrades over time. In this example, and using an 6 mm^3 wafer, degradation was complete by day 14. Graphics created in BioRender. Weissleder, R. (2025), https://BioRender.com/4dpd1c6.

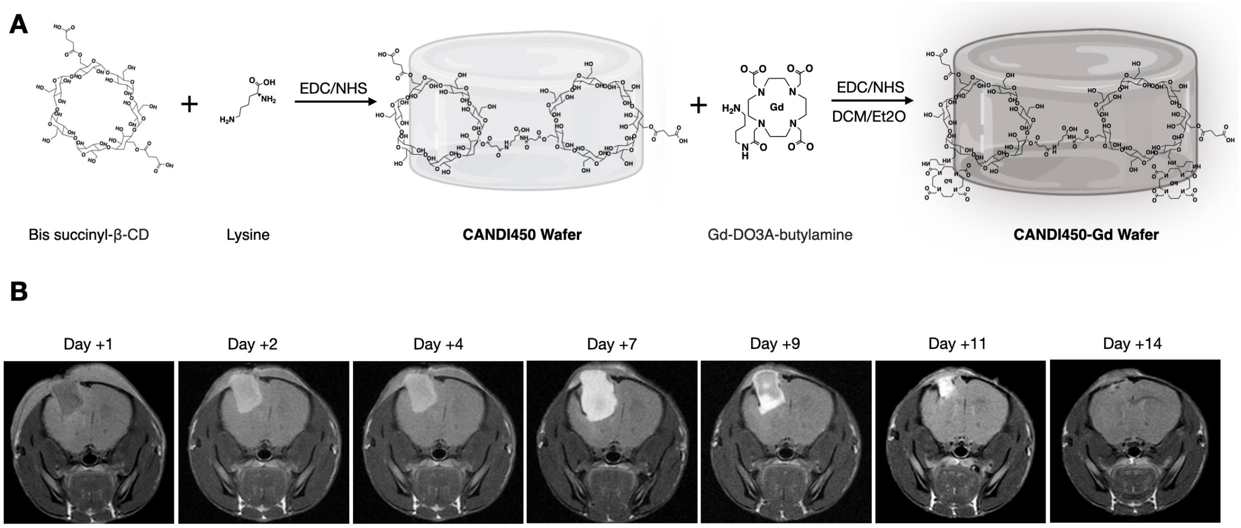

Fig. S7: Confocal Imaging reveals cellular uptake and lysosomal escape. Representative confocal microscopy image of macrophages (iMacs) treated with AF647-stained CANDI (white)/ Left: 24hr; right: 48 hr. Note the localization of CANDI in punctuate intracellular structures within 24 hrs and the following lysosomal escape (48 hrs) evidenced by cytoplasmic localization of the fluorescent compound best seen in the inserts. Cells are outlined for easier visibility. N=nucleus. Representative images are from one of three technical replicates with similar results.

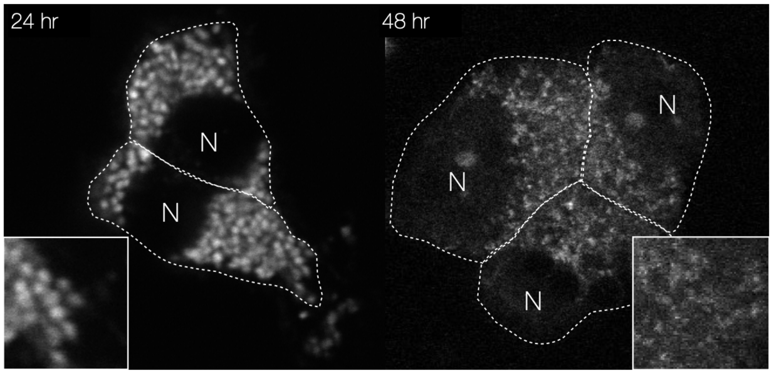

Fig. S8: Mechanism of cellular CANDI uptake. iMAC cells were incubated with CANDI-AF647 and different uptake inhibitors, and cellular uptake of CANDI-AF647 was assessed using flow cytometry. **A.** Gating strategy. **B.** Chlorpromazine inhibits clathrin-mediated endocytosis (Rho-GTPase). Wortmannin inhibits micropinocytosis/phagocytosis (PI3K). Imipramine inhibits macropinocytosis. EIPA inhibits macropinocytosis via Na/H exchange. The biggest effects were observed with chlorpromazine. n = 3 technical replicates, data are presented as mean values ± SD.

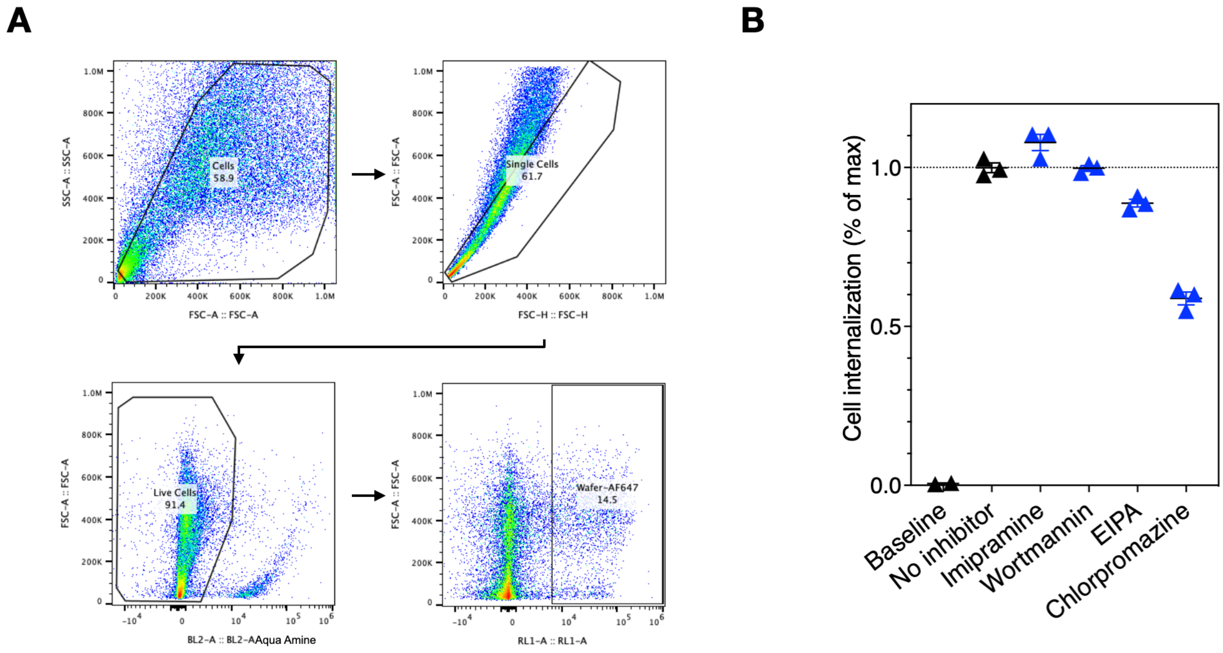

Fig. S9: RNAseq of BMDM exposed to different wafer materials. A. Comparison of empty wafer material against PBS. Note the lack of significantly upregulated or downregulated macrophage genes. **B.** Comparison of drug-loaded vs empty wafer material shows a myriad of upregulated and downregulated genes. Wald test with Benjamini-Hochberg FDR correction.

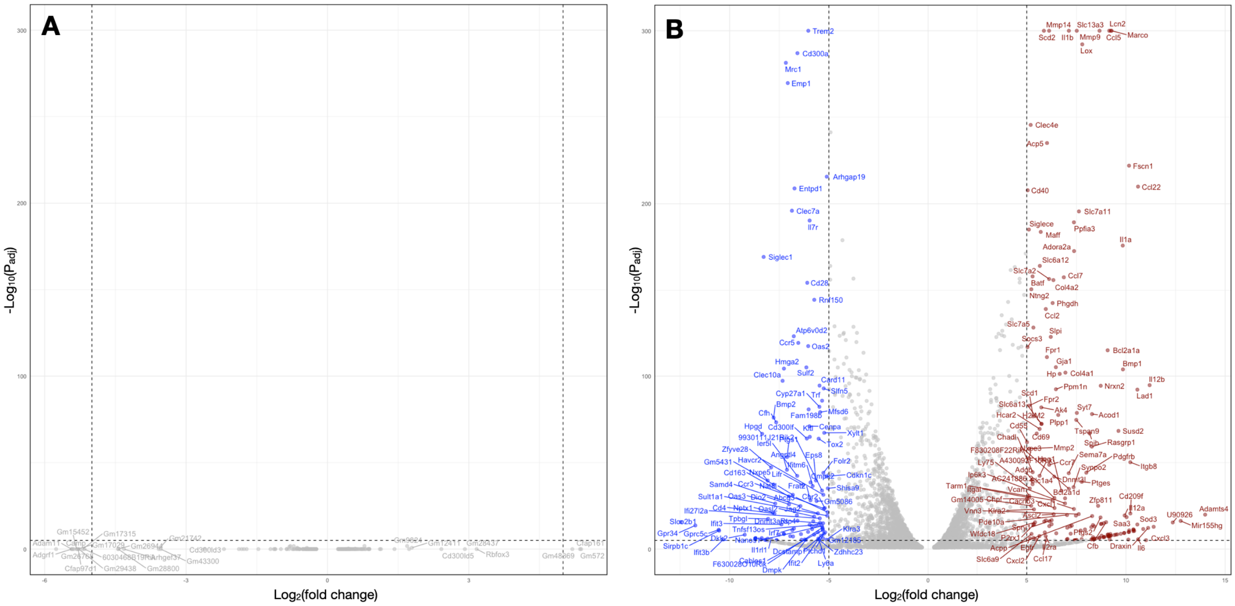

Fig. S10: GBM implantation and surgical resection. A. We chose the following coordinates for the intracranial implantation of 10^5 CT-2A (or SB-28) tumor cells: AP: -2mm; ML: -2mm; DV: -0.8mm. This allowed for tumor growth in the left hemisphere and surgical resection 12 days later. Graphics created with BioRender. Created in BioRender. Weissleder, R. (2025), https://BioRender.com/4dpd1c6. **B**. Six burr holes were created using a motorized drill. **C**. Craniotomy using fine scissors. **D** View of superficial CT-2A tumor. E Resection and debulking of the primary tumor. **F.** Implantation of the wafer. **G**. Wafer before implantation (diameter 2mm).

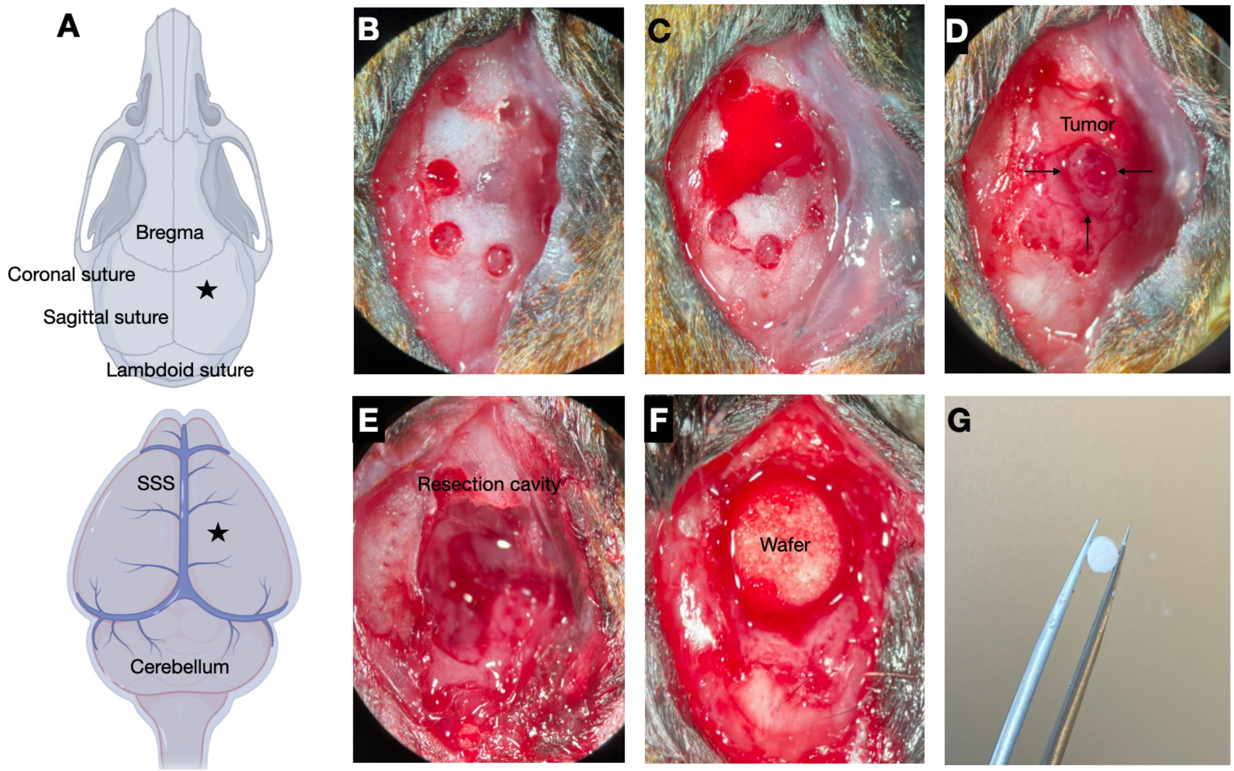

Fig. S11: In vivo degradation of the wafer. A. Intravital imaging was performed using a cemented brain window to serially image the GBM tumor microenvironment. We used a mer-TK-GFP mouse to natively image macrophages (green), and implanted AF647 labeled wafer (red). Note that the wafer material is fluorescent in the red and green channels (autofluorescence). Created in BioRender. Weissleder, R. (2025), https://BioRender.com/4dpd1c6. **B** High-resolution imaging 3 days after implantation of a wafer clearly shows cellular internalization of wafer material into macrophages surrounding the disk. Scale bar 20 µm. **C.** Serial imaging of a similar brain location over 4 days after wafer implantation into the brain (scale bar: 50 µm). Note the recruitment of green macrophages by day three and cellular uptake of digested wafer material (white arrows). **A**-**C.** Serial imaging was performed in one mouse over 4 days and multiple fields of view were imaged, yielding similar results. **B-C.** For colorblind-accessible versions of the images please see **Fig. S23**.

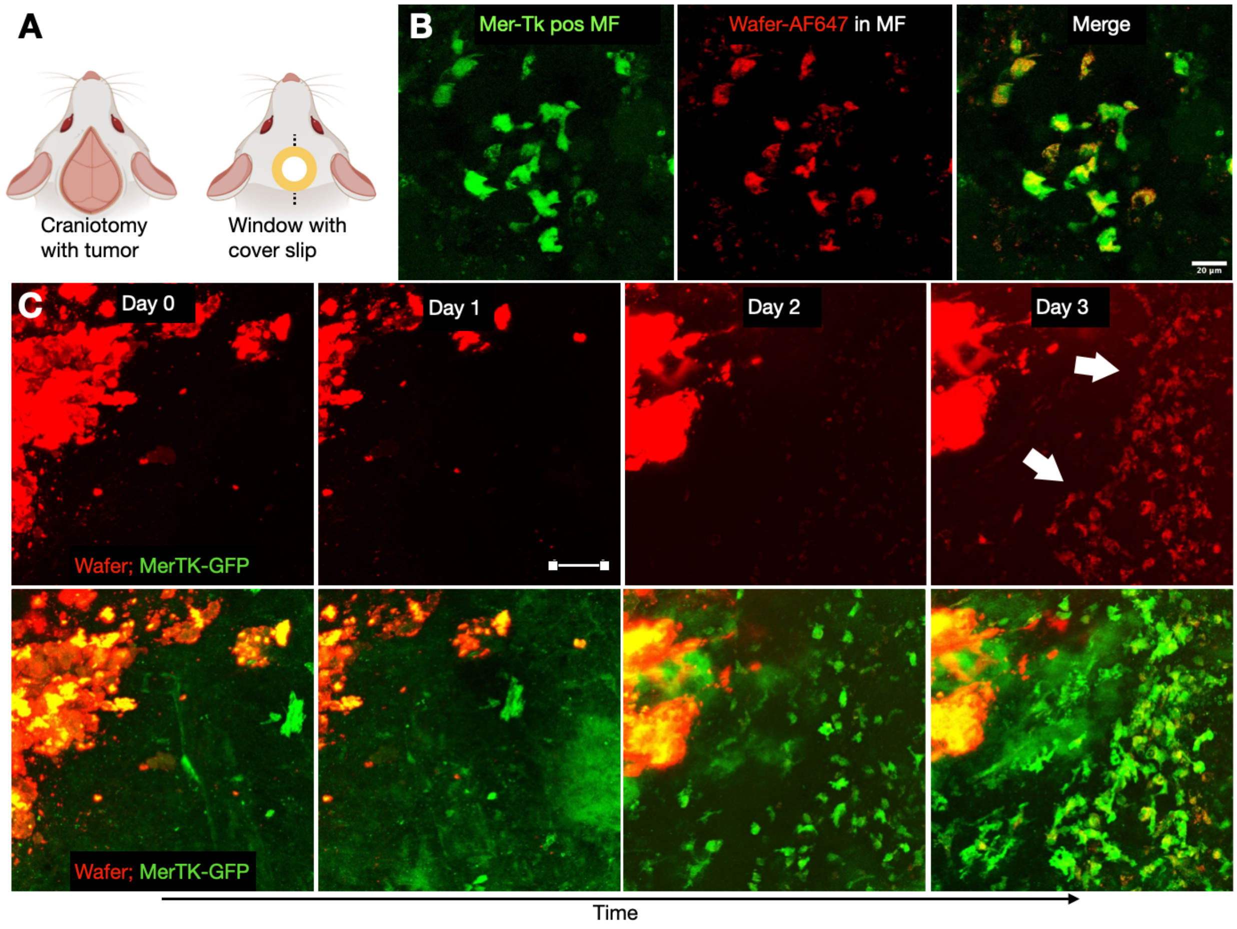

Fig. S12: MR imaging of survivors. MR imaging of four representative surviving mice 48, 64, and 96 days after tumor implantation of the primary GBM. Most animals showed a post-surgical fluid-filled resection cavity without any evidence of residual tumor by HE staining.

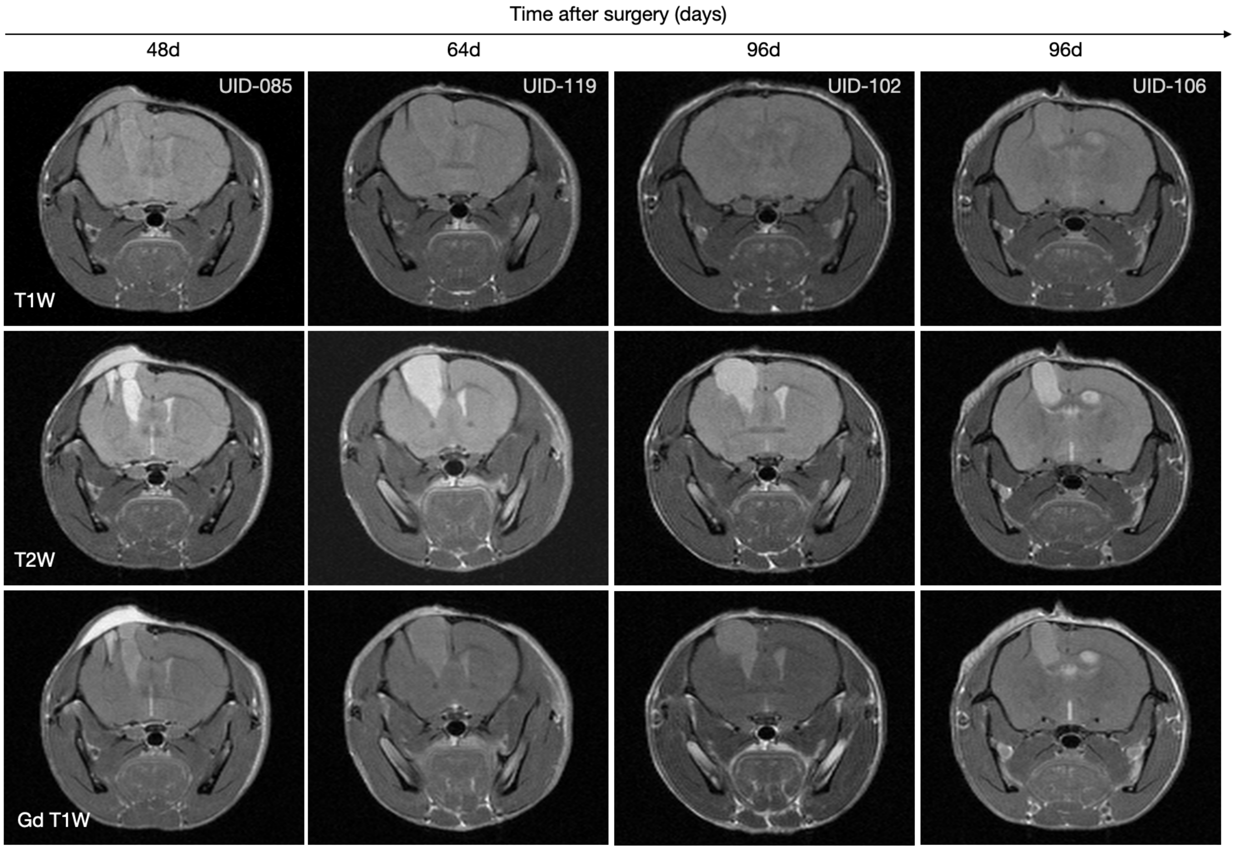

Fig. S13: Bioluminescence data on CT2A cohorts. All tumors were surgically resected at 12 days after implantation. In wafer-treated animals (n = 9 mice), the bioluminescence signal was much lower at 2 weeks after resection, with four out of nine animals back at baseline. Also, note the high mortality rate in the surgery-only group (n = 10 mice).

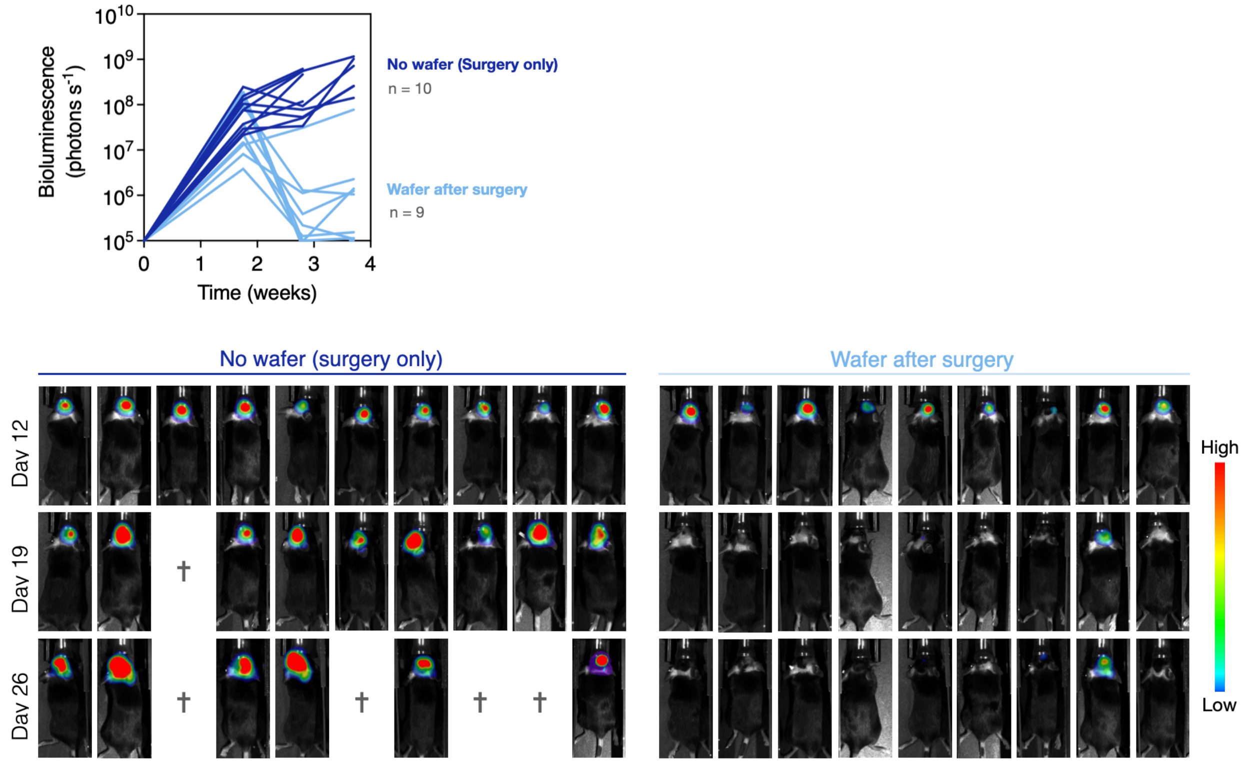

Fig. S14: Long-term effects. MR imaging and autopsy were performed on a representative mouse 96 days after the original tumor implantation. **A.** T1W MRI shows the resection cavity as isointense compared to the surrounding brain. **B**. Following intravenous administration of gadolinium. **C.** T2 weighted image shows the resection cavity to be hyper-intense. **D** and **E**. At autopsy, the reception cavity is fluid-filled and has smooth margins. **F.** Corresponding HE stain does not show any residual GBM.

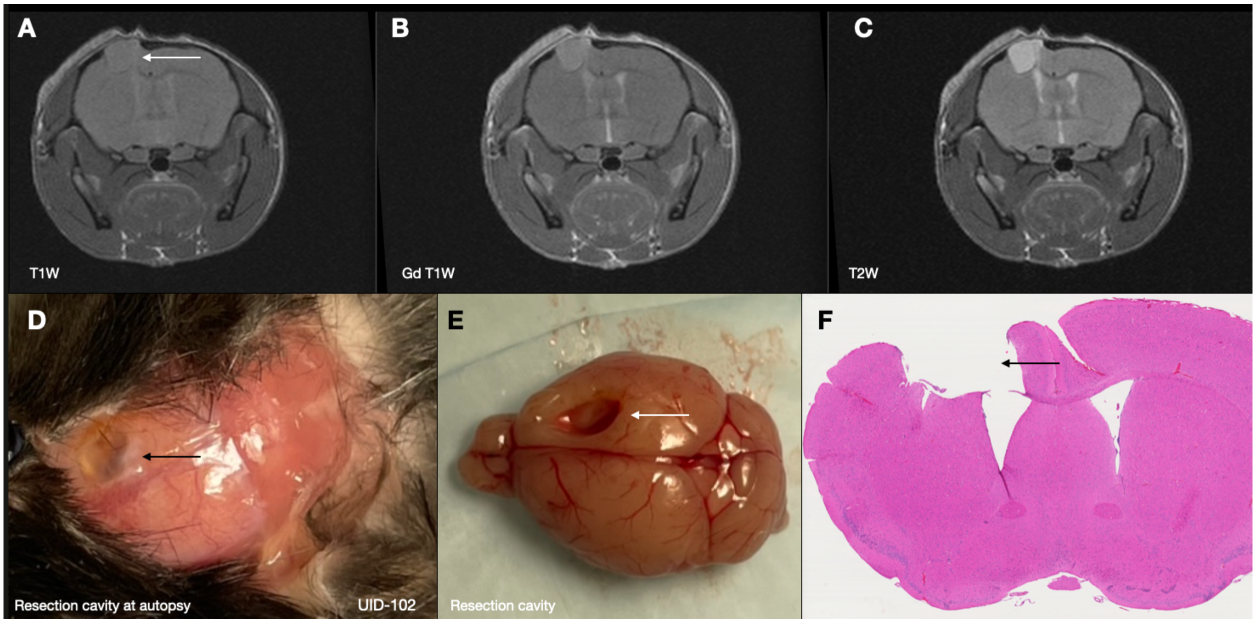

Fig. S15. Extended histologic studies in a surviving animal using H&E (left) or GFAP immunohistochemistry (right). Note the absence of glioblastoma around the cavity and also the absence of inflammatory response 90 days after resection. There is no apparent toxicity to neuronal structures.

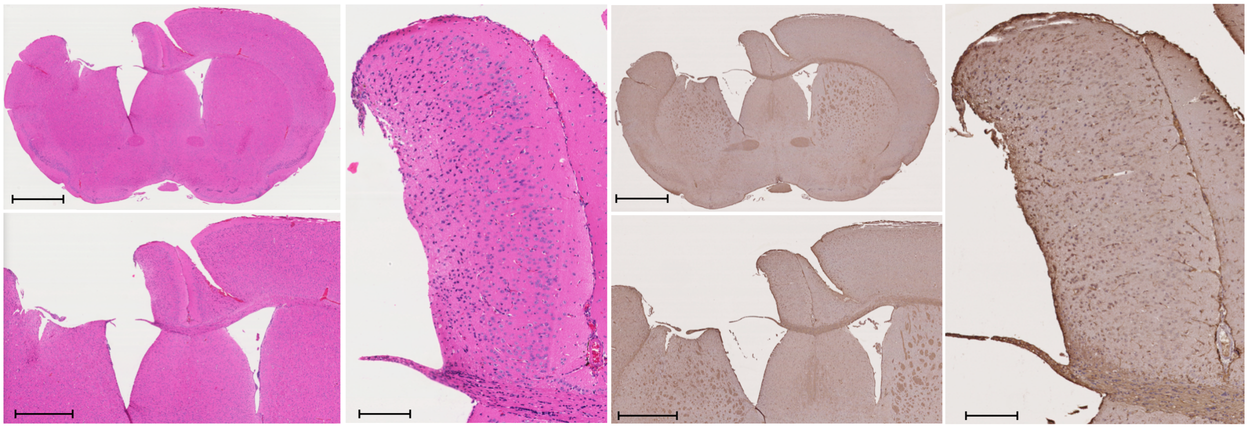

Fig. S16: Alternative GBM model. A. Overall experimental design for tumor implantation, resection with wafer implantation, and longitudinal monitoring. Here, we used the invasive murine SB-28 GBM model[67]. Created in BioRender. Weissleder, R. (2025), https://BioRender.com/4dpd1c6. **B**. Survival graphs showing differences between resection (dark blue, n = 10 mice) and resection with wafer implantation (light blue, n = 13 mice). Log-rank test: p=0.0009. For additional statistics, see **Table S2**. No other therapy was administered.

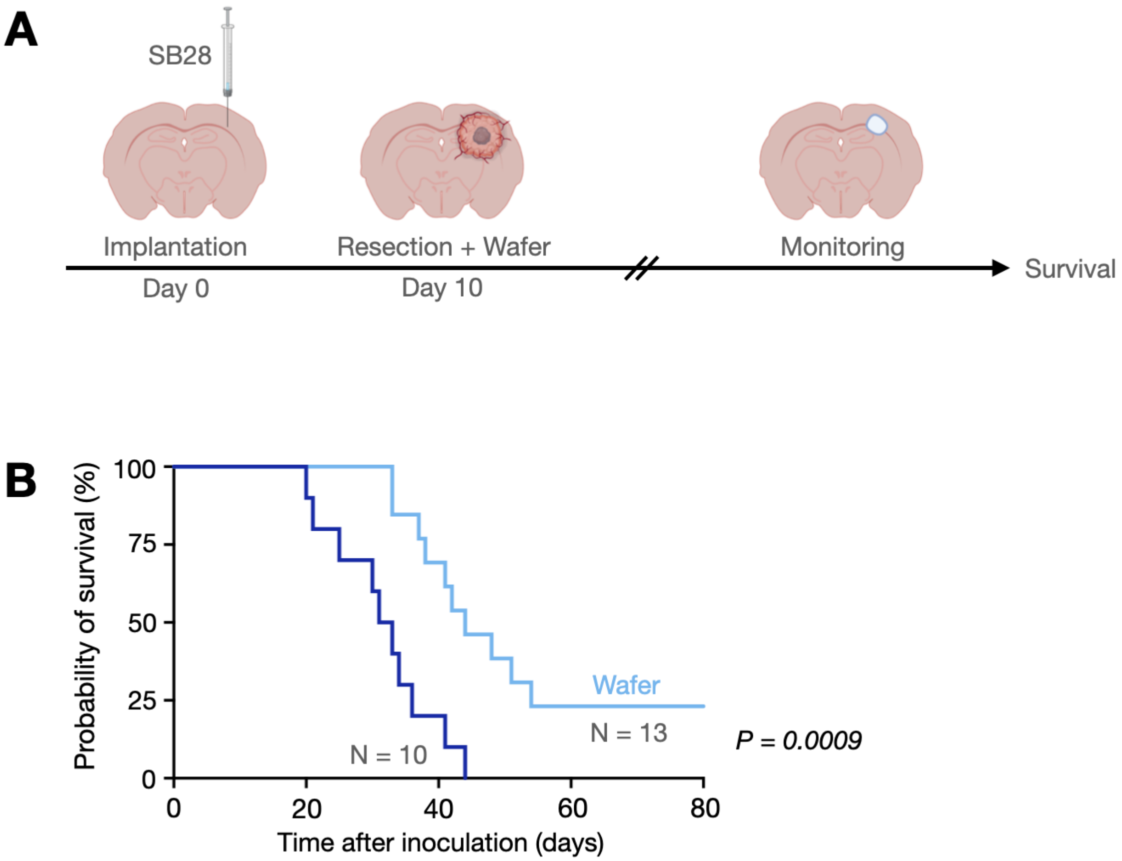

Fig. S17: Flow cytometry. A. Experimental outline of CT2a tumors, resection and wafer implantation, and processing of brain tumors for flow cytometry. Created in BioRender. Weissleder, R. (2025), https://BioRender.com/4dpd1c6. **B.** Gating strategy for flow cytometry. **C** and **D.** Quantification of brain tumor CD8 T cells (**C**) or CD4 T cells (**D**) expressed as a proportion of T-cells, from control unresected (n = 5 biological replicates), empty wafer (n = 5 biological replicates), and drug-loaded CANDI wafer (n = 6 biological replicates). One-way ANOVA with multiple comparisons. Data are presented as mean values ± SD.

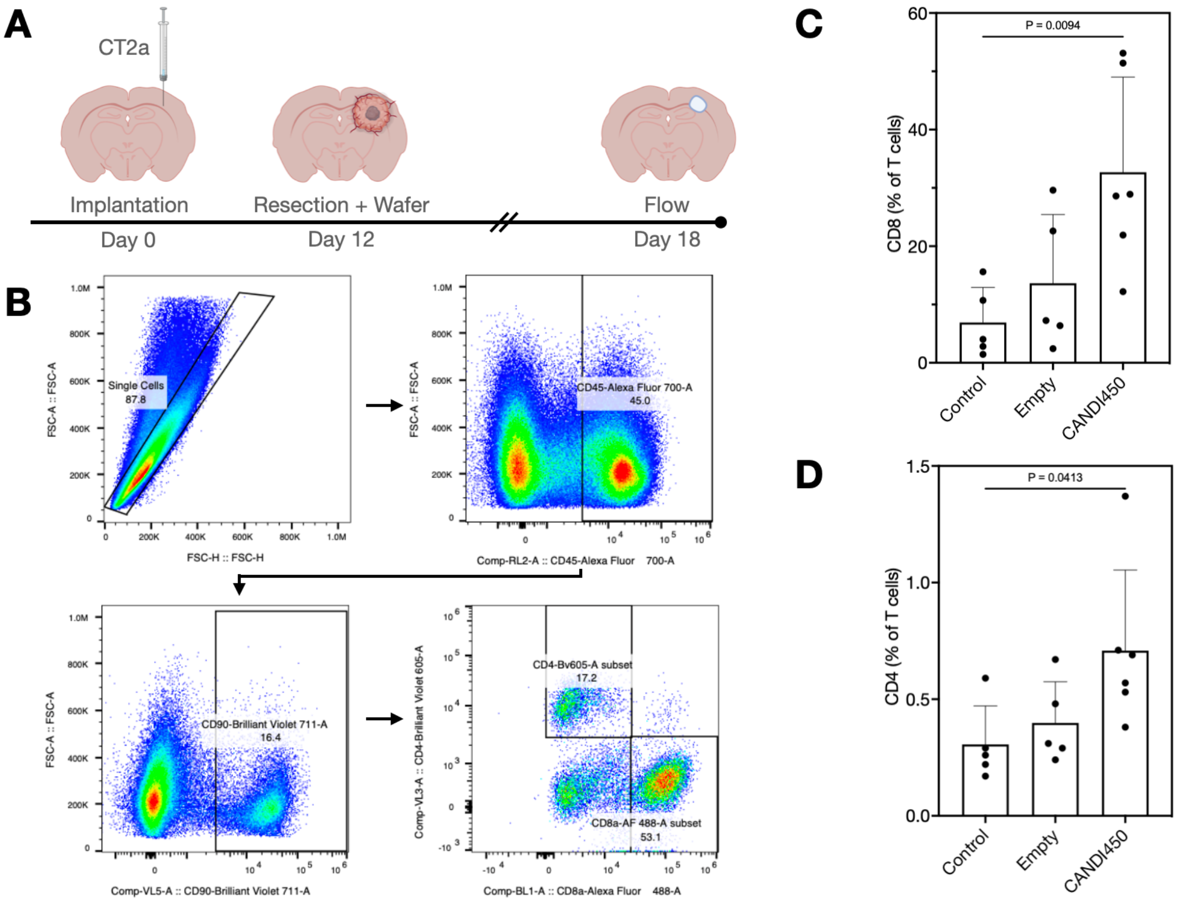

Fig. S18: TAM polarization assessed by flow cytometry. A. Experimental outline of CT2a tumor implantation, resection and wafer implantation, and processing of brain tumors for flow cytometry. Created in BioRender. Weissleder, R. (2025), https://BioRender.com/4dpd1c6. **B.** Gating strategy for flow cytometry. **C-F.** Quantification of expression levels of MHC-II, CD86, CD206, and TREM2 on F4/80-high macrophages in unresected control (n = 5 biological replicates), empty wafer (n = 5 biological replicates), and drug-loaded CANDI wafer (n = 6 biological replicates) indicating TAM polarization towards a pro-inflammatory and anti-tumorigenic M1 phenotype. One-way ANOVA with multiple comparisons. Data are presented as mean values ± SD.

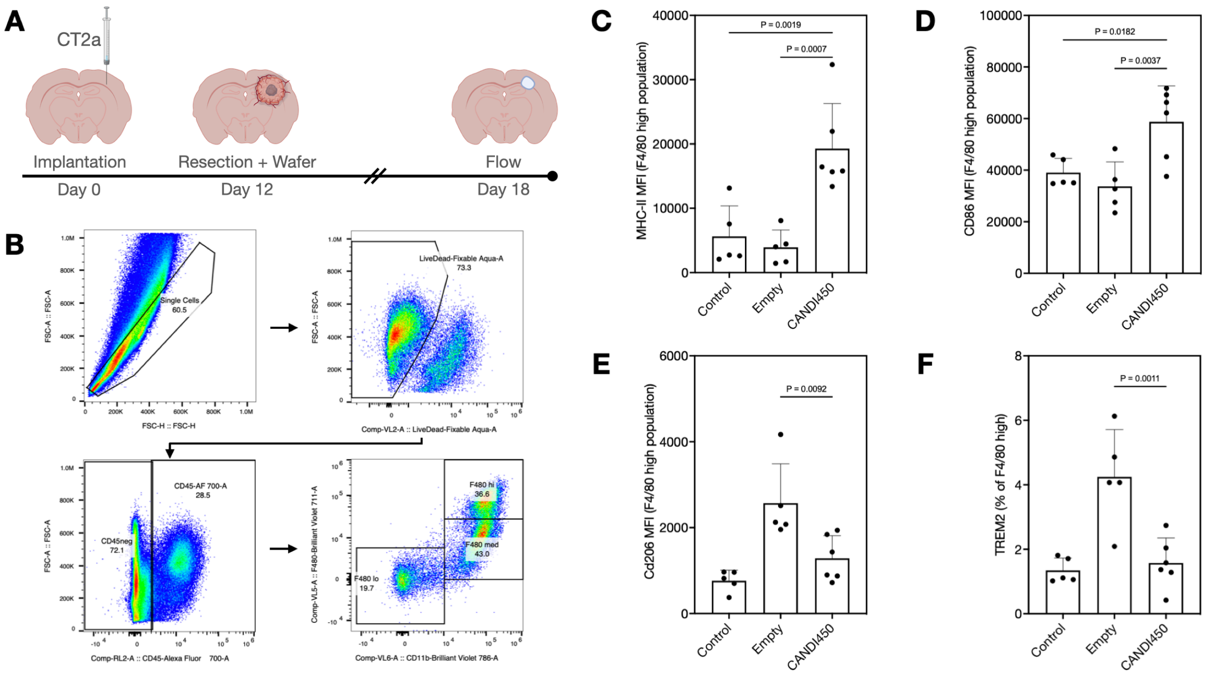

Fig. S19: Comparison of IHC immune cell profiling between different experimental groups. CT2A tumors were implanted intracranially and then processed for immunohistochemistry with subsequent quantification of the respective cell types without surgical resection (n = 3 biological replicates), after subtotal resection (n = 3 biological replicates), or after subtotal resection and packing with the CANDI wafer (n = 6 biological replicates). Note the significantly higher CD8 and CD4 populations in the wafer-treated group. One-way ANOVA with multiple comparisons. Data are presented as mean values ± SD.

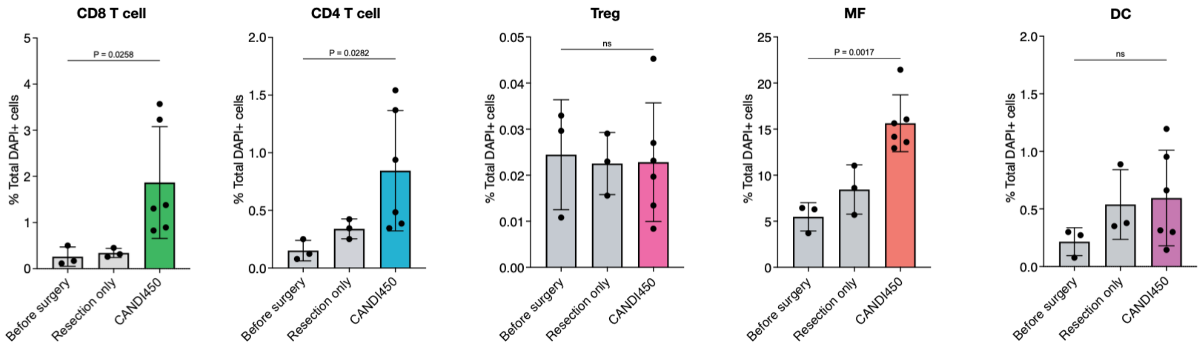

Fig. S20: Effects of CANDI wafer in human THP1-cells. To determine whether the wafer material would induce cytokines in human cells and tissue, we performed two experiments. **A.** A human monocyte/macrophage cell line (THP1) was exposed to CANDI wafer material, and IL12 levels were measured. Created in BioRender. Weissleder, R. (2025), https://BioRender.com/4dpd1c6. **B.** Significant IL12 induction could be observed ($p<0.001$ at 24 hrs), as well as no discernible toxicity. Additionally, myeloid cell activation marker analysis showed significant CD80 ($p=0.0232$) and CD86 ($p=0.0182$) upregulation upon treatment. n = 3 technical replicates. Two-way ANOVA with multiple comparisons. Data are presented as mean values ± SD.

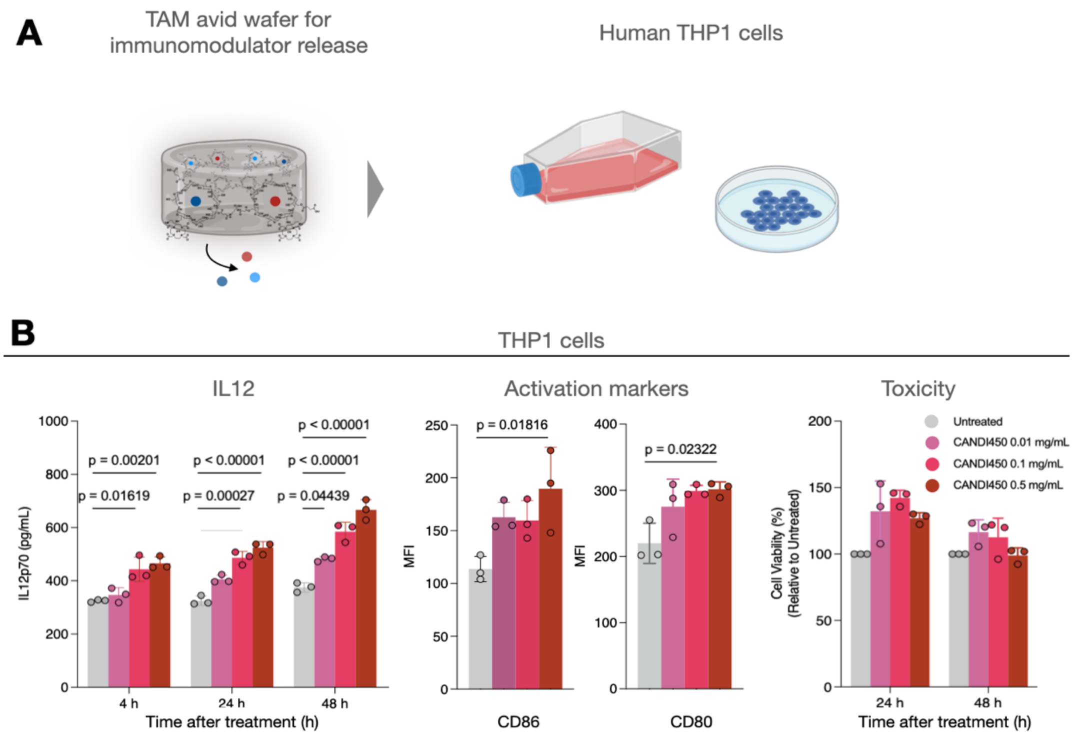

Fig. S21: Toxicity of drug-loaded wafer material. Viability assays were performed using immortalized macrophages (iMAC). Viability testing showed no appreciable toxicity effects at concentrations as high as 2.5mg/ml, much higher than the expected in-vivo concentration (< 1 mg/mL). n = 3 technical replicates. Data are presented as mean values ± SD.

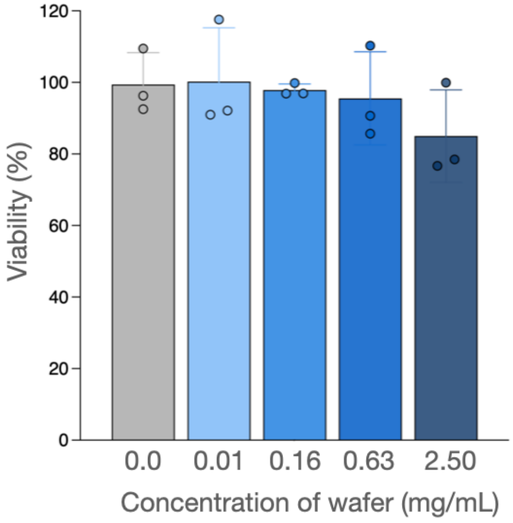

Fig. S22: Re-challenge experiment. To determine whether previously operated and CANDI-wafer-treated mice were protected from recurrence, we performed a re-challenge experiment by re-injecting $2x10^5$ tumor cells. Of the previously cured four mice that underwent re-challenge, three were completely tumor-free 22 days later, while one exhibited only minimal tumor regrowth. In contrast, all control animals (n = 4) that were previously resected but without wafer implantation developed significant tumor burden, with two animals requiring euthanasia by day 20. Previously resected animals treated with wafers thus seem to be protected from recurrence.

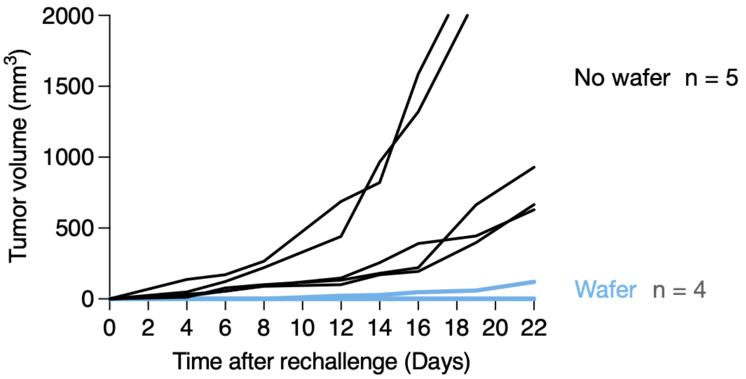

Fig. S23: Colorblind-accessible versions of microscopy images. A. Recolored images shown in Fig. 3A. **B.** Recolored images shown in Fig. 5B and S11. **C.** Recolored images shown in Fig. 5C. **D.** Recolored images shown in Fig. S11. Please see the respective figure legends for details.

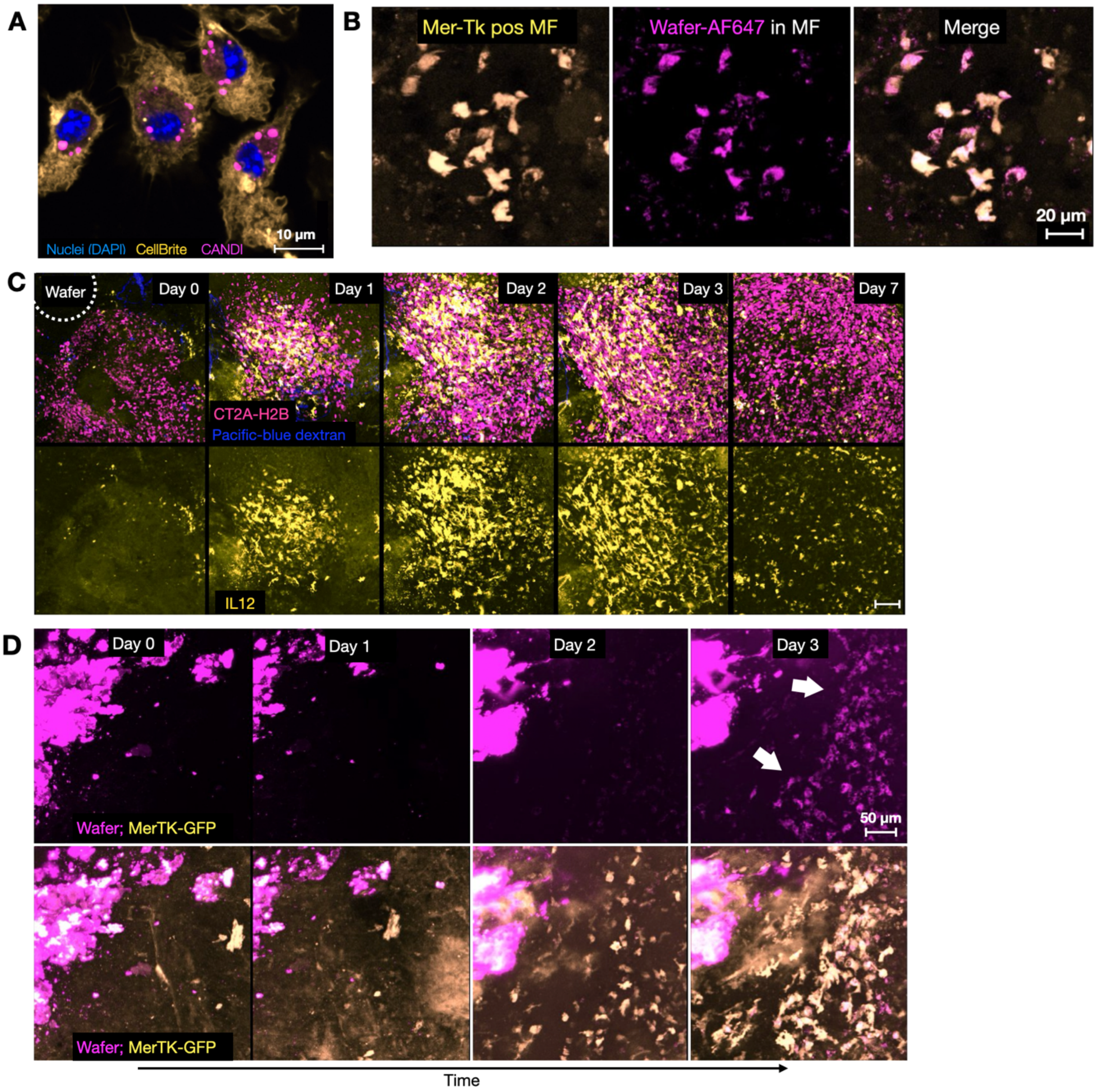

Supplement: Supplementary file 1 — Supplementary Figs. 1–23 [file 41551_2025_1533_MOESM1_ESM.pdf]
