# Supplementary material for: Targeting immunosuppressive myeloid cells via implant-mediated slow release of small molecules to prevent glioblastoma recurrence
Source: Nat Biomed Eng. 2025 Oct 22;10(7):1431–44. doi: 10.1038/s41551-025-01533-2 (PMC13150848; doi:10.1038/s41551-025-01533-2)
Supplement: Supplementary file 2 — Reporting Summary [file 41551_2025_1533_MOESM2_ESM.pdf]

nature portfolio

Corresponding author(s): Ralph Weissleder

Last updated by author(s): Jul 15, 2025

Reporting Summary

Nature Portfolio wishes to improve the reproducibility of the work that we publish. This form provides structure for consistency and transparency in reporting. For further information on Nature Portfolio policies, see our Editorial Policies and the Editorial Policy Checklist.

Statistics

For all statistical analyses, confirm that the following items are present in the figure legend, table legend, main text, or Methods section.

n/a	Confirmed	
☐	☒	The exact sample size (*n*) for each experimental group/condition, given as a discrete number and unit of measurement
☐	☒	A statement on whether measurements were taken from distinct samples or whether the same sample was measured repeatedly
☐	☒	The statistical test(s) used AND whether they are one- or two-sided<br>*Only common tests should be described solely by name; describe more complex techniques in the Methods section.*
☒	☐	A description of all covariates tested
☒	☐	A description of any assumptions or corrections, such as tests of normality and adjustment for multiple comparisons
☐	☒	A full description of the statistical parameters including central tendency (e.g. means) or other basic estimates (e.g. regression coefficient) AND variation (e.g. standard deviation) or associated estimates of uncertainty (e.g. confidence intervals)
☒	☐	For null hypothesis testing, the test statistic (e.g. *F*, *t*, *r*) with confidence intervals, effect sizes, degrees of freedom and *P* value noted<br>*Give P values as exact values whenever suitable.*
☒	☐	For Bayesian analysis, information on the choice of priors and Markov chain Monte Carlo settings
☒	☐	For hierarchical and complex designs, identification of the appropriate level for tests and full reporting of outcomes
☒	☐	Estimates of effect sizes (e.g. Cohen's *d*, Pearson's *r*), indicating how they were calculated

Our web collection on statistics for biologists contains articles on many of the points above.

Software and code

Policy information about availability of computer code

Data collection	Microscopy data were collected with CellCens Dimension (Olympus, version 3.2), FV10 (Olympus, version 4.1.1.5) or an Olympus BX-63 microscope (Metamorph Software version 7.10.4). Flow cytometry data was collected using the Attune Cytometric software (v.5.3.0).
Data analysis	Seurat (version 4), GraphPad Prism (version 10.0.2), ImageJ2 (version 2.3/1.53f), Microsoft Excel (v16.86), R (version 4.3.0, version 4.4.0), FlowJo (version 10.9.0), Horos (version 3.3.6) were used for data analysis. CellProfiler version 4.1.8 was used for the analysis of tissue section images. A custom python script was used to analyze IHC data and is available under https://csb.mgh.harvard.edu/bme_software.

For manuscripts utilizing custom algorithms or software that are central to the research but not yet described in published literature, software must be made available to editors and reviewers. We strongly encourage code deposition in a community repository (e.g. GitHub). See the Nature Portfolio guidelines for submitting code & software for further information.

Data

Policy information about availability of data

All manuscripts must include a data availability statement. This statement should provide the following information, where applicable:

- Accession codes, unique identifiers, or web links for publicly available datasets
- A description of any restrictions on data availability
- For clinical datasets or third party data, please ensure that the statement adheres to our policy

The data supporting the results in this study are available within the paper, the Supplementary Information, the Source data file or are available from the

corresponding author upon reasonable request. The publicly available scRNAseq datasets used in the bioinformatic analyses were accessed in the Gene Expression Omnibus under the accession code GSE197879 and in the European Genome-phenome Archive (EGA) under the accession code EGAD00001006778. The acquired RNAseq data are available under https://csb.mgh.harvard.edu/bme_software.

Research involving human participants, their data, or biological material

Policy information about studies with human participants or human data. See also policy information about sex, gender (identity/presentation), and sexual orientation and race, ethnicity and racism.

Reporting on sex and gender	No sex or gender-based analyses were performed, as only ex vivo GBM tissue from two male patients was used to assess wafer-induced immune activation.
Reporting on race, ethnicity, or other socially relevant groupings	No race, ethnicity, or other social categorization variables were used in this study, as it involved ex vivo GBM tissue from two individual patients without group-based comparisons
Population characteristics	The study used ex vivo glioblastoma tissue obtained from two adult male patients diagnosed with glioblastoma. One patient was 37 years old, and the other was 52 years old. No treatment history was available or considered relevant for the experimental objectives of this study.
Recruitment	Ex vivo glioblastoma tissue was obtained from two patients undergoing clinically indicated tumor resection, with informed consent for research use. Participants were not actively recruited for this study. Tissue availability was based on clinical scheduling and consent status.
Ethics oversight	This study was approved by the Human Research and Ethics Committee of the Geneva University in Switzerland (CCER 2022-02109 and 2023-01928).

Note that full information on the approval of the study protocol must also be provided in the manuscript.

Field-specific reporting

Please select the one below that is the best fit for your research. If you are not sure, read the appropriate sections before making your selection.

☒ Life sciences ☐ Behavioural & social sciences ☐ Ecological, evolutionary & environmental sciences

For a reference copy of the document with all sections, see nature.com/documents/nr-reporting-summary-flat.pdf

Life sciences study design

All studies must disclose on these points even when the disclosure is negative.

Sample size	Sample sizes were chosen to allow statistical comparison between treatment groups. No formal statistical methods were used to pre-determine sample size; instead, sizes were based on prior experience with similar experimental models, expected effect sizes, and feasibility constraints (e.g., animal availability, ethical considerations, imaging resources). For in vitro experiments, if not stated otherwise, a sample size of 3 replicates was used. For in-vivo experiments on the therapeutic effects as well as the mechanistic effects, the details regarding sample sizes are provided in the figure legends .
Data exclusions	No data were excluded from the experimental results or analyses.
Replication	The numbers of experimental replicates conducted are reported within the text, accompanying figures or under materials and methods.
Randomization	Mice were randomized into different groups before treatment. In the in-vitro experiments, cultured cells or other samples were randomly assigned to different experimental groups.
Blinding	Blinding was not performed as the work does not involve groups of participants.

Reporting for specific materials, systems and methods

We require information from authors about some types of materials, experimental systems and methods used in many studies. Here, indicate whether each material, system or method listed is relevant to your study. If you are not sure if a list item applies to your research, read the appropriate section before selecting a response.

Materials & experimental systems

n/a	Involved in the study
☐	☒ Antibodies
☐	☒ Eukaryotic cell lines
☒	☐ Palaeontology and archaeology
☐	☒ Animals and other organisms
☒	☐ Clinical data
☒	☐ Dual use research of concern
☒	☐ Plants

Methods

n/a	Involved in the study
☒	☐ ChIP-seq
☐	☒ Flow cytometry
☒	☐ MRI-based neuroimaging

Antibodies

Antibodies used

Antibody Application Target population Source Dye Catalog# Clone Dilution/Concentration
CD45 IF Hematopoetic Cells BioLegend AF488 368535 2D1 2 µg/ml
CD4 IF CD4 T Cells BioLegend AF488 100532 RM4-5 2 µg/ml
CD8 IF CD8 T Cells BioLegend AF750 100702 53-6.7 2 µg/ml
FoxP3 IF Regulatory T cells BioLegend AF647 126408 MF14 2 µg/ml
F4/80 IF Macrophage BioLegend AF488 123119 BM8 2 µg/ml
CD11c IF DC BioLegend APC 117310 N418 2 µg/ml
MHC-II IF Antigen-presenting cell BioLegend AF750 107603 M5/114.15.2 2 µg/ml
Ly-6C IF Monocyte BioLegend AF750 128003 HK1.4 2 µg/ml
TMEM119 IF Microglia Abcam AF647 ab209064 28-3 4 µg/ml
Anti-Rat antibody IF Rat IgG primary antibody Thermo DyLight 755 SA5-10015 Polyclonal 2 µg/ml
Streptavidin IF Biotin-conjugated primary antibody Thermo AF750 S21384 N/A 1 µg/ml
CD45 Flow Immune cells AF700 56-0451-82 30-F11 2 µl/ml
Dump (negative gate) CD19, B220, CD3e, NK1.1 mAbs Flow B, T, and NK cells Biolegend PE-Cy5 2 µl/ml
XCR1 Flow cDC1 Biolegend PE 148204 ZET 2 µl/ml
CD11c Flow Antigen-presenting cell Thermo PE-eF610 61-0114-82 N418 2 µl/ml
F4/80 Flow Macrophage Thermo PE-Cy7 BM8 2 µl/ml
IL-12 Flow IL-12 Producers Reporter Gene eYFP 2 µl/ml
CD80 Flow Co-stimulation Thermo APC 17-0801-82 16-10A1 2 µl/ml
MHC II Flow Antigen-presenting cell Thermo PE-Cy7 47-5321-82 M5/114.15.2 2 µl/ml
CD40 Flow Co-stimulation Biolegend Pacific Blue 124626 3/23 2 µl/ml
Live/Dead Aqua Amine Flow Live Dead Marker Thermo L34966 1 µl/ml
Sirpa Flow cDC2 BD Bv605 740390 P84 2 µl/ml
CD86 Flow Co-stimulation Biolegend Bv785 105043 GL-1 2 µl/ml
CD90 Flow T Cells Biolegend Bv711 105349 30-H12 2 µl/ml
The dilution used for each antibody is available in the respective table in the paper. 2 µl/ml

Validation

We used commercially available antibodies validated by the vendor for specific purposes. We provide vendor catalog number and/or clone number. In addition, we re-validated antibodies against positive and negative control proteins or cell lines as outlined elsewhere (Uhlen et al., 2016, Nat Methods, 13, 823-7).

Eukaryotic cell lines

Policy information about cell lines and Sex and Gender in Research

Cell line source(s)

CT-2A were obtained from Samuel Rabkin, MGB (PMID: 1418222). SB-28 were obtained from Hideho Okada, UCSF (PMID: 30524896). IMACs were received from Charles L. Evavold (PMID: 34289345). THP-1 were commercially purchased from ATCC (Catalog-Number: TIB-202). BMDMs were obtained from donor mice as described under materials and methods.

Authentication

Cell-lines were obtained as validated material and used directly without re-validation or storage.

Mycoplasma contamination

All cell-lines used tested negative for mycoplasma contamination.

Commonly misidentified lines
(See ICLAC register)

No commonly misidentified lines were used.

Animals and other research organisms

Policy information about studies involving animals; ARRIVE guidelines recommended for reporting animal research, and Sex and Gender in Research

Laboratory animals

Immunocompetent C57BL/6J wild-typ-mice (Strain Number: 000664, JAX, Bar Harbor, ME); MerTK-GFP mice (Strain Number: 036071, JAX, Bar Harbor, ME); IL12-eYFP mice (Strain Number: 006412, JAX, Bar Harbor, ME)
Light cycle: A 14-hour light/10-hour dark cycle or 12 light/12 dark cycle is used.
Temperature and humidity: Temperatures of 65-75°F (~18-23°C) with 40-60% humidity are used.

	Diet: Fat content ranges from 4% to 11%, Water is accessible at all times.
Wild animals	No wild animals were used.
Reporting on sex	For the therapeutic and mechanistic studies female mice were used.
Field-collected samples	No field-collected samples were used.
Ethics oversight	Ethics oversight was provided by the Institutional Animal Care and Use Committee (IACUC) for Massachusetts General Hospital, approved under Animal Protocol 2021N000135.

Note that full information on the approval of the study protocol must also be provided in the manuscript.

Plants

Seed stocks	*Report on the source of all seed stocks or other plant material used. If applicable, state the seed stock centre and catalogue number. If plant specimens were collected from the field, describe the collection location, date and sampling procedures.*
Novel plant genotypes	*Describe the methods by which all novel plant genotypes were produced. This includes those generated by transgenic approaches, gene editing, chemical/radiation-based mutagenesis and hybridization. For transgenic lines, describe the transformation method, the number of independent lines analyzed and the generation upon which experiments were performed. For gene-edited lines, describe the editor used, the endogenous sequence targeted for editing, the targeting guide RNA sequence (if applicable) and how the editor was applied.*
Authentication	*Describe any authentication procedures for each seed stock used or novel genotype generated. Describe any experiments used to assess the effect of a mutation and, where applicable, how potential secondary effects (e.g. second site T-DNA insertions, mosiacism, off-target gene editing) were examined.*

Flow Cytometry

Plots

Confirm that:

☒ The axis labels state the marker and fluorochrome used (e.g. CD4-FITC).

☒ The axis scales are clearly visible. Include numbers along axes only for bottom left plot of group (a 'group' is an analysis of identical markers).

☒ All plots are contour plots with outliers or pseudocolor plots.

☒ A numerical value for number of cells or percentage (with statistics) is provided.

Methodology

Sample preparation	Harvested tumors: Tissues were isolated, mechanically dissociated using surgical scissors, and digested using Collagenase IV at 0.2 mg/ml in RPMI 1640 at 37 °C for 45 minutes with vigorous shaking. After digestion, tissues were filtered through a 40 µM cell strainer and resuspended in protein-free PBS. Cells were stained using AquaAmine Live Dead Fixable viability stain (Thermo Fisher) and then washed with PBS. Cells were then resuspended in FACS buffer (PBS with 2 mM EDTA and 2% Fetal Calf Serum) and stained with Fc block (Biolegend) and fluorochrome-conjugated antibodies.<br>In-vitro experiments: Cells were stained using AquaAmine Live Dead Fixable viability stain (Thermo Fisher) and then washed with PBS. Cells were then resuspended in FACS buffer (PBS with 2 mM EDTA and 2% Fetal Calf Serum) and stained with Fc block (Biolegend) and fluorochrome-conjugated antibodies.
Instrument	Sample data were acquired using an Attune NxT flow cytometer (Thermo Fisher)
Software	Data were analyzed using FlowJo 10 software (TreeStar)
Cell population abundance	No cell-sorting or fractionation was performed.
Gating strategy	Immune cell analysis in the TME (Fig. S14): Single cells were first gated by forward and side scatter. Dead cells were excluded by Aqua Amine Live Dead fixable viability stain. CD45 positive cells and subsequently C D90 positive cells were gated and fractionated by expression of their respective marker subpopulations as indicated (Fig. S14). To analyze expression levels of CD206 and TREM2 on TAMs, after gating on CD45 we distinguished three macrophage populations (F4/80 low, medium, and high) and fractionated in the F4/80-high-population by expression of the respective marker subpopulation.<br>Mechanism of cellular uptake (Fig. 3B): Single cells were first gated by forward and side scatter. Dead cells were excluded by Aqua Amine Live Dead fixable viability stain. AF647-positive (CANDI-AF647) were then gated.<br>Wafer material distribution in the TME (Fig. 3C): Single cells were first gated by forward and side scatter. Dead cells were excluded by Aqua Amine Live Dead fixable viability stain. Next we gated on CD45 and for the CD45 high population the different immune cell subpopulations (CD11b, CD90.2, NK1.1, Ly6G, CD19). The CD45 low population was further fractionated into TMEM19+ (MG) and TMEM119- (CT2A) populations.<br>Immune cell analysis in the TME (Fig. S14): Single cells were first gated by forward and side scatter. Dead cells were excluded by Aqua Amine Live Dead fixable viability stain. CD45 positive cells and subsequently CD90 positive cells were gated and fractionated by expression of their respective marker subpopulations as indicated (Fig. S14). To analyze expression levels of

CD206 and TREM2 on TAMs, after gating on CD45 we distinguished three macrophage populations (F4/80 low, medium, and high) and fractionated in the F4/80-high-population by expression of the respective marker subpopulation.

☒ Tick this box to confirm that a figure exemplifying the gating strategy is provided in the Supplementary Information.
