# Supplementary material for: Targeting immunosuppressive myeloid cells via implant-mediated slow release of small molecules to prevent glioblastoma recurrence
Source: Nat Biomed Eng. 2025 Oct 22;10(7):1431–44. doi: 10.1038/s41551-025-01533-2 (PMC13150848; doi:10.1038/s41551-025-01533-2)
Supplement: Supplementary file 3 — Peer Review File [file 41551_2025_1533_MOESM3_ESM.pdf]

nature portfolio

Peer Review File

Targeting immunosuppressive myeloid cells via implant-mediated slow release of small-molecules to prevent glioblastoma recurrence

Corresponding Author: Dr Ralph Weissleder

Version 0:

Decision Letter:

Dear Dr Weissleder,

Thank you again for submitting to *Nature Biomedical Engineering* your manuscript, "Intracavitary polarization of myeloid cells controls postoperative glioblastoma recurrence". The manuscript has been seen by 4 experts; however, despite our chasing efforts 1 reviewer have yet to provide a report (should the reviewer provide it, we will send it to you in due course). You will find the reports of 3 reviewers at the end of this message, and please excuse the unusual delay in reaching a decision (1 reviewer needed additional time to assess the work and provide a report).

The reviewers recognize certain positive aspects of the work. However, they have raised concerns regarding the level of support for some claims, the degree of advancement over relevant published studies (including those from your own group), and have provided valuable suggestions for improvement. To address these concerns and strengthen the manuscript, substantial further effort will be required. Specifically, we expect you to address the mechanistic insufficiencies underlying the observed therapeutic outcomes, as highlighted by all reviewers. Furthermore, we also expect a thorough assessment of the long-term safety of the biomaterials, as explicitly requested by reviewer #1 and #2, and the incorporation of human data.

When you are ready to resubmit your manuscript, please upload the revised files, a point-by-point rebuttal to the comments from all reviewers, the reporting summary, and a cover letter that explains the main improvements included in the revision and responds to any points highlighted in this decision.

Please follow the following recommendations:

* Clearly highlight any amendments to the text and figures to help the reviewers and editors find and understand the changes (yet keep in mind that excessive marking can hinder readability).

* If you and your co-authors disagree with a criticism, provide the arguments to the reviewer (optionally, indicate the relevant points in the cover letter).

* If a criticism or suggestion is not addressed, please indicate so in the rebuttal to the reviewer comments and explain the reason(s).

* Consider including responses to any criticisms raised by more than one reviewer at the beginning of the rebuttal, in a section addressed to all reviewers.

* The rebuttal should include the reviewer comments in point-by-point format (please note that we provide all reviewers will the reports as they appear at the end of this message).

* Provide the rebuttal to the reviewer comments and the cover letter as separate files.

We expect that you will be able to resubmit the manuscript within 20 weeks of receiving this message. If this is the case, you will be protected against potential scooping. Otherwise, we will be happy to consider a revised manuscript as long as the significance of the work is not compromised by work published elsewhere or accepted for publication at *Nature Biomedical Engineering*.

We hope that you will find the referee reports helpful when revising the work. Please do not hesitate to contact me should

you have any questions.

Best wishes,

Filipe

__
Dr Filipe Almeida
Senior Editor, *Nature Biomedical Engineering*

____
Reviewer #1 (Report for the authors (Required)):

In this manuscript, the authors designed a drug-loaded guest-host macrostructure material for modulation of immunosuppressive microenvironment and controlled the postoperative recurrence of brain glioma. Three different immune modulatory drugs could be released locally and slowly to provide long-lasting conversion of myeloid phenotype. IL-12 produced by tumor antigen-presenting cells locally regulated cytolytic immunity and interferon gamma, thereby inhibiting tumor regeneration and prolonging animal survival.The characterization of drug-loaded implant materials is relatively complete, and cell experiments are also adequate. However, the drug delivery system has been reported in the author's previous studies. Although the drug delivery mode and animal model have changed, the therapeutic effect and mechanism have not been substantially improved, so the drug delivery system lacks innovation. At the same time, the design of animal experiments is deficient and lacks integrity. Therefore,it is not suitable for publication in its present state. There are the following concerns that need to be addressed.
1. The delivery system of CANDI wafer lacks innovation. In previous studies[ACS Nano 17, 20666-20679 (2023)], the authors had reported that the delivery system was used in the regulation of macrophage polarization and tumor immunosuppressive microenvironment, showing relatively good effects. In this manuscript, it is extended to the treatment of brain glioma, and there is no significant improvement in innovation. Moreover, the materials loaded with three drugs in the preliminary experiment were administered intravenously. The optimized drug loading mode was not necessarily suitable for local administration due to circulation and metabolism.
2. Although the authors verified the cell uptake on CANDI wafer and its mechanism, whether the delivery vector and drug are delivered to cells simultaneously remains to be further verified. Moreover, the authors mentioned that “The biggest inhibitory effects in uptake were observed with chlorpromazine, arguing for uptake via clathrin-mediated endocytosis”. If it is endocytic uptake, then the wafer material should be trafficked to endosome/lysosome and be degraded in low pH condition. And there is a question how to ensure the biological activity of the three drugs.
3. Animal experiments only included control group and experimental group treated with three drugs, and the other control groups were insufficient. Although the combined use of three drugs had good effects, different drugs had different mechanisms, so it was suggested to further investigate and optimize the dose and combination of drugs. The immunomodulatory mechanism of the combined use of the three drugs needs be further studied.
4. In Fig. 4B, the control group TMZ+cRT is lack, does the combination of TMZ+cRT have a significant effect on brain glioma? Is there a significant improvement after adding CANDI wafer? Lack of corresponding experimental evidence.
5. The original data of flow cytometry quantitative analysis of tumor tissue are recommended to be supplemented in SI.
6. It only verified the enhancement of IL-12 at the treatment site, but what about the expression of other immunostimulatory or immunosuppression-related cytokines?
7.Long-term biosafety needs to be supplemented.
8.Serial bioluminescence is necessary for tumor monitoring. FIg.4 described bioluminescence for tumor monitoring, but lacked the imaging data of bioluminescence.
Some important data was missing in Fig.6, for example “Following subtotal surgical resection and wafer implantation, we noted a significant increase in tumoral CD8 (7.2-fold; p=0.0258) and CD4 (5.6-fold; p=0.0282) levels, which was not observed in surgical resection alone.” The data of surgical resection alone group can not be found in the manuscript. “Temporal analysis of different cohorts also showed a 3-fold increase in TAM”. We haven't seen the raw data of TAM.

Reviewer #2 (Report for the authors (Required)):

This manuscript presents an approach to glioblastoma multiforme (GBM) management through the use of a synthetic immunomodulatory wafer designed to locally deliver immune-modulating drugs. The authors claim that this wafer leads to significant locoregional induction of IL-12 in myeloid cells, improved anti-tumor immunity, and extended survival in murine models. They propose that this method could represent a new and potent strategy for GBM control. The manuscript suffers from limitations in translational relevance, methodological robustness, and comprehensive understanding of the underlying mechanisms. Therefore, the manuscript is not suitable for publication in its current form and the enthusiasm for the manuscript at this stage is moderate to low.

1.The manuscript relies heavily on murine models, which have significant limitations in mimicking human GBM pathology and therapeutic responses. While the results are promising, they lack a direct translation to human clinical settings. The physiological and molecular differences between mice and humans, especially in the context of the blood-brain barrier (BBB) and immune responses, may affect the applicability of the findings.

2.The authors report a half-life of 8.9 days for wafer degradation in vivo, but this data is based on a limited number of experiments and may not accurately reflect long-term degradation and drug release kinetics.
3.The study does not thoroughly address potential local toxicity or side effects from the sustained drug release, which is crucial for clinical safety.
4.The manuscript acknowledges variability in therapeutic outcomes among the murine models but does not provide a robust analysis of the potential reasons for this variability. The potential influence of residual tumor volumes or differences in individual animal responses could significantly impact the overall efficacy and reliability of the wafer.
5.The manuscript presents survival data with some statistical significance but lacks comprehensive statistical analysis to fully validate the observed improvements. Additionally, the comparisons between different treatment groups and control conditions are not adequately detailed, raising concerns about the robustness and reproducibility of the results. And wafer alone and free drug should also be used to compare treatment effects.
6.The toxicity of Wafer's drug release is also something that should be monitored because high concentrations of the drug released locally cause changes in osmolality and the drug medication releases more than 20% of the drug within the first day, which implies an uncontrolled in vivo release.
7.While the study provides insights into local IL-12 induction and immune cell recruitment, it does not fully elucidate the detailed mechanisms by which the wafer induces these changes. There is insufficient exploration of potential off-target effects or the broader impact of the wafer on the tumor microenvironment.
8.The authors acknowledge the need for further research to optimize wafer design, explore additional drug payloads, and better understand the differences in drug release kinetics between mice and humans. However, these future directions are not sufficiently developed in the current study. Further investigations are necessary before the proposed wafer can be considered for clinical trials.

Reviewer #3 (Report for the authors (Required)):

The authors report utilization of a drug-loaded intracavitary wafer using murine glioma model to modulate the local GBM TME. Three drugs leveraging previous literature on the efficacy were combined for the wafer model, leading to synergistic effects. The data presented are convincing and suggest efficacy in the clinical realm.

Strengths
1. The use of two cell lines for validation (CT2A, SB28)
2. The synthesis and degradation profile of the wafer are well described with well-supported validation results.
3. Synergistic effect of the wafer when added to the standard-of-care (TMZ + radiation) suggest clinical relevance.

Weakness/suggestions
1. It is unclear when only half of the mice were cured, and the other half were not. A more detailed analysis between the two groups are warranted (imaging, harvest, histology, flow cytometry etc).
2. Lack use of human tissues and/or clear plan of how this approach can be applied in clinical domains and surgical feasibility
3. It highlights IL-12 as the central mechanism which is fairly well-supported but there is no description of potential off-target effects or compensatory mechanisms in other immune compartments
4. The wafer idea has been explored in the past (eg. Gliadel). Please discuss previous work in the field on this topic and elaborate why this method is distinctly unique.
5. While there was a significant survival benefit in the murine model, it is unclear why the benefit was seen in only half of the genetically same murine model. Have the authors investigated performing additional immunophenotyping of the mice who did vs did not achieve cure (possibly using the “fluid” that filled up the cavity)?
6. For the mice that achieve cure, before sacrificing them, can the authors perform a rechallenge experiment?
7. Figure 6 shows composition of the immune environment via flow cytometry but more detailed flow cytometric analysis can be performed – in addition to recruitment of CD4/CD8 T cells, how activated are they (using IFN-y or CD69)? TAM population goes up initially and then goes down. TAM population is a broad classification. Can expression of classic M1/M2 markers be also analyzed?
- Have the authors considered spatial transcriptomics to analyze for better understanding of TME before/after wafer implantation as well as to compare the cured vs non-responder mice?

Minor points
1. GBM as “glioblastoma multiforme” is an obsolete term as the nomenclature from WHO now describes it as “glioblastoma.”
2. Figure 1 needs a better descriptive title than “overview.”
3. Relevant figure panel will need to be referenced rather than just the figure number throughout the manuscript (e.g., Fig 1A-C instead of Fig 1)
4. “This triple combination was identified by a drug screen to maximize IL-12 production in myeloid cells when given systemically27.” Please clarify how the three agents were selected whether it was from the group’s screening or from previously published paper, etc.
5. “Marco (SCARA2, a scavenger receptor that could be a CANDI entry point), Cd209 (another receptor that could be a CANDI entry point)”. This requires additional explanation as to why these receptors can serve as entry points
6. Figure 4: add P values or asterisk to indicate significance for each survival curve
7. Figure 4 and Supplemental Fig S12-13 are redundant
8. Fig S1. “A. Umap of major immune cell types in CT-2A originally obtained by30.” “D. Umap of major immune cell types in human GBM originally obtained by31” --> sentence not completed

Version 1:

Decision Letter:

Dear Dr Weissleder,

Thank you for your revised manuscript, "Intracavitary polarization of myeloid cells controls postoperative glioblastoma recurrence", which has been seen by the original reviewers. In their reports, which you will find at the end of this message, you will see that the reviewers acknowledge the improvements to the work. However, Reviewer #1 and Reviewer #3 raise additional criticisms that we hope you will be able to address.

As before, when you are ready to resubmit your manuscript, please upload the revised files, a point-by-point rebuttal to the comments from all reviewers, the reporting summary, and a cover letter that explains the main improvements included in the revision and responds to any points highlighted in this decision.

As a reminder, please follow the following recommendations:

* Clearly highlight any amendments to the text and figures to help the reviewers and editors find and understand the changes (yet keep in mind that excessive marking can hinder readability).

* If you and your co-authors disagree with a criticism, provide the arguments to the reviewer (optionally, indicate the relevant points in the cover letter).

* If a criticism or suggestion is not addressed, please indicate so in the rebuttal to the reviewer comments and explain the reason(s).

* Consider including responses to any criticisms raised by more than one reviewer at the beginning of the rebuttal, in a section addressed to all reviewers.

* The rebuttal should include the reviewer comments in point-by-point format (please note that we provide all reviewers will the reports as they appear at the end of this message).

* Provide the rebuttal to the reviewer comments and the cover letter as separate files.

We expect that you will be able to resubmit the manuscript within 12 weeks of receiving this message. If this is the case, you will be protected against potential scooping. Otherwise, we will be happy to consider a revised manuscript as long as the significance of the work is not compromised by work published elsewhere or accepted for publication at *Nature Biomedical Engineering*.

We look forward to receive a further revised version of the work. Please do not hesitate to contact me should you have any questions.

Best wishes,

Filipe

__
Dr Filipe Almeida
Senior Editor, *Nature Biomedical Engineering*

____

Reviewer #1 (Report for the authors (Required)):

The authors have addressed most of my concerns, but some important points remain to be addressed.
1.Q2:The author does not explain the relevant mechanism clearly. If it is endocytic uptake, then the wafer material should be trafficked to endosome/lysosome and be degraded in low pH condition. How to achieve lysosomal escape so that the release of the drug can play its role? Further explanation and verification are required.
2.Q7:The authors mention that formal toxicity tests have been conducted, but relevant experimental data about long-term biosafety in vivo was not available.
3.Q8: Raw bioluminescence images are still lacking and it is not enough to provide quantitative analysis about

bioluminescence imaging data.

Reviewer #2 (Report for the authors (Required)):

The revised manuscript addresses several concerns raised in the initial review regarding the efficacy and translational relevance of the synthetic immunomodulatory wafer for GBM management. While the authors have made efforts to provide additional experimental data and clarifications, several critical issues remain unresolved, raising concerns about the robustness and clinical applicability of the study.
1. The authors suggest that the wafer is fully biodegradable based on a limited number of MRI imaging sessions. However, a comprehensive kinetic analysis of drug release over time is lacking. Without detailed pharmacokinetic and pharmacodynamic assessments, it is difficult to ascertain whether the observed immune responses are reproducible and sustainable. This is particularly important given the dynamic nature of the post-surgical microenvironment. Additionally, it would be beneficial for the authors to provide data on how the wafer's degradation profile may vary under different physiological conditions, as this information is crucial for assessing clinical translation.

2. The authors indicate that assessing local toxicity is challenging due to the inherent inflammation following surgery. While this point is understandable, a more rigorous evaluation of both local and systemic toxicity is necessary to ensure patient safety. The cell-based toxicity assays presented (Fig. S17 and S18) provide limited insight into the long-term effects of sustained drug release. Further, the RNA sequencing analysis (Fig. S7A) offers a narrow scope and does not sufficiently explore off-target immune responses or potential adverse effects. Including in vivo toxicity data would significantly strengthen the manuscript and address concerns regarding the safety profile of the wafer.

3. The statistical analysis remains incomplete and would benefit from greater clarity. Although the authors reference the use of Kaplan-Meier survival curves with log-rank tests, a more detailed explanation of the statistical methods used to assess variability and compare treatment groups is needed. Furthermore, the absence of comparisons between the drug-loaded wafer and free drug controls weakens the conclusions. While the authors cite IACUC restrictions as a reason for omitting these controls, such comparisons are essential to determine whether the observed therapeutic effects are attributable to the wafer formulation rather than drug release alone.

Reviewer #3 (Report for the authors (Required)):

All our comments have been addressed.

Version 2:

Decision Letter:

Dear Dr Weissleder,

Thank you for your revised manuscript, "Intracavitary polarization of myeloid cells controls postoperative glioblastoma recurrence". Having consulted with the original reviewers, I am pleased to write that we shall be happy to publish the manuscript in *Nature Biomedical Engineering*. Reviewer #3 has additional requests that we would like you to address. Could you please send me (via email) a response to the reviewer, as well as a new version of the manuscript incorporating the respective changes, while we prepare a checklist detailing our editorial and formatting requirements? Once they are ready, you will need to follow the instructions before you upload the final manuscript files.

Please do not hesitate to contact me if you have any questions.

Best wishes,
Filipe

—
Dr Filipe Almeida
Senior Editor, *Nature Biomedical Engineering*

____
Reviewer #1 (Report for the authors (Required)):

The authors have addressed my concerns.

Reviewer #2 (Report for the authors (Required)):

All our comments have been addressed.

Reviewer #3 (Report for the authors (Required)):

The authors have put in efforts to address the reviewer comments, some improvements in the current manuscript include the addition of human GBM tissue to enhance the translational value of the study and the discussion has been strengthened. Because of that, I am supportive of publication in Nature Biomedical Engineering. However, I suggest few additional improvements:
- There is a variability of treatment responses in the mice model (despite their genetic homogeneity). The authors do provide an explanation, but it is largely speculative. Could the authors provide a deeper discussion on how such variability can represent translational challenges, and how one can tackle them?

- Another concern is the novelty of the reported finding given that the group has recently published on the CANDI platform with IL-12 as the primary mechanism in tumor-associated myeloid cell population (Lugani et al 2023). This paper focus on the surgical applicability as a wafer system but an additional mechanistic exploration could have enhanced its novelty. I suggest that the authors specify what avenues of future research are needed to provide better understanding of the underlying therapeutic effects mediated from this strategy.

Minor Comments:
The authors need to clarify the number of patient samples obtained/used for the study and whether there was variability among patient samples.

Version 3:

Decision Letter:

Dear Dr Weissleder,

I am happy to inform you that your manuscript, "Targeting immunosuppressive myeloid cells via implant-mediated slow release of small-molecules to prevent glioblastoma recurrence", has now been accepted for publication in *Nature Biomedical Engineering*.

Over the next few weeks, the figures will be checked for production quality, the text edited to ensure that it conforms to house style, and the manuscript typeset.

Our Articles are published about 40 days after the acceptance date (we recommend that you inform your institutional press office of this timeframe), and you will be notified of the actual publication date a few days in advance. Articles can be published any working day of the week, and are pushed live shortly after 10 am London time.

Publishing agreement. You will be asked to digitally sign a publishing agreement (grant of rights). After the signed publishing agreement has been received, the proofs of the article will be sent to you for review. If you have any queries during the production process, or you cannot meet the requested deadline for returning the proofs, please contact rjsproduction@springernature.com.

Nature Biomedical Engineering is a Transformative Journal. Authors may publish their research with us through the traditional subscription access route, or make their paper immediately open access through payment of an article-processing charge. More information about publication options is available.

You may need to take specific actions to comply with funder and institutional open-access mandates. If the work described in the accepted manuscript is supported by a funder that requires immediate open access (as outlined, for example, by Plan S) and your manuscript was originally submitted on or after January 1st 2021, then you should select the gold OA route. Authors selecting subscription publication will need to accept our standard licensing terms (including our self-archiving policies), and these will supersede any other terms that the author or any third party may assert apply to any version of the manuscript.

Acceptance of your manuscript is conditional on agreement, by all authors, with both our <a

href="http://www.nature.com/authors/policies/embargo.html">media embargo and confidentiality and pre-publicity policies. In particular, you may arrange your own publicity of the Article (for instance, through your institutional press office), as long as you ensure that journalists strictly adhere to the media embargo.

To assist you in disseminating the work, as soon as the Article is published you will be able to take advantage of the Springer Nature SharedIt initiative to generate a unique shareable link to the Article that will allow anyone (with or without a subscription) to read it. Recipients of the link who are subscribers will also be able to download and print the PDF.

Thank you for having submitted this work to *Nature Biomedical Engineering*.

Best wishes,
Filipe

__
Dr Filipe Almeida
Senior Editor, *Nature Biomedical Engineering*

Open Access This Peer Review File is licensed under a Creative Commons Attribution 4.0 International License, which permits use, sharing, adaptation, distribution and reproduction in any medium or format, as long as you give appropriate credit to the original author(s) and the source, provide a link to the Creative Commons license, and indicate if changes were made.
In cases where reviewers are anonymous, credit should be given to 'Anonymous Referee' and the source.
The images or other third party material in this Peer Review File are included in the article's Creative Commons license, unless indicated otherwise in a credit line to the material. If material is not included in the article's Creative Commons license and your intended use is not permitted by statutory regulation or exceeds the permitted use, you will need to obtain permission directly from the copyright holder.
To view a copy of this license, visit https://creativecommons.org/licenses/by/4.0/

Reviewer #1:
In this manuscript, the authors designed a drug-loaded guest-host macrostructure material for modulation of immunosuppressive microenvironment and controlled the postoperative recurrence of brain glioma. Three different immune modulatory drugs could be released locally and slowly to provide long-lasting conversion of myeloid phenotype. IL-12 produced by tumor antigen-presenting cells locally regulated cytolytic immunity and interferon gamma, thereby inhibiting tumor regeneration and prolonging animal survival.The characterization of drug-loaded implant materials is relatively complete, and cell experiments are also adequate. However, the drug delivery system has been reported in the author's previous studies. Although the drug delivery mode and animal model have changed, the therapeutic effect and mechanism have not been substantially improved, so the drug delivery system lacks innovation. At the same time, the design of animal experiments is deficient and lacks integrity. Therefore,it is not suitable for publication in its present state. There are the following concerns that need to be addressed.

1. The delivery system of CANDI wafer lacks innovation. In previous studies[1], the authors had reported that the delivery system was used in the regulation of macrophage polarization and tumor immunosuppressive microenvironment, showing relatively good effects. In this manuscript, it is extended to the treatment of brain glioma, and there is no significant improvement in innovation. Moreover, the materials loaded with three drugs in the preliminary experiment were administered intravenously. The optimized drug loading mode was not necessarily suitable for local administration due to circulation and metabolism.

It is true that prior research had identified small molecule modulators to coax TAM into antitumor phenotypes; this, for example, results in IL12[1], CXCL9[2], or SPP1[3] pathways being activated or inhibited. These prior studies were primarily drug screens, and "hits" were incorporated into IV injectable nanoparticles about 30 nm in size. While this approach worked well for peripheral cancers, it did not work well for GBM; with only marginal therapeutic responses observed (**Fig. R1**), likely due to the well-documented challenges of drug delivery across the blood-brain barrier. Indeed, similar trends have been observed for immune checkpoint blockade agents, among many other drugs, making GBM one of the deadliest cancers.

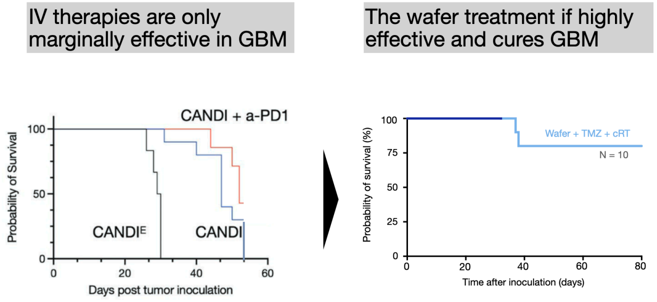

Fig. R1: IV-injected therapies done often do not work well in GBM because of delivery barriers (Nat Rev Canc 2020;20:26–41). The post-surgical wafer treatment on the contrary is highly effective

The novelty of the current study was that we developed a TAM-targeted immunomodulatory wafer, thus bypassing the delivery issue and, for the first time, show that long-term modulation of phagocytic cells in the brain of resected tumors leads to actual cures. We are not aware of any other therapeutic approach that has achieved

this complete cure in the very aggressive orthotopic CT2a and SB28 glioblastoma models.

Moreover, this wafer-based strategy aligns seamlessly with current GBM treatment workflows. In clinical practice, the vast majority of GBM patients undergo resection surgery (in combination with adjuvant radiation and chemotherapy), making an implantable immunomodulatory wafer an ideal intervention at the time of surgery. This aspect may not have been fully appreciated, so we would like to emphasize the clinical translatability of the concept, especially highlighting our added experiments in live human organotypic tissue confirming murine work (**Fig. 7, Fig. S17**).

2. Although the authors verified the cell uptake on CANDI wafer and its mechanism, whether the delivery vector and drug are delivered to cells simultaneously remains to be further verified. Moreover, the authors mentioned that “The biggest inhibitory effects in uptake were observed with chlorpromazine, arguing for uptake via clathrin-mediated endocytosis”. If it is endocytic uptake, then the wafer material should be trafficked to endosome/lysosome and be degraded in low pH condition. And there is a question how to ensure the biological activity of the three drugs.

The TAM internalized drugs clearly change the TAM phenotype in the TME as demonstrated by extensive experiments, including RNAseq (**Fig. 3**), intravital microscopy in reporter mice (**Fig. 5**), and cytokine profiling (**Fig. 3**), as well as flow cytometry of treated tumors (**Fig. S15**). To address this further and pinpoint specific pathways we also provide new ELISA evidence for pathway modulation (see **Fig R2**).

Figure Redacted

Fig. S15: TAM polarization assessed by flow cytometry. A. Experimental outline of CT2a tumor implantation, resection and wafer implantation, and processing of brain tumors for flow cytometry. **B.** Gating strategy for flow cytometry. **C-F.** Quantification of expression levels of MHC-II, CD86, CD206, and TREM2 on F4/80-high macrophages. indicating TAM polarization towards a pro-inflammatory and anti-tumorigenic M1 phenotype. One way ANOVA with multiple comparisons.

3. Animal experiments only included control group and experimental group treated with three drugs, and the other control groups were insufficient. Although the combined use of three drugs had good effects, different drugs had different mechanisms, so it was suggested to further investigate and optimize the dose and combination of drugs. The immunomodulatory mechanism of the combined use of the three drugs needs be further studied.

First and foremost, each of the three payload drugs has failed as single agent therapeutics in clinical trials because of lack of efficacy or toxicity. Second, in order to work at acceptable concentrations, these drugs rely upon synergism. Prior work has compared the efficacy of different combinations and doses, and we don't think that reiterating prior published work is helpful (other than referencing it, of course). **Fig. R3** summarizes some of the published data (PMID: 37824733)[1].

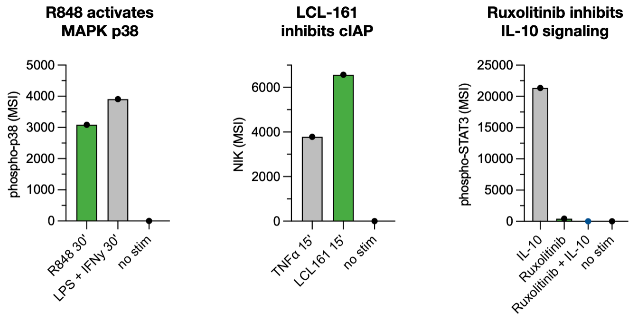

Fig. R2: Evidence that payloads affect specific pathways in TAM. MSI: mean signal intensity by Western blotting

4. In Fig. 4B, the control group TMZ+cRT is lack, does the combination of TMZ+cRT have a significant effect on brain glioma? Is there a significant improvement after adding CANDI wafer? Lack of corresponding experimental evidence.

The fig. 4B explores the synergistic effects of postoperative wafers with the addition of clinical standard-of-care therapy (TMZ + radiation). Both groups are shown. The probability of survival in our mouse cohort receiving TMZ+cRT was only 25%. More importantly, we show that TMZ and radiation with wafer further enhanced survival to ~75% of mice. In clinical practice, nearly all postoperative patients receiving TMZ+cRT will die within 2 years. Thus, providing peri-operative immunotherapy could substantially improve surgical outcomes.

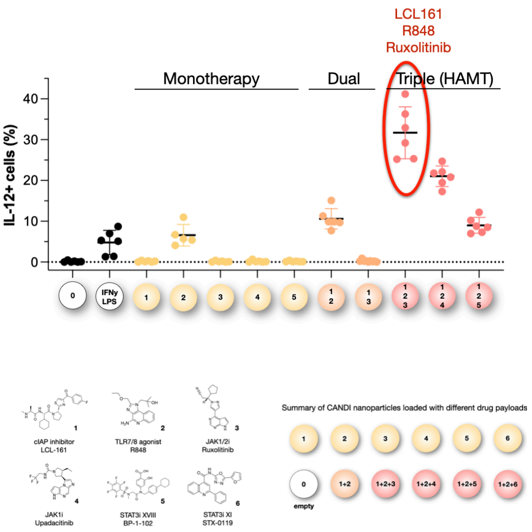

Fig. R3: Identification of triple combination

5. The original data of flow cytometry quantitative analysis of tumor tissue are recommended to be supplemented in SI.

The original flow cytometry data (gating strategy) is indeed part of the supplemental information (**Fig. S6**, **Fig. S14**, and **Fig. S15**).

6. It only verified the enhancement of IL-12 at the treatment site, but what about the expression of other immunostimulatory or immunosuppression-related cytokines?

We performed three types of experiments: i) window chamber experiments to study the temporal evolution of the key cytokine IL12 (**Fig 5C**); ii) temporal plasma level measurements of IL12 to rule out systemic effects (**Fig 5E**) and iii) comprehensive cytokine profiling (**Fig. 3E**) identifying IL12 as one of the top effectors. The reviewer points to exceptionally surgically challenging experiments in which we showed the temporal evolution of the key cytokine IL12, which is strongly linked to anti-tumor

efficacy, at the treatment site by implanting micronized wafers in glioblastoma of IL12-reporter-mice and subsequent temporal confocal imaging through implanted window chambers. In addition, we have performed comprehensive cytokine profiling (**Fig. 3E**) and RNAseq, identifying IL12 as one of the top effectors, and providing broader cytokine induction data. To provide further mechanistic proof of myeloid immunoconversion in-vivo, we now also provide data from flow-cytometric analysis of wafer-treated tumors, clearly indicating in-vivo TAM polarization towards a pro-inflammatory and anti-tumorigenic M1 phenotype (**Fig. S15**). Finally, the new human data shows similar effects (**Fig. 7** and **Fig. S17**).

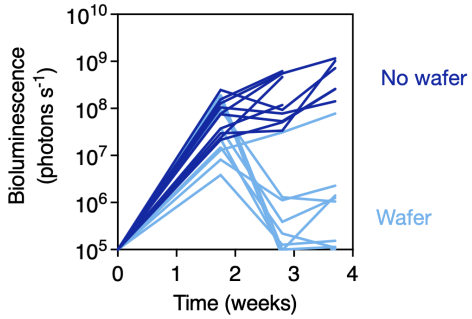

Fig. S12: Bioluminescence data on CT2A cohorts. All tumors were surgically resected at 12 days after implantation. In wafer-treated animals, the bioluminescence signal was much lower at 2 weeks after resection, with four out of nine animals back at baseline.

7. Long-term biosafety needs to be supplemented.
This is a good point. In preparation for a physician-sponsored FDA application, we had performed formal toxicity experiments. The experimental procedures were performed on the basis of standards set forth in: i) OECD No. 420, ‘Acute Oral Toxicity Study in Rodents’. The Organization for Economic Co-operation and Development (OECD) guidelines for the Testing of Chemicals, adopted by the council on December 17, 2001; ii) Exploratory IND Studies, Pharmacology/Toxicology; Guidance for Industry, Investigators, and Reviewers; U.S. Department of Health and Human Services, Food and Drug Administration, Center for Drug Evaluation and Research (CDER), January 2006; iii) OECD No. 407, ‘Repeated Dose 28-Day Oral Toxicity Study in Rodents’, The Organization for Economic Co-operation and Development (OECD) guidelines for the Testing of Chemicals, adopted on 3rd October 2008. The test compound was administered to Sprague-Dawley rats at 0.13 and 1.3 mg/kg, by intravenous route and did not result in mortality or clinical signs. There was no test item-related effect on body weight, feed consumption, hematology, clinical chemistry, organ weights, gross pathology, and histopathology in the rats.

8. Serial bioluminescence is necessary for tumor monitoring. Fig. 4 described bioluminescence for tumor monitoring, but lacked the imaging data of bioluminescence.
Thank you for this comment. We often use MR imaging or bioluminescence imaging to monitor tumor growth in orthotopic locations. We have now added the bioluminescence data to the supporting information (**Fig. S9**), showing similar data as in the Kaplan-Meier curve (**Fig. 4**).

9. Some important data was missing in Fig.6, for example “Following subtotal surgical resection and wafer implantation, we noted a significant increase in tumoral CD8 (7.2-fold; p=0.0258) and CD4 (5.6-fold; p=0.0282) levels, which was not observed in surgical resection alone.” The data of surgical resection alone group can not be found in the manuscript. “Temporal analysis of different cohorts also showed a 3-fold increase in TAM”. We haven't seen the raw data of TAM.

The purpose of **Fig. 6** is to show what happens during wafer treatment as a function of time. With wafer treatment, the CD8 and CD4 populations increase and then decrease with healing.

To further explore other treatment/control groups, we have now added **Fig. S16**, which shows higher CD8, CD4, and TAM levels in the wafer-treated animals compared to native tumors and surgical resection alone.

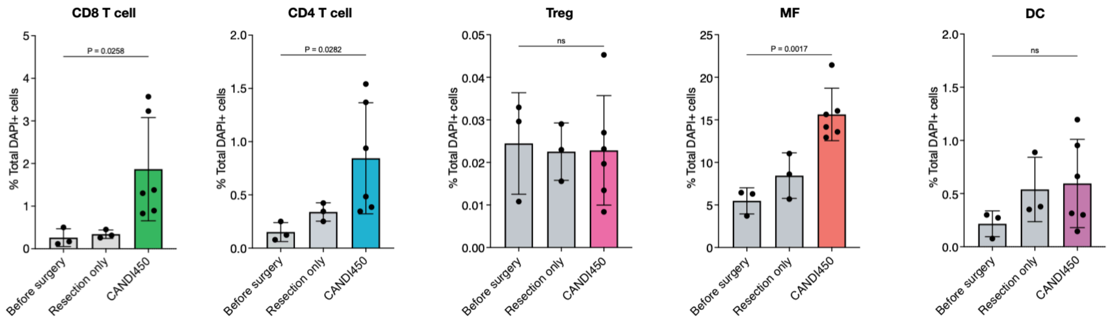

Fig. S16: Comparison of IHC immune cell profiling between different experimental groups. CT2A tumors were implanted intracranially and then processed for immunohistochemistry with subsequent quantification of the respective cell types without surgical resection, after subtotal resection, or after subtotal resection and packing with the wafer (CANDI450). Note the higher CD8 and CD4 populations with the wafer material.

Figure Redacted

Reviewer #2
This manuscript presents an approach to glioblastoma multiforme (GBM) management through the use of a synthetic immunomodulatory wafer designed to locally deliver immune-modulating drugs. The authors claim that this wafer leads to significant locoregional induction of IL-12 in myeloid cells, improved anti-tumor immunity, and extended survival in murine models. They propose that this method could represent a new and potent strategy for GBM control. The manuscript suffers from limitations in translational relevance, methodological robustness, and comprehensive understanding of the underlying mechanisms. Therefore, the manuscript is not suitable for publication in its current form and the enthusiasm for the manuscript at this stage is moderate to low.

1. The manuscript relies heavily on murine models, which have significant limitations in mimicking human GBM pathology and therapeutic responses. While the results are promising, they lack a direct translation to human clinical settings. The physiological and molecular differences between mice and humans, especially in the context of the blood-brain barrier (BBB) and immune responses, may affect the applicability of the findings. The reviewer is correct in stating that mice are not humans, but they are the closest thing we have in the GBM space, especially the tumor models CT2A and SB28, which we used in this study (PMID 34466804). To show that the wafer material described here has an effect on human cells, we performed two additional experiments. First, we obtained freshly resected human GBM specimens, incubated them with CANDI450, and ran IL-12 ELISA and immunohistochemistry. Second, we screened a primary human cell line that resembles macrophages (THP1) for the ability of the wafer to induce IL-12. Collectively, these data show that the wafer material indeed exerts effects on human cells and especially tumor-associated macrophages from the primary GBM setting. This

Fig. 7: Locoregional effects of the wafer on human GBM. A. Fresh resected human glioblastoma tissue was cut into 400 µm thick sections using a vibratome. Tissue slices were then incubated with CANDI450 wafer material. **B.** IL12 induction was measured as a function of dose and time. Note the highly significant IL12 induction in human GBM tissue within 24 hours ($p<0.001$) and the dose dependency. **C-D.** Immunohistochemistry performed in the same sections showed highly significant upregulation of myeloid cell activation markers HLA-DR, CD68, and CD86. For in-vitro wafer effects on human monocyte/macrophage THP-1 cells as well as toxicity testing, see **Fig. S17**.

new data has been added to the results section and the supplemental figures as **Fig. S17**.

CANDI wafer has immune stimulatory effects in human GBM tissue
To determine whether the CANDI wafer would have similar immune stimulatory effects in human tissue, we performed two additional experiments. First, we obtained freshly resected human glioblastoma tissue and exposed vibratome-cut organotypic tissue slices to different amounts of the wafer. Tissue culture supernatant was then tested for IL12 production (**Fig. 7B**). The data shows a clear dose and time-dependent increase in IL12 compared to control ($p<0.001$ at 24 hr). Similarly, we observed significant upregulation of TAM activation markers HLA-DR, CD68, and CD86 in tissue sections exposed to wafer material (**Fig. 7C-D**). Finally, we tested the wafer material in THP1 cells, showing similar IL12 induction effects, upregulation of myeloid cell activation markers, and no discernible toxicity in these human cells (**Fig. S17**).

2. The authors report a half-life of 8.9 days for wafer degradation in vivo, but this data is based on a limited number of experiments and may not accurately reflect long-term degradation and drug release kinetics.
The reviewer points to extraordinarily complex and tedious experiments, in which we had developed a novel Gd-containing CANDI wafer and then performed serial MRI after surgical resection of GBM in mice. Nevertheless, the data is based on dozens of imaging sessions in mice and is reproducible. We show that all wafer implants are fully biodegradable and that cured animals do not have any residual wafer material. We are not quite sure what else could be done to address this point.

Figure Redacted

Fig. S17: Effects of CANDI450 in human THP1-cells. To determine whether the wafer material would induce cytokines in human cells and tissue, we performed two experiments. **A.** A human monocyte/macrophage cell line (THP1) was exposed to CANDI450 wafer material, and IL12 levels were measured. **B.** Significant IL12 induction could be observed ($p<0.001$ at 24 hr), as well as no discernible toxicity. Additionally, myeloid cell activation marker analysis showed significant CD80 ($p=0.0232$) and CD86 ($p=0.0182$) upregulation upon treatment.

3. The study does not thoroughly address potential local toxicity or side effects from the sustained drug release, which is crucial for clinical safety.
The post-surgical space after GBM resection is a highly inflammatory and heterogenous space (physical manipulation, bleeding, cell death, residual tumor). We attempted to look at local toxicity but the effects are impossible to discern in the background of mechanical tissue injury. Conversely, the implantation of a wafer into a normal brain also results in mass effects so that molecular and cellular effects are not entirely distinguishable from the underlying injury. We performed conventional toxicity experiments on cells and did not observe any toxicity (**new Fig. S17 and S18**). Finally, we performed RNAseq of BMDM incubated with the wafer material and did not see any major gene upregulation as would be expected with toxicity (**Fig. S7A**).

4. The manuscript acknowledges variability in therapeutic outcomes among the murine models but does not provide a robust analysis of the potential reasons for this variability. The potential influence of residual tumor volumes or differences in individual animal responses could significantly impact the overall efficacy and reliability of the wafer.
This is a good point that we now address better in the discussion.

The current study had some limitations and these may represent avenues for future research. First, it remains unclear why some animals with glioblastoma were completely cured while others showed relapse. One interpretation could be that the relapses occurred in animals that had a larger fraction of unresected tumors, but this remained elusive as these deposits are too small (or too deep) to be imaged with reliability by BLI, MRI, or IVM, respectively. Alternatively, it is possible that near-complete tumor resection may not be ideal for wafer immunotherapy, as the antigens to prime an immune response against would be missing. Thus, the inherent variability in surgery, which has been a longstanding problem in the field, is likely the reason we observe divergent responses. While we attempted to perform the microsurgery in an identical fashion in the >140 operated animals, residual tumors may always be a possibility. Nonetheless, combined treatments with radiation and chemotherapy - which are standard clinical practice - could synergize with the wafer and reduce variability in outcomes. We indeed show that this was synergistic and resulted in even higher cures.

5. The manuscript presents survival data with some statistical significance but lacks comprehensive statistical analysis to fully validate the observed improvements. Additionally, the comparisons between different treatment groups and control conditions are not adequately detailed, raising concerns about the robustness and reproducibility of the results. And wafer alone and free drug should also be used to compare treatment effects.
The survival data was performed in n=90 mice and in two different models. To assess the statistical significance of the results, Kaplan-Meier curves were compared using the Log-rank (Mantel-cox) test, indicating significant differences in survival between the groups, which we have now better described in the main figures. Overall, over 140 animals were used for the study, including all the clinically relevant controls. While the suggestion for other controls is a reasonable one, our IACUC did not approve these requests as the free drugs had failed clinically (PMID: 17532523, 25113756, 34739635). Wafer alone did not have any effect (identical to the PBS control) and also did not change the macrophage gene signature (**Fig. S7**). Therefore, an empty wafer would never be used in a surgical setting unless drug-loaded. This is precisely what was tested.

Furthermore, systemically administered immunomodulators have largely failed for pharmacokinetic, pharmacodynamic or toxicity reasons[4-7]

6. The toxicity of Wafer's drug release is also something that should be monitored because high concentrations of the drug released locally cause changes in osmolality and the drug medication releases more than 20% of the drug within the first day, which implies an uncontrolled in vivo release.

Thank you for the comment on local toxicity. We now include **Fig. S18** with toxicity data showing that the material is safe over prolonged periods of time.

7. While the study provides insights into local IL-12 induction and immune cell recruitment, it does not fully elucidate the detailed mechanisms by which the wafer induces these changes. There is insufficient exploration of potential off-target effects or the broader impact of the wafer on the tumor microenvironment.

The reviewer brings up a good point. The main cytokine certainly appears to be IL12, but other pro-stimulatory cytokines are also elevated (**Fig. 3E**). The pathway mechanisms by which the drug-loaded wafer induces the pro-inflammatory changes are outlined in Fig. 3D. To provide further mechanistic insights into the effects the wafer induces in-vivo, we now also provide a more detailed flow-cytometric analysis of wafer-treated tumors (**Fig. S15**) showing the effects on the myeloid compartment indicative of anti-tumorigenic M1-polarization. The flow-cytometric data was further confirmed by more detailed IHC (**Fig. R4**). We point to the RNAseq data (**Fig. S7**), which showed no major effects of the wafer material alone.

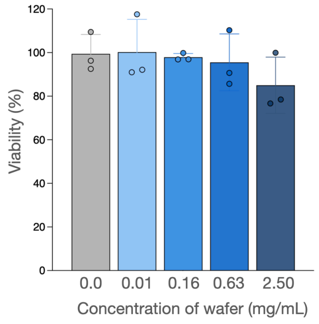

Fig. S18: Toxicity of drug-loaded wafer. Viability assays were performed using immortalized macrophages (iMAC). Viability testing showed no appreciable toxicity effects at concentrations as high as 2.5 mg/ml, much higher than the expected in vivo dose.

8. The authors acknowledge the need for further research to optimize wafer design, explore additional drug payloads, and better understand the differences in drug release kinetics between mice and humans. However, these future directions are not sufficiently developed in the current study. Further investigations are necessary before the proposed wafer can be considered for clinical trials.

That is a true statement, and we say so in the discussion:

In summary, we establish the myeloid compartment as a future drug target capable of preventing GBM recurrence if modulated appropriately. This work provides the rationale for more efficient immunotherapy in GBM and warrants clinical translation. Further studies are necessary before the proposed wafer can be considered for clinical trials.

Reviewer #3
The authors report utilization of a drug-loaded intracavitary wafer using murine glioma model to modulate the local GBM TME. Three drugs leveraging previous literature on the efficacy were combined for the wafer model, leading to synergistic effects. The data presented are convincing and suggest efficacy in the clinical realm. Strengths

- The use of two cell lines for validation (CT2A, SB28)
- The synthesis and degradation profile of the wafer are well described with well-supported validation results.
- Synergistic effect of the wafer when added to the standard-of-care (TMZ + radiation) suggest clinical relevance.

1. It is unclear when only half of the mice were cured, and the other half were not. A more detailed analysis between the two groups are warranted (imaging, harvest, histology, flow cytometry etc).
The reviewer raises a good point which is similar to that of reviewer #2. The problem is that one does not know which mouse will be cured and which not until it is too late and the non-survivor succumbs to cancer. The same happens with simple surgical resection, except that all animals will die, i.e. there is always residual cancer left behind.

One interpretation could be that the relapses occurred in animals that had a larger fraction of unresected tumors, but this remained elusive as these deposits are too small (or too deep) to be imaged with reliability by BLI, MRI, or IVM, respectively. Alternatively, it is possible that near-complete tumor resection may not be ideal for wafer immunotherapy, as the antigens to prime an immune response against would be missing. Thus, the inherent variability in surgery, which has been a longstanding problem in the field, is likely the reason we observe divergent responses. While we attempted to perform the microsurgery in an identical fashion in the >140 operated animals, residual tumors may always be a possibility. Nonetheless, combined treatments with radiation and chemotherapy - which are standard clinical practice - could synergize with the wafer and reduce variability in outcomes. We indeed show that this was synergistic and resulted in even higher cures.

2. Lack use of human tissues and/or clear plan of how this approach can be applied in clinical domains and surgical feasibility
This is a good point and is similar to that of reviewer 2, point 1. To show that the drug loaded-wafer material described here has an effect on human cells, we performed two additional experiments. <u>First</u> we screened human macrophage cells (THP, primary bone marrow isolate) for the ability of the wafer to induce IL-12 (and other cytokines). Second, we obtained freshly resected human GBM slices, incubated them CANDI, and ran IL-12 ELISA/IHC for immune co-stimulatory markers. Collectively, this data shows that the wafer material indeed exerts effects on human cells and especially tumor-associated macrophages. The new data has been added to the manuscript as **Fig. 7 and Fig. S17** (see new data above).

3. It highlights IL-12 as the central mechanism which is fairly well-supported but there is no description of potential off-target effects or compensatory mechanisms in other immune compartments

The reviewer brings up a good point. The main cytokine certainly appears to be IL12 but other pro-stimulatory cytokines are also elevated (**Fig. 3E**). We point to the RNAseq data (**Fig. S7**) which did not show any major effects of the wafer material alone. However, we feel that identifying and testing additional mechanisms would be beyond the scope of this manuscript.

4. The wafer idea has been explored in the past (eg. Gliadel). Please discuss previous work in the field on this topic and elaborate why this method is distinctly unique.

Thank you for this suggestion. We have added this aspect to the Discussion

……Myeloid cells in GBM rapidly adopt an immunosuppressive phenotype[8-10]. We reasoned that an immunomodulatory wafer material could be constructed to shift immune suppression to stimulation. Wafers have been used extensively to deliver drugs to GBM cells, e.g. carmustine (Gliadel)[11,12] and other drugs[13]. However, to our knowledge, they have not been used for direct innate immunomodulation. Here, we show the synthesis, characterization, and therapeutic efficacy of this approach. Using two different mouse models, we show anti-tumor immune responses preventing postoperative glioma recurrence.

5. While there was a significant survival benefit in the murine model, it is unclear why the benefit was seen in only half of the genetically same murine model. Have the authors investigated performing additional immunophenotyping of the mice who did vs did not achieve cure (possibly using the "fluid" that filled up the cavity)?

The reviewer raises a similar point as #1 above. We asked the same question and performed histology and plasma analysis to identify predictive biomarkers. Unfortunately, we were not able to identify any reproducible biomarkers. The fluid in the cavity is essentially CSF, and its analysis did not reveal any distinguishing features.

6. For the mice that achieve cure, before sacrificing them, can the authors perform a rechallenge experiment?

We have done this experiment and show that the mice are protected from rechallenge (new **Fig. S19**). The new experiments have been added to the manuscript.

Finally, to assess whether CANDI450 treatment induced antitumor immunity, we re-challenged previously cured mice with repeat tumor implantation. By day 22 after re-challenge, three

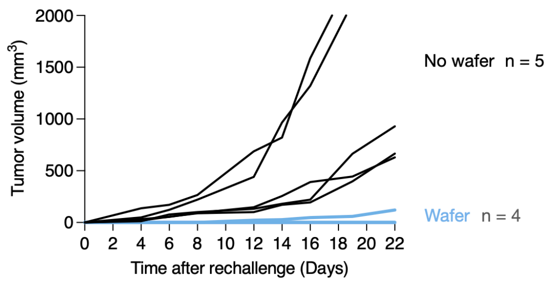

Fig. S19: Re-challenge experiment. To determine whether previously operated and CANDI450-treated mice were protected from recurrence, we performed a re-challenge experiment by re-injecting $2x10^5$ tumor cells. Of the previously cured four mice that underwent re-challenge, three were completely tumor-free 22 days later, while one exhibited only minimal tumor regrowth. In contrast, all control animals that were previously resected but without wafer implantation developed significant tumor burden, with two animals requiring euthanasia by day 20. Previously resected animals treated with wafers thus seem to be protected from recurrence.

of four mice remained completely tumor-free, while one exhibited only minimal tumor regrowth. In contrast, all control mice that received de novo flank tumor implantation developed significant tumor burden, suggesting that wafer immunoconversion generates a lasting immune response capable of preventing tumor recurrence.

7. Figure 6 shows composition of the immune environment via flow cytometry but more detailed flow cytometric analysis can be performed – in addition to recruitment of CD4/CD8 T cells, how activated are they (using IFN-y or CD69)? TAM population goes up initially and then goes down. TAM population is a broad classification. Can expression of classic M1/M2 markers be also analyzed? Have the authors considered spatial transcriptomics to analyze for better understanding of TME before/after wafer implantation as well as to compare the cured vs non-responder mice?
Indeed, we have also conducted a more detailed flow-cytometric analysis using resected tumor residuals after CANDI450 wafer treatment (**Fig. S15**). These experiments' results clearly indicate TAM polarization towards a pro-inflammatory and anti-tumorigenic M1 phenotype. We noted a significant increase in the expression of CD86 (p=0.0182), and MHC-II (p=0.0019) on F4/80^{high} macrophages, compared to native tumors before resection as well as to surgical resection alone without CANDI450 wafer treatment. We also noted a significant down-regulation of CD206 (p=0.0092) and TREM2 (p=0.0011) expression when compared to animals that only received surgery (**Fig. S15**).

Further IHC analysis (**Fig. R4**) also confirms TAM polarization towards an M1 phenotype and T cell activation. By IHC, we observed significantly higher MHCII expression in TAMs (p=0.032) as well as a decrease in expression levels of M2 phenotype markers, namely CD163 (p=0.0082) and CD206. We also determined the expression levels of Granzyme B in CD63+ T cells which were significantly increased (p=0.047) in CANDI450-treated tumors compared to control. This indicates that not only does the tumor infiltration of T cells increase, but also that the cytotoxic activity of T cells is elevated.

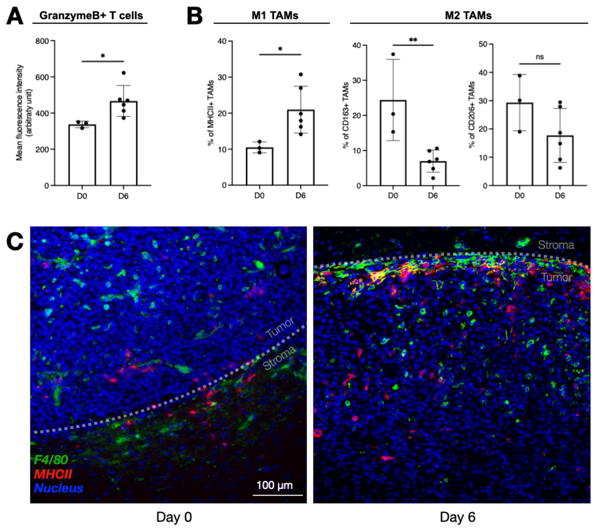

Fig. R4: Further detailed IHC analysis

Spatial transcriptomics holds promise for future research to gain a more comprehensive understanding of immune infiltration characteristics in tumors treated with CANDI wafers. A preliminary spatial analysis of the immune infiltration showed that TAMs are more densely located at the tumor boundary region in the treated tumors as compared to control group (**Fig. R4**). In addition, a larger fraction of the TAMs in the tumor boundary region expressed MHCII.

8. GBM as “glioblastoma multiforme” is an obsolete term as the nomenclature from WHO now describes it as “glioblastoma.”
This has been corrected (GBM = glioblastoma).

9. Figure 1 needs a better descriptive title than “overview.”
We have revised the legend.

Fig. 1: Glioblastoma is rich in immunosuppressive TAM. A-B. Synthetic spatial maps of myeloid cell distribution in CT-2A. The white grids represent 63 separate fields of view imaged to acquire the entire tissue specimen. Note that ~25% of all cells in CT-2A GBM represent macrophages.

10. Relevant figure panel will need to be referenced rather than just the figure number throughout the manuscript (e.g., Fig 1A-C instead of Fig 1)
We have updated the numbering throughout the entire manuscript.

11. “This triple combination was identified by a drug screen to maximize IL-12 production in myeloid cells when given systemically27.” Please clarify how the three agents were selected, whether it was from the group’s screening or from a previously published paper, etc.
It was identified through our own drug screens and this is now appropriately referenced.

The three molecules included a Janus Tyrosine Kinase (JAK) inhibitor (ruxolitinib), a cIAP inhibitor (LCL-161), and a toll-like receptor TLR7/8 agonist (R848). This triple combination was identified by a drug screen to maximize IL-12 production in myeloid cells when given systemically[1]. Additional prior work had identified R848 and LCL-161 as being able to modulate TAM phenotypes when used as monotherapy[14] or dual therapy[15] in peripheral cancers.

12. “Marco (SCARA2, a scavenger receptor that could be a CANDI entry point), Cd209 (another receptor that could be a CANDI entry point)”. This requires additional explanation as to why these receptors can serve as entry points
Thank you for this point which is now explained better in two different locations.

We next determined the mechanism of cellular CANDI wafer uptake using typical inhibitors of different pathways (chlorpromazine for clathrin-mediated endocytosis; wortmannin for micropinocytosis/phagocytosis; Imipramine for macropinocytosis; EIPA (5-(N-ethyl-N-isopropyl)amiloride) for macropinocytosis via Na/H exchange; **Fig. 3B** and **Fig. S6**). The biggest inhibitory effects in uptake were observed with chlorpromazine, arguing for uptake via clathrin-mediated endocytosis. Additionally, we noticed that Marco (SCARA2) and Cd209 were highly upregulated and could be CANDI entry points.

Among the top upregulated genes were: IL-12 (the main mechanism of action), Marco (SCARA2, a scavenger receptor that assists in clearing cellular debris and being a co-receptor to TLR modulating immune response), Cd209 (DC-SIGN, a C-type lectin

receptor that facilitates the uptake of carbohydrate-rich microorganisms and materials), and H2-M2 (antigen presentation, MHC).

13. Figure 4: add P values or asterisk to indicate significance for each survival curve
This has been added.

14. Figure 4 and Supplemental Fig S12-13 are redundant
Fig. 4 is the CT2A model and Fig. 13 is the SB28 model. The old Fig. S12 has been replaced with new data.

15. Fig S1. “A. Umap of major immune cell types in CT-2A originally obtained by30.” “D. Umap of major immune cell types in human GBM originally obtained by31” --> sentence not completed
This has been corrected

Fig. S1: Comparison of the TME in CT-2A (A-C) and human glioblastoma (D-F). A. Umap of major immune cell types in CT-2A originally obtained by[9]. Note the prominent myeloid cell population comprising primarily macrophages and monocytes. The microglia and lymphoid populations are small. **B.** Violin plots show expression of Trem2 and Spp1 genes in different clusters. SPP1, in particular, has recently been identified as an immunosuppressive biomarker[16]. **C.** Gene set enrichment analysis for the Monocyte, TAM and Microglia clusters. Hallmark pathways invariably involve NFkB and hypoxia. **D.** Umap of major immune cell types in human GBM originally obtained by[10]. The myeloid compartment is still sizable but the microglial compartment is larger than in the mouse. **E.** SPP1 and TREM2 levels across human cell types. Note higher SPP1 levels than in the mouse. **F.** Gene set enrichment analysis in human glioblastoma. Hallmark pathways upregulated include complement, inflammatory response, and NFkB signaling.

References cited in the point-by-point response

1. Fredrich, I. R. *et al.* Highly Active Myeloid Therapy for Cancer. *ACS Nano* **17**, 20666-20679 (2023).
2. Enbergs, N. *et al.* Pharmacological Polarization of Tumor-Associated Macrophages Toward a CXCL9 Antitumor Phenotype. *Adv Sci (Weinh)* e2309026 (2024).
3. Kartal, B. *et al.* Targeted SPP1 Inhibition of Tumor-Associated Myeloid Cells Effectively Decreases Tumor Sizes. *Adv Sci (Weinh)* e2410360 (2024).
4. Pockros, P. J. *et al.* Oral resiquimod in chronic HCV infection: safety and efficacy in 2 placebo-controlled, double-blind phase IIa studies. *J Hepatol* **47**, 174-182 (2007).
5. Infante, J. R. *et al.* Phase I dose-escalation study of LCL161, an oral inhibitor of apoptosis proteins inhibitor, in patients with advanced solid tumors. *J Clin Oncol* **32**, 3103-3110 (2014).
6. Johnson, M. L. *et al.* A Phase Ib Dose-Escalation Study of LCL161 Plus Oral Topotecan for Patients With Relapsed/Refractory Small Cell Lung Cancer and Select Gynecologic Malignancies. *Oncologist* **28**, 640-e559 (2023).
7. Bonneville-Levard, A. *et al.* Molecular profile to guide personalized medicine in adult patients with primary brain tumors: results from the ProfiLER trial. *Med Oncol* **39**, 4 (2021).
8. Miller, T. E. *et al.* Programs, Origins, and Niches of Immunomodulatory Myeloid Cells in Gliomas. *bioRxiv* 2023.10.24.563466 (2023).

9. Tomaszewski, W. H. *et al.* Neuronal CaMKK2 promotes immunosuppression and checkpoint blockade resistance in glioblastoma. *Nat Commun* **13**, 6483 (2022).
10. Pombo Antunes, A. R. *et al.* Single-cell profiling of myeloid cells in glioblastoma across species and disease stage reveals macrophage competition and specialization. *Nat Neurosci* **24**, 595-610 (2021).
11. Subach, B. R. *et al.* Morbidity and survival after 1,3-bis(2-chloroethyl)-1-nitrosourea wafer implantation for recurrent glioblastoma: a retrospective case-matched cohort series. *Neurosurgery* **45**, 17-22; discussion 22 (1999).
12. Gururangan, S. *et al.* Phase I study of Gliadel wafers plus temozolomide in adults with recurrent supratentorial high-grade gliomas. *Neuro Oncol* **3**, 246-250 (2001).
13. Erthal, L. C. S., Gobbo, O. L. & Ruiz-Hernandez, E. Biocompatible copolymer formulations to treat glioblastoma multiforme. *Acta Biomater* **121**, 89-102 (2021).
14. Rodell, C. B. *et al.* TLR7/8-agonist-loaded nanoparticles promote the polarization of tumour-associated macrophages to enhance cancer immunotherapy. *Nat Biomed Eng* **2**, 578-588 (2018).
15. Koch, P. D., Rodell, C. B., Kohler, R. H., Pittet, M. J. & Weissleder, R. Myeloid Cell-Targeted Nanocarriers Efficiently Inhibit Cellular Inhibitor of Apoptosis for Cancer Immunotherapy. *Cell Chem Biol* **27**, 94-104.e5 (2020).
16. Bill, R. *et al.* CXCL9:SPP1 macrophage polarity identifies a network of cellular programs that control human cancers. *Science* **381**, 515-524 (2023).

Reviewer #3 (Report for the authors (Required)):

The authors have put in efforts to address the reviewer comments, some improvements in the current manuscript include the addition of human GBM tissue to enhance the translational value of the study and the discussion has been strengthened. Because of that, I am supportive of publication in Nature Biomedical Engineering. However, I suggest few additional improvements:

1. There is a variability of treatment responses in the mice model (despite their genetic homogeneity). The authors do provide an explanation, but it is largely speculative. Could the authors provide a deeper discussion on how such variability can represent translational challenges, and how one can tackle them?
We have added a new paragraph to address future mechanistic explorations:

Although the mice used for studies are genetically homogeneous, some limitations still exist in complex *in vivo* animal models of cancer that could explain the variability in treatment responses seen. These sources of variation can include tumor location, differences in cell numbers of implantations, size at the start of treatment, and the degree of tumor resection efficiency. Precision in the surgical removal of tumors and the subsequent placement of immune stimulatory wafers may be a key determinant of translational success as these intraoperative immunotherapies progress. Innovations such as fluorescence-guided surgery for more defined tumor resection and tracking immune stimulatory material distribution to immune active sites, such as the dura and cervical lymph nodes, could be used to optimize the immune-activating impact of intraoperative biomaterials.

2. Another concern is the novelty of the reported finding given that the group has recently published on the CANDI platform with IL-12 as the primary mechanism in tumor-associated myeloid cell population (Lugani et al 2023). This paper focus on the surgical applicability as a wafer system but an additional mechanistic exploration could have enhanced its novelty. I suggest that the authors specify what avenues of future research are needed to provide better understanding of the underlying therapeutic effects mediated from this strategy.
This exact point was already addressed in our first revision. It is true that prior research had identified small molecule modulators to coax TAM into antitumor phenotypes; this,

for example, results in IL12[Fredrich et al., 2023, ACS Nano, 17, 20666-20679], CXCL9[Enbergs et al., 2024, Adv Sci (Weinh), e2309026], or SPP1[Kartal et al., 2024, Adv Sci (Weinh), e2410360] pathways being activated or inhibited respectively. These prior studies were primarily drug screens, and “hits” were incorporated into IV injectable nanoparticles about 30 nm in size. While this approach worked well for peripheral cancers, it did not work well for GBM; with only marginal therapeutic responses observed (**Fig. R1**), likely due to the well-documented challenges of drug delivery across the blood-brain barrier. Indeed, similar trends have been observed for immune checkpoint blockade agents, among many other drugs, making GBM one of the deadliest cancers.

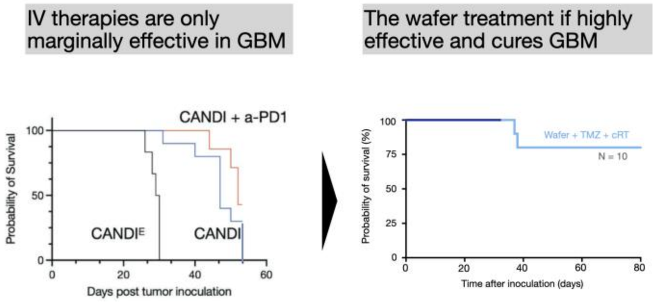

Fig. R1: IV-injected therapies done often do not work well in GBM because of delivery barriers (Nat Rev Canc 2020;20:26–41). The post-surgical wafer treatment, on the contrary, is highly effective as it promotes local drug release in the brain.

The novelty of the current study was that we developed a TAM-targeted immunomodulatory wafer, thus bypassing the delivery issue and, for the first time, showing that long-term modulation of phagocytic cells in the brain of resected tumors leads to actual cures. We are not aware of any other therapeutic approach that has achieved this complete cure in the very aggressive orthotopic CT2a and SB28 glioblastoma models.

We have added a new paragraph to address future mechanistic explorations:

Identifying the microanatomic biodistribution of immune-stimulating wafer materials in the immune hubs of the CNS, including the cervical lymph nodes and dura, is a future area of research that can provide a more thorough understanding of how these therapeutics function. In other cancer models, we have found that while polarizing macrophages locally can trigger strong anti-tumor functions, durable responses are linked to the trafficking of dendritic cells to draining lymphoid tissues. Determining whether and how wafer-based materials either passively enter or are actively trafficked into lymphoid tissues is our next step in advancing these intraoperative immunotherapies.

3. Minor Comments. The authors need to clarify the number of patient samples obtained/used for the study and whether there was variability among patient samples.
As requested, we have updated the number of patient samples and addressed their variability and comparability between samples.

Testing in human organotypic tissue slices
To determine the effects of wafer material on human GBM tissue, we studied freshly resected GBM sections (n=2). This study was approved by the Human Research Ethics Committee from the Geneva University in Switzerland (CCER 2022-02109 and 2023-01928). Informed consent was obtained from all patients who had been enrolled (n=2). Freshly resected GBM specimens were transferred from the operating room to the laboratory in a 50 mL conical tube containing ice-cold medium (High-glucose DMEM supplemented with HEPES 1X and 1X Pen-Strep solution). Pieces were punched from fresh tumor, embedded into 2% agarose, then sliced on vibratome to a thickness of 400

µm. GBM slices were cultured on a PTFE insert (Pore size 0.4 µm) in 24-well plates with culture media (Neurobasal medium supplemented with B27 1X, D-glucose 1.5 mg/mL, Glutamax 600 µM, L-glutamine 400 µM and 1X Pen-Strep solution). Tumor slices (n=5 per condition) were cultured for 34 days, and CANDI Wafer was performed on the 2nd day. CANDI Wafer (0.01 mg/mL, 0.1 mg/mL or 0.5 mg/mL) or not were added to the GBM slices for 72 hours in an incubation system (37°C, 5% CO_2). Supernatant was collected after 4, 24, 48 and 72 hours for IL-12p70 assay (n=5 per condition). Then, GBM slices were fixed in PFA 4% for immunohistofluorescence (n=3 per condition). The experiments yield comparable results between two patients, thus supporting the robustness and reproducibility of the findings.

Fig. 7: Locoregional effects of the wafer on human GBM. A. Fresh resected human glioblastoma tissue was cut into 400 µm-thick sections using a vibratome. Tissue slices were then incubated with CANDI450 wafer material. **B.** IL12 induction was measured as a function of dose and time (n=5 per condition). Note the highly significant IL12 induction in human GBM tissue within 24 hours ($p<0.001$) and the dose dependency. **C-D.** Immunohistochemistry performed in the same sections (n=3 per condition) showed upregulation of myeloid cell activation markers HLA-DR, CD68, and CD86. One-way ANOVA with multiple comparisons was used to determine statistical significance. For in vitro wafer effects on human monocyte/macrophage THP-1 cells, as well as toxicity testing, see **Fig. S20**.
